# Chemical Proteomics and Phenotypic Profiling Identifies the Aryl Hydrocarbon Receptor as a Molecular Target of the Utrophin Modulator Ezutromid

**DOI:** 10.1002/anie.201912392

**Published:** 2020-01-03

**Authors:** Isabel V. L. Wilkinson, Kelly J. Perkins, Hannah Dugdale, Lee Moir, Aini Vuorinen, Maria Chatzopoulou, Sarah E. Squire, Sebastian Monecke, Alexander Lomow, Marcus Geese, Philip D. Charles, Peter Burch, Jonathan M. Tinsley, Graham M. Wynne, Stephen G. Davies, Francis X. Wilson, Fraydoon Rastinejad, Shabaz Mohammed, Kay E. Davies, Angela J. Russell

**Affiliations:** ^1^ Department of Chemistry University of Oxford Chemistry Research Laboratory Mansfield Road Oxford OX1 3TA UK; ^2^ Department of Physiology, Anatomy and Genetics University of Oxford Sir Henry Wellcome Building of Gene Function South Parks Road Oxford OX1 3PT UK; ^3^ Evotec International GmbH Manfred Eigen Campus Essener Bogen 7 22419 Hamburg Germany; ^4^ Department of Biochemistry University of Oxford South Parks Rd Oxford OX1 3QU UK; ^5^ Summit Therapeutics plc. 136a Eastern Avenue, Milton Park Abingdon Oxfordshire OX14 4SB UK; ^6^ Target Discovery Institute University of Oxford Old Road Campus Roosevelt Drive Oxford OX3 7FZ UK; ^7^ Department of Pharmacology University of Oxford Mansfield Road Oxford OX1 3PQ UK

**Keywords:** mechanism of action, medicinal chemistry, photoaffinity labelling, proteomics, target identification

## Abstract

Duchenne muscular dystrophy (DMD) is a fatal muscle‐wasting disease arising from mutations in the dystrophin gene. Upregulation of utrophin to compensate for the missing dystrophin offers a potential therapy independent of patient genotype. The first‐in‐class utrophin modulator ezutromid/SMT C1100 was developed from a phenotypic screen through to a Phase 2 clinical trial. Promising efficacy and evidence of target engagement was observed in DMD patients after 24 weeks of treatment, however trial endpoints were not met after 48 weeks. The objective of this study was to understand the mechanism of action of ezutromid which could explain the lack of sustained efficacy and help development of new generations of utrophin modulators. Using chemical proteomics and phenotypic profiling we show that the aryl hydrocarbon receptor (AhR) is a target of ezutromid. Several lines of evidence demonstrate that ezutromid binds AhR with an apparent K_D_ of 50 nm and behaves as an AhR antagonist. Furthermore, other reported AhR antagonists also upregulate utrophin, showing that this pathway, which is currently being explored in other clinical applications including oncology and rheumatoid arthritis, could also be exploited in future DMD therapies.

## Graphical abstract

**Chemical proteomics** and phenotypic profiling illustrate the aryl hydrocarbon receptor (AhR) is a target of ezutromid, the first‐in‐class utrophin modulator for the treatment of Duchenne muscular dystrophy. Ezutromid binds to AhR with an apparent *K*_D_ of 50 nm and functions as an AhR antagonist. Other reported AhR antagonists also upregulate utrophin, showing that this pathway, which is currently being explored in other clinical applications, could also be exploited in future DMD therapies.

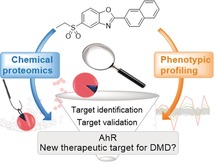

## Introduction

Duchenne muscular dystrophy (DMD) is a fatal, X‐linked muscle wasting disease that affects approximately 1 in 3500–5000 boys.^1^, ^2^ DMD is caused by loss‐of‐function mutations in the dystrophin gene. Thousands of mutations exist, with an estimated one‐third caused by spontaneous mutations.^3^, ^4^ Loss of dystrophin results in progressive muscle degeneration, with patients wheelchair‐dependent by their early teens and average life expectancy reduced to the late 20 s to 30 s, due to heart and respiratory failure.^5^, ^6^

Developments in the clinical standard of care have led to improvements in quality of life and longevity^7^, ^8^, ^9^ but there is currently no available cure for DMD. Several therapeutic strategies have reached the clinic, including stop codon readthrough and exon skipping which target particular mutations, and gene therapy which faces multiple challenges, including viral production and immune response.^10^, ^11^

Utrophin is an autosomal paralogue of dystrophin and highly similar in sequence, size and function.^12^ Increasing utrophin in the dystrophin‐deficient *mdx* mouse model^13^ prevents the dystrophic phenotype,^14^ not only offering a potential therapy to all DMD patients regardless of mutation type but also avoiding an immune response.^11^, ^15^

The first‐in‐class utrophin modulator ezutromid (formerly SMT C1100) **1** was discovered using a phenotypic screen for utrophin gene upregulation^16^, ^17^ and, after successful animal^18^ and human phase 1 trials,^19^, ^20^ progressed to an open‐label Phase 2 study in DMD patients (NCT02858362, Summit Therapeutics PLC). In January 2018, interim 24‐week data demonstrated reduced muscle fibre damage and increased levels of utrophin, providing the first evidence of ezutromid target engagement and proof of mechanism.^21^ However, these effects were not seen after the full 48 weeks of the trial, and thus development of ezutromid was discontinued. Since the mechanism of action of ezutromid is unknown, it is difficult to rationalise the lack of sustained clinical efficacy.

Phenotypic drug discovery is seeing a resurgence in popularity, and while the molecular target is unknown, when combined with target identification, it can lead to discovery of novel targets for a disease.^22^, ^23^ Target identification can also allow a rational compound design strategy, provide biomarkers for monitoring efficacy in clinical trials and provide insights into potentially deleterious on‐ and off‐target side effects.

Methods to elucidate target and mechanism of action of small molecules have been extensively reviewed.^24^, ^25^, ^26^, ^27^, ^28^, ^29^, ^30^ They are generally divided into (1) direct methods, which identify the protein interacting partner(s) of a compound, and (2) indirect phenotypic profiling methods, which reveal a compound's mechanism by comparing its effect on cell phenotype and gene expression with vehicle or inactive controls, and compounds with known mechanism. Both of these approaches were used to elucidate the target and mechanism of action of ezutromid.

## Results

### Probe design and validation

**Figure 1:**
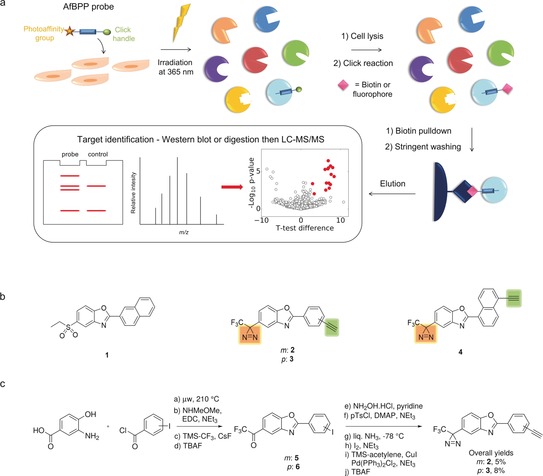
Design and synthesis of dual‐tagged ezutromid probes for use in affinity‐based protein profiling. a) Outline of the AfBPP strategy for target identification of small molecule probes in live cells. Probe‐treated cells are UV irradiated to trigger covalent binding of the probe to its protein target. A click reaction is performed to ligate a fluorophore or enrichment tag, such as biotin, to the probe. Enriched proteins are subsequently analysed using western blot or mass spectrometry. b) Chemical structures of ezutromid (**1**) ezutromid‐based active (**2**–**3**) and inactive (**4**) probes, containing a diazirine as a photoaffinity label (yellow shading) and alkyne click handles (green shading). c) Synthesis of probes **2** and **3**.

Affinity‐based protein profiling (AfBPP)^31^, ^32^, ^33^ is a chemical proteomics strategy to identify a molecule's direct target(s) within a cellular context. AfBPP requires development of active probes bearing tags or “click” handles for bioorthogonal tag ligation^34^ for enrichment by affinity chromatography. Enriched proteins can either be identified by western blotting or digested for LC‐MS/MS analysis (Figure 1 a). AfBPP can be enhanced by inclusion of a photoaffinity label on the probe to create a protein‐ligand covalent bond under UV irradiation, allowing discovery of low affinity binding partners.^35^

AfBPP was employed as a strategy for ezutromid, beginning with synthesis of active and inactive probes each containing a photoaffinity label and a click handle. A trifluoromethyl‐phenyl diazirine (TPD) was chosen to replace ezutromid's 5′ ethylsulfonyl moiety due to the similarity in sterics and electronics of the diazirine and the sulfonyl group. Alkynyl‐phenyl substituents (*meta* compound **2** and *para* compound **3**, Figure 1 b) were selected to replace the naphthyl substituent of ezutromid to provide a copper‐catalysed azide‐alkyne cycloaddition (CuAAC) click handle with minimal impact on the overall size of the compound. Altering the substitution pattern of ezutromid's naphthyl substituent from 2′ to 1′ has been previously shown to ablate activity,^17^ and was therefore incorporated into the design of the inactive control probe (compound **4**, Figure 1 b).

Synthesis of the dual‐tagged probes was undertaken in the same manner for each of the probes (Figure 1 c), beginning with microwave‐assisted benzoxazole cyclisation^17^ from 3‐amino‐4‐hydroxybenzoic acid and the corresponding acid chloride. EDC‐mediated couplings afforded Weinreb amides which were used to generate trifluoromethyl ketones **5** and **6** with the Ruppert‐Prakash reagent.^36^ Synthesis of TPDs from trifluoromethyl ketones is well‐established,^37^ starting from oxime formation and activation (*O*‐tosylation) followed by liquid ammonia induced diaziridine cyclisation and lastly oxidation to afford the diazirine. Sonagashira couplings with TMS‐acetylene followed by silyl deprotection furnished the final probes **2** and **3** in overall yields of 5–13 % (reported in the Supplementary methods).

**Figure 2:**
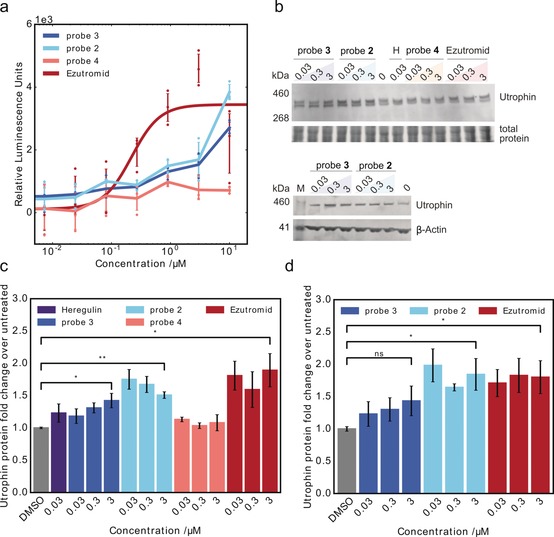
Biological activity of AfBPP probes **2**, **3** and **4**. a) Probes **2** and **3**, but not **4** show an increase in activity in a mouse H2K *mdx* cellular utrophin gene firefly luciferase reporter assay, although to a lesser extent than ezutromid, *n*=4, representative graph shown. b) Probes **2** and **3** increase utrophin protein, but inactive probe **4** does not. Representative utrophin western blots, top: probe treatment of mouse H2K *mdx* myoblasts, bottom: probe treatment of human DMD myoblasts. H, heregulin; M, protein standard (in kDa). Uncropped blots are presented in Supplementary Figure S4. c,d) Probes **2** and **3** increase utrophin protein levels comparable to heregulin and ezutromid after 24 h of compound treatment in mouse H2K *mdx* cells (c) and human DMD myoblasts (d) determined by western blot. Bars represent means of utrophin levels normalised to total protein and relative to DMSO, error bars are standard error of the mean, *n*=3. * *p*<0.05, ** *p*<0.005, ns=non‐significant.

Validation of individual probe bioactivity was performed using initial in vitro assessment of activity in a murine cellular utrophin gene firefly luciferase reporter assay.^17^ Probes **2** and **3** showed increases in luciferase activity in the micromolar range, but control probe **4** showed no increase in luciferase activity as expected (Figure 2 a). Next, fold change in utrophin protein levels post probe treatment compared to the vehicle control was evaluated by western blot analysis. Encouragingly, probes **2** and **3** were found to increase utrophin protein ≈1.5 fold, similar to other reported utrophin modulators^38^, ^39^ and the endogenous utrophin modulator heregulin.^40^, ^41^ Further, much of the activity of the parent compound ezutromid was retained in both mouse and human DMD cells (Figure 2 b–d). Probe **4** was confirmed to be inactive in all assays.

The fusion of myoblasts into mature myotubes is required during muscle development and repair, and is dysregulated in dystrophic muscles.^42^ Ezutromid and heregulin increased fusion of treated human DMD myocytes into mature myotubes in a concentration‐dependent manner (Supplementary Figure S1), indicating improved myogenesis.^43^ This behaviour was replicated in the *para* probe **3**, but not **2** or **4**, indicating conservation of physiological activity (Supplementary Figure S1c). On the basis of the combined activity data, **3** appears the most similar to ezutromid's phenotype and was taken forward to target identification studies.

**Figure 3:**
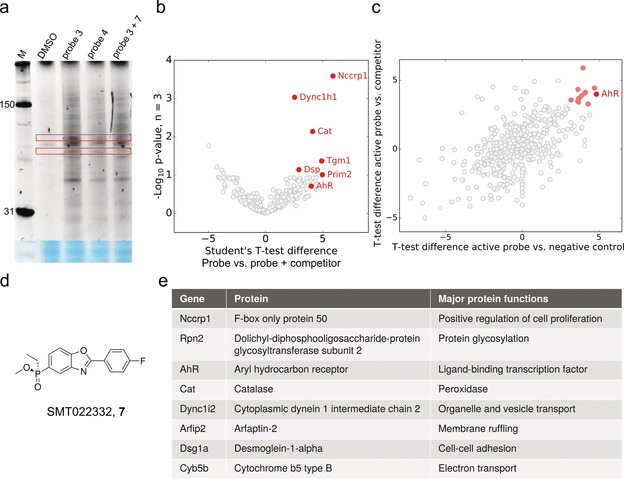
Target identification by chemical proteomics. a) In gel fluorescence of active probe **3**, inactive probe **4** and active **3** with competitor **7**, SMT022332 (d), labelled H2K *mdx* proteome. M, protein standard (in kDa); red boxes, bands that appear stronger in active probe treated samples than in the negative controls; blue stained gel represents total protein. b) Volcano plot of proteins enriched by probe **3** in the presence and absence of competitor **7**, top hits in red. c) Protein enrichment by probe **3** compared to inactive probe **4** and competitor **7**. Proteins strongly enriched by **3**, but not the controls, in red. d) Structure of competitor **7**, SMT022332. e) Top hits from (c), full list of proteins available in the supplementary information.

In addition to a negative control probe, a competition control was desired to facilitate deconvolution of therapeutically relevant targets from non‐specific or irrelevant hits. This competitor compound is ideally co‐treated at an excess concentration to occupy target binding sites and block binding of the photoaffinity probe. SMT022332 (probe **7**, Figure 3 d), an analogue of the second generation utrophin modulator SMT022357 whose ability to upregulate utrophin levels and diminish dystrophic pathology has been previously reported,^44^ was chosen as the competitor compound. SMT022332 (synthesis reported in Supplementary Figure S2a and supplementary methods) demonstrates an ability equivalent to ezutromid's in utrophin mRNA and protein upregulation (Supplementary Figure S2b,c). It also possesses significantly improved aqueous solubility, allowing dosage at the excess concentrations appropriate for competition experiments.

### Target identification

A chemical proteomics strategy to identify interacting proteins was employed using the active dual‐tagged probe **3**. This compound was used alongside two controls: (1) negative control probe **4** and (2) SMT022332 **7** dosed at >30× concentration to competitively block enrichment of **3**‐binding proteins.

Initially, the protein‐interaction profile of active probe **3** was assessed with gel‐based labelling experiments using live dystrophic mouse cells. Active probe **3** (3 μm), alongside DMSO vehicle, negative control dual tagged probe **4** (3 μm) and competitor **7** (100 μm) were dosed for 2 h. After irradiation (365 nm) and cell lysis, the probe‐labelled lysate was subjected to a CuAAC‐mediated click reaction with TAMRA‐azide, then SDS‐PAGE. In gel fluorescence showed probe‐dependent labelling of proteins (Figure 3 a), with little to no labelling in the vehicle (DMSO) control. Multiple proteins were labelled in the three conditions, with only a subset of proteins outcompeted by SMT022332, indicating a shortlist of specifically labelled targets amongst a majority of non‐specific hits. The labelling of proteins with probe **3** was also shown to be concentration‐dependent (Supplementary Figure S3).

Proteome‐wide target identification proceeded with click‐mediated ligation of probe‐labelled proteins to biotin, affinity enrichment with streptavidin beads and on‐bead tryptic digest. Samples were analysed by nano liquid chromatography‐tandem mass spectrometry (nanoLC‐MS/MS). Comparison of proteins enriched by active probe **3** but displaced in the presence of competitor SMT022332 gave a list of high occupancy binders (red proteins, Figure 3 b). Combining differences in proteins identified in active probe samples but not in the two controls delivered a shortlist of potential targets (Figure 3 c,e, full list of proteins identified available in Supplementary Information). To determine which potential targets should be prioritised in follow‐up studies, a phenotype profiling study of ezutromid was performed to identify pathways which are differentially regulated post compound exposure.

Ezutromid was profiled in a DiscoverX BioMAP Diversity PLUS study which examines changes in the activity of 148 biomarkers across 12 different human primary cell‐based co‐culture systems upon compound treatment^45^ (Figure S4). Ezutromid was found to dose‐dependently decrease activity of sIgG, and sIL‐17F, indicating suppression of the immune response. Meanwhile, ezutromid caused modest decreases in sPGE2, E‐selectin and IL1α, which are hallmarks of an anti‐inflammatory response. Ezutromid's profile most closely matched that of aryl hydrocarbon receptor (AhR) antagonist CH223191^46^ (Pearson correlation, *r*=0.854) from a database of >4500 reference compounds.

AhR is a pleiotropic ligand‐binding transcription factor which acts as an environmental sensor to control complex transcriptional programmes in a ligand‐, cell‐type‐ and context‐dependent manner. Interestingly, AhR was found to be one of the most differentially enriched proteins in the AfBPP LC‐MS/MS experiment, comparing the active probe to both inactive controls (Figure 3 c). Given the phenotypic profiling results which suggest ezutromid behaves as an AhR antagonist and the identification of AhR as a possible target in the chemical proteomics experiment, AhR was prioritised for follow‐up confirmatory and validation experiments.

### Target validation

**Figure 4:**
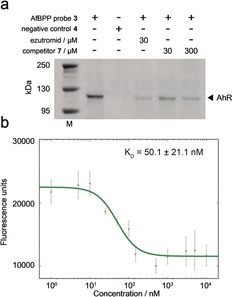
Target validation using immunoblotting and fluorescence. a) Immunoblot of AfBPP samples following treatment of human DMD myoblast cells and biotin enrichment. Selective enrichment of AhR by active probe **3**, but not inactive probe **4** is observed as well as enrichment competition by ezutromid and SMT022332 (**7**). M, protein standard (in kDa); uncropped blot is presented in Supplementary Figure 6. b) Fluorescence of ezutromid (100 nm, 313 nm excitation, 390 nm emission) measured after incubation (16 h) with AhR:ARNT (5–10000 nm).

Binding of AhR to probe **3**, but not negative control probe **4**, was verified in human DMD myoblasts by western blotting using an AhR‐specific antibody (Figure 4 a). The binding of both ezutromid and SMT022332 to human AhR in cellulo was confirmed by competition of the probe **3** affinity capture. Binding of ezutromid to a purified mouse AhR:ARNT complex (expression reported in the methods section, supplementary information) was investigated by adapting a previously reported fluorescence quenching assay.^47^, ^48^ This assay exploits ezutromid's intrinsic fluorescence in solution which is quenched upon protein binding and revealed a potent apparent K_D_ of 50.1±22.1 nm (Figure 4 b).

**Figure 5:**
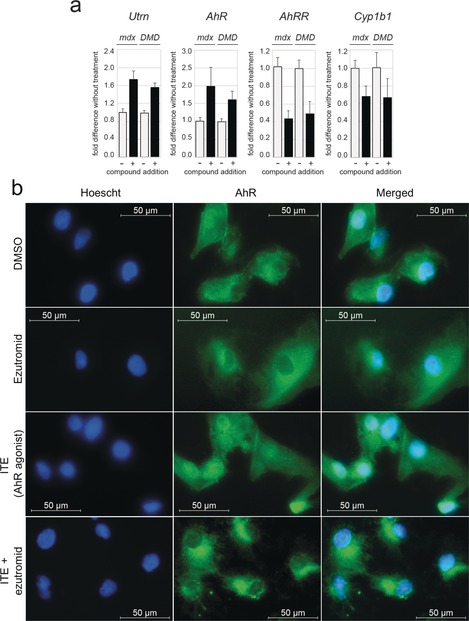
Ezutromid behaves as an aryl hydrocarbon receptor antagonist. a) Ezutromid treatment (+; 3 μm for 24 h; dark bars) increases full‐length utrophin (*Utrn*) and *AhR* mRNA expression in dystrophin‐deficient mouse (*mdx*) and human DMD myoblasts (*DMD*). Concomitantly, AhR‐responsive genes *AhRR* and *Cyp1b1* illustrate downregulation, indicating antagonism of AhR signalling with compound treatment. Bars represent mean fold change relative to DMSO (−; light bars) and normalised to S13 ± SD (*n*=3). Fold changes were all statistically significant with *p*<0.0001 (paired two‐tailed Student t‐test). b) Localisation of AhR induced by compound treatment determined by immunofluroscence. AhR is mostly located in the cytoplasm of human DMD myoblasts; ezutromid does not increase nuclear translocation, unlike agonist 2‐(1′*H*‐indole‐3′‐carbonyl)‐thiazole‐4‐carboxylic acid methyl ester (ITE), and co‐treatment of ITE with ezutromid results in retention of AhR in the cytoplasm.

AhR binds structurally diverse small molecule agonists and antagonists, with agonists inducing nuclear translocation of the receptor and increased transcription of AhR‐responsive genes. To ascertain whether ezutromid is an AhR agonist or antagonist, the effects of ezutromid on mRNA expression of AhR and AhR‐responsive genes Cyp1b1 and the AhR repressor (AhRR) were assessed in *mdx* mice and human DMD myoblasts by RT‐qPCR (Figure 5 a). Ezutromid treatment more than halved *AhRR* expression and led to an approximate 34 % decrease in *Cyp1b1* expression in both cell lines, indicating that ezutromid behaves as an AhR antagonist. Decreased AhR repressor production saw a concomitant 2.0‐ and 1.6‐ fold increase in AhR in *mdx* and human DMD myoblasts, respectively.

The ability of ezutromid to antagonise AhR activation was further investigated by immunofluorescence of AhR localisation after compound treatment. Human DMD cells were treated with ezutromid (3 μm, 2 h) and imaging with an AhR‐specific antibody revealed no induction of nuclear translocation, unlike that observed with the potent endogenous AhR agonist ITE (2‐(1′*H*‐indole‐3′‐carbonyl)‐thiazole‐4‐carboxylic acid methyl ester, 1 μm, 2 h),^49^, ^50^ which was used as a positive control (Figure 5 b). Co‐treatment of ezutromid with ITE led to retention of AhR in the cytoplasm (Figure 5 b), manifesting the ability of ezutromid to counteract a potent AhR agonist.

**Figure 6:**
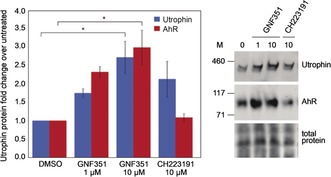
Differential effects of AhR antagonists on AhR and utrophin protein levels. The AhR antagonist GNF351 increases AhR and utrophin, while antagonist CH223191 increases utrophin without changing AhR, as determined by western blot analysis. Bars represent means of utrophin (blue) and AhR (red) levels normalised to total protein and relative to DMSO, error bars reflect standard error of the mean, *n*=3. * denotes *p*<0.05, M; protein standard (in kDa).

To determine whether AhR antagonism represents a viable mechanism to modulate utrophin in human DMD cells, AhR antagonists GNF‐351^51^ and CH223191 were assessed for their effect on utrophin protein levels by western blot (Figure 6). Gratifyingly, GNF‐351 treatment (24 h) showed a significant dose‐dependent 2.7‐fold increase in utrophin and a 3‐fold increase in AhR protein (both *p*<0.05). In contrast, treatment with antagonist CH223191 appeared to increase utrophin, but gave no change in AhR.

## Discussion

The utrophin modulator ezutromid was originally discovered using a phenotypic screen, and hence its molecular targets were unknown. Phenotypic screening for drug discovery has many advantages and disadvantages, but importantly, when it is combined with target identification, it can lead to the discovery of novel therapeutic mechanisms. This is particularly significant for diseases where no good targets are known. The identification and verification of AhR as a target for ezutromid represents the first validation of a disease‐modifying drug target in Duchenne muscular dystrophy. Our findings are also consistent with other reported observations. For example, resveratrol, which while exerting pleiotropic effects, has documented AhR antagonist activity^52^ and has been shown to increase utrophin expression and decrease inflammation in *mdx* mice.^53^ Furthermore, AhR agonist PCB‐126 has been shown to decrease utrophin mRNA expression in rat liver progenitor cells.^54^

A mechanism by which AhR antagonism modulates utrophin has not yet been established. However, prototypical AhR agonist TCDD has been shown to decrease activity and abundance of transcriptional coactivator peroxisome proliferator‐activated receptor‐γ coactivator‐1α (PGC1α),^55^ which stimulates utrophin expression at neuromuscular junctions.^56^ This dysregulation was removed by co‐treatment with an AhR antagonist, leading to stabilisation of active PGC1α. Treatment with AhR antagonists alone could lead to an increase in PGC1α and consequently utrophin.

Ezutromid is extensively metabolised in humans and, moreover, repeated dosing of ezutromid led to a reduction in exposure in both healthy volunteers and markedly in DMD patients in early phase clinical trials.^19^, ^57^ This phenomenon may account for the observed positive clinical effect followed by a lack of sustained efficacy in the Phase 2 clinical trial. An intriguing outstanding question is whether ezutromid's primary pharmacology also affects its overall exposure. In the short term, antagonism of AhR, which regulates the xenobiotic response, is likely to suppress ezutromid metabolism. However, we observe that ezutromid also increases AhR expression and decreases expression of the AhR repressor, and therefore chronic administration studies may provide insights into whether this impacts ezutromid metabolism in the longer term.

While numerous AhR agonists have been described, and are additionally found amongst existing pharmaceuticals,^58^ antagonists are fewer in number. Furthermore, AhR antagonists are typically free of the dioxin‐like carcinogenicity of some AhR agonists and are increasingly being investigated as possible therapeutic agents in a diverse range of indications including multiple myeloma,^59^ treatment‐resistant triple‐negative breast cancer,^60^ glioblastoma,^61^ head and neck squamous cell carcinoma,^62^ atheroscelerosis,^63^ obesity^64^ and rheumatoid arthritis.^65^ Furthermore, AhR antagonism has a promising application in stem cell expansion and development^66^, ^67^, ^68^ and cancer immunotherapy.^69^ While human and mouse AhR are known to have substantial differences in ligand specificity,^70^ in this work, ezutromid is shown to bind both species’ receptors. As a result, the identification of ezutromid as a novel, nontoxic AhR antagonist represents an important addition to the literature by expanding the toolbox available to interrogate AhR biology.

## Conclusion

In this work, the protein interactome of an ezutromid‐based probe has been profiled and the aryl hydrocarbon receptor has been identified and validated as a target through a combination of chemical proteomics and phenotypic profiling. Ezutromid binds AhR potently and is revealed as a novel AhR antagonist, inhibiting nuclear translocation and downregulating AhR responsive genes such as AhRR and Cyp1b1.

Signalling pathways connecting AhR to utrophin modulation now warrant further investigation given that known AhR antagonists also upregulate utrophin protein expression, suggesting AhR is a valid target for utrophin functional replacement therapies. This work could pave the way for the first target‐based drug discovery program in DMD as well as provide a biomarker for future clinical trials. Furthermore, AhR modulation could be used in combination with other therapeutic approaches, amplifying efficacy. Excitingly, the results of this work justifies research into a new approach to deliver a utrophin modulator to benefit all DMD patients.

## Conflict of interest

F.X.W. is employed by Summit Therapeutics plc. A.J.R., S.G.D., K.E.D and G.M.W. are minor shareholders of Summit Therapeutics plc.

## Supporting information

As a service to our authors and readers, this journal provides supporting information supplied by the authors. Such materials are peer reviewed and may be re‐organized for online delivery, but are not copy‐edited or typeset. Technical support issues arising from supporting information (other than missing files) should be addressed to the authors.

Supplementary
