# Supplementary material for: Chemical Proteomics and Phenotypic Profiling Identifies the Aryl Hydrocarbon Receptor as a Molecular Target of the Utrophin Modulator Ezutromid
Source: Angew Chem Int Ed Engl. 2020 Jan 3;59(6):2420–8. doi: 10.1002/anie.201912392 (PMC7003794; doi:10.1002/anie.201912392)
Supplement: Supplementary file 1 — Supplementary [file ANIE-59-2420-s001.pdf]

Angewandte Chemie
Eine Zeitschrift der Gesellschaft Deutscher Chemiker

Supporting Information

Chemical Proteomics and Phenotypic Profiling Identifies the Aryl Hydrocarbon Receptor as a Molecular Target of the Utrophin Modulator Ezutromid

*Isabel V. L. Wilkinson, Kelly J. Perkins, Hannah Dugdale, Lee Moir, Aini Vuorinen, Maria Chatzopoulou, Sarah E. Squire, Sebastian Monecke, Alexander Lomow, Marcus Geese, Philip D. Charles, Peter Burch, Jonathan M. Tinsley, Graham M. Wynne, Stephen G. Davies, Francis X. Wilson, Fraydoon Rastinejad, Shabaz Mohammed, Kay E. Davies, and Angela J. Russell**

anie_201912392_sm_miscellaneous_information.pdf

Supporting information

Experimental section
- Materials 2
- Cell Culture 2
- Utrophin Fluc reporter gene assay 2
- Utrophin Western blot assay 2
- DMD myoblast differentiation and fusion assay 3
- Chemoproteomics workflow of intact cell labelling followed by LC-MS/MS analysis 3
- Chemoproteomics followed by AhR immunoblotting 4
- AhR:ARNT expression 5
- AhR:ARNT fluorescence quenching assay 5
- RT-qPCR 5
- AhR localisation immunofluorescence 6
- Statistics 6
- General and analytical procedures 7
- Experimental procedures and data for the synthesis of probes 2, 3, 4 and 7 7
- Chiral purification of probe 7 20
- Selected spectra 22

Supplementary figures S1-8 29

References 37

Experimental section

Materials

The following materials were obtained from commercial sources: biotin azide (Sigma #762024), TAMRA-biotin-azide (DC Biosciences #CCR-1048), TBTA (Cayman Chemical #18816), TCEP (Sigma #C4706), CH-223191 (Selleckchem #S7711), GNF351 (Sigma #182707), ITE (R&D systems #1803/10) and heregulin (Recombinant Human NRG1-beta 1/HRG1-beta 1 EGF Domain Protein, R&D systems #396-HB-050). Ezutromid was provided by Summit Therapeutics PLC.

Analytical procedures and chemical synthesis of **2**, **3**, **4**, and SMT022332 are described in the supplementary information.

Cell culture

H2K-*mdx*[1] and H2K-*mdx* utrnA-luc cells[2,3] were maintained in DMEM (Life Technologies) supplemented with 20% Fetal Bovine Serum (Life Technologies), 2% CEE (SLI), 2 mM L-Glutamine (Life Technologies), 1% Penicillin Streptomycin (Life Technologies) and 2 µg/500 ml Mouse Interferon-γ (Roche). Cells were maintained at 10% CO_2 at 33 °C.

Immortalised DMD myoblasts isolated from the Fascia lata muscle of a 10 year old male, del 52 DMD (KM571DMD10FL) were acquired through collaboration with Professor Vincent Mouly (Insitut de Myologie, Paris). These were cultured in Skeletal Muscle Cell Growth Medium and Supplement (PromoCell C-23060), 20% Fetal Bovine Serum (Life Technologies) and 1% Penicillin Streptomycin (Life Technologies). Cells were maintained at 5% CO_2 at 37 °C.

Utrophin FLuc reporter gene assay

White flat bottomed 96 well plates (Corning) were seeded with 5000 H2K *mdx* utrnA-luc cells. After 24 h, cells were dosed with compound in triplicate, in the following concentration series: 0.01, 0.03, 0.1, 0.3, 1.0, 3.0, 10.0 µM from 10 mM solution stocks in DMSO (final DMSO concentration was 0.3%). The cells were incubated for a further 24 h, (10% CO_2, 33 °C). Relative luminescence readout after using the Luciferase Assay System (Promega, E1500) reagents was measured using a FLUOstar Optima plate reader (BMG Labtech). The means from the biological triplicates were fitted with a four parameter logistic function with least squares regression (Levenberg-Marquardt algorithm) to calculate EC_{50} values.

Utrophin Western blot assay

6 well plates were seeded with H2K *mdx* cells (1×10^5) per well. After 24 h (10% CO_2, 33 °C), the cells were dosed with query compounds in three concentrations from 10 mM solution stocks in DMSO (0.3% final DMSO concentration) in triplicate. Heregulin at 30 nM was used as a positive control. After 24 h, the cells were harvested (TrypLE Express, Gibco), washed (PBS) and lysed (RIPA buffer and protease inhibitors, Sigma #P8340). Protein content was quantified by a bicinchinonic acid protein assay (Thermo Scientific Pierce). For the Western blot, 30 µg lysate was separated by NuPAGE™ 3-8% Tris-Acetate protein gel electrophoresis

and transferred to a PVDF membrane (GE Healthcare). Utrophin protein was detected using Mancho-3 antibody (1: 50, kind gift from G.E. Morris, Oswestry, UK) and an AlexaFluor™ 680 anti-mouse antibody (1:10,000, Invitrogen). Blots were imaged with a Licor Odyssey system and relative protein quantitation was performed using Image Studio Lite. REVERT™ total protein stain (Licor) was used as a loading control, along with β-actin detected with anti-β-actin antibody (1: 20,000, Cell Signaling Technology #3700S) and an AlexaFluor™ 680 anti-mouse antibody (1:10,000, Invitrogen). Experiments were carried out at least three times.

DMD myoblast differentiation and fusion assay

Human DMD cells were seeded in black 384 well µclear cell culture plates and cultured in a differentiation medium containing DMEM/F-12 (Life Technologies), 5% KnockOut™ Serum Replacement (Life Technologies), 1 µM dexamethasone and 1% Penicillin Streptomycin (Life Technologies). The cells were dosed with compound (n = 4, 0.1 % final DMSO concentration) in differentiation medium on days 0 and 3. On day 5, the cells were fixed (4% paraformaldehyde in PBS, 15 min), permeabilised (0.5% Triton X-100 in PBS), blocked (5% FCS, 0.1% Tween-20 in PBS, 30 min) and stained with CellMask blue (Thermo Fisher). The cells were probed with anti-myosin heavy chain antibody (1:800, R&D #MAB4470), anti-mouse AlexaFluor™ 647 (1:500, Thermo Fisher #A21242) and DAPI. The cells were imaged using a Perkin Elmer Operetta high-content analysis system using a 10 × objective. The % fusion index was calculated from nuclei detected in MHC positive area divided by all detected nuclei in four image fields per well.

Chemoproteomics workflow of intact cell labelling followed by LC-MS/MS analysis

H2K *mdx* cells were seeded in 12 × 10 cm diameter dishes and grown to 80% confluency. Cells were treated with 3 µM probe **3** (with and without 100 µM competitor **7**), 3 µM probe **4** and the DMSO vehicle, in triplicate for each condition, to a final DMSO concentration of 0.3% in serum-free media, for 2 h at 33 °C and 10% CO_2. The cells were then washed with 1 × PBS and irradiated (3 min, 5 cm distance, 365 nm 100W lamp, VWR 36595-021) in serum-free media. The cells were washed with 1 × PBS then lysed for 10 min at 4 °C with 300 µL of a buffer containing 1% SDS, 1% triton X-100 and protease inhibitors (Sigma #P8340) in 1 × PBS. The dishes were scraped to collect the lysate, which was sonicated (4 × 2 s on, 3 s off, 20% amplitude) and the total protein concentration determined by a bicinchinonic acid protein assay (Thermo Scientific Pierce). 350 µg of lysate at 0.8 mg/mL concentration per sample was then Clicked to biotin-azide (FAC of 0.1 mM from 10 mM in DMSO stock) with $CuSO_4$ (FAC of 1 mM, from 50 mM in H_2O stock), TCEP (FAC of 1 mM, from 50 mM in H_2O stock) and TBTA (FAC of 0.1 mM, from 10 mM in DMSO stock) for 1 h at rt, 1000 rpm shaking[5]. Excess Click reagents were removed by protein precipitation (MeOH/$CHCl_3$) and washing of the protein pellet (MeOH × 4). The proteins were then solubilized with 2% SDS in PBS before dilution to 0.5 mg/mL protein, 0.5% SDS using 1 × PBS. Streptavidin beads (15 µL per sample, Dynabeads™ MyOne™ T1, Invitrogen) were washed twice (1 × PBS) then added to the labelled lysate samples. After 16 h rocking (65 rpm) at 4 °C, the beads were washed (3 ×

1% SDS, 1% triton X-100 in PBS, 3 × 4 M urea in 50 mM ammonium bicarbonate buffer (AB), 3 × 6 M urea in 50 mM AB).

On-bead digestion of pulled down proteins was achieved using a Filter Aided Sample Preparation (FASP) protocol[6]. Briefly, Vivacon 500 filters (Sartorius, VN01H02 10 kDa/VNCT01) were washed with 0.1% trifluoroacetic acid in 50% acetonitrile. The beads were loaded on the filter in 8 M urea in 100 mM AB for 30 minutes at rt. On-bead proteins were reduced (10 mM TCEP, 30 minutes, rt), alkylated (50 mM chloroacetamide, 30 min, rt in the dark) and washed (2 × 1 M urea in 50 mM AB). The proteins were subjected to tryptic digestion (0.2 µg enzyme, Promega, 1 M urea in 50 mM AB) overnight at 37°C. Trypsinised peptides collected from the filtrate were dried and resuspended in 50 µL 5% formic acid and 5% DMSO.

LC-MS/MS analysis was carried out on an Ultimate 3000 ultra-HPLC system (Thermo Fisher) coupled to a QExactive mass spectrometer (Thermo Fisher). The peptides were trapped on a C18 PepMap100 pre-column (300 µm i.d. x 5 mm, 100 Å, Thermo Fisher) using solvent A (0.1% formic acid in water) at a pressure of 500 bar, then separated on an in-house packed analytical column (75 µm i.d. packed with ReproSil-Pur 120 C18-AQ, 1.9 µm, 120 Å, Dr. Maisch GmbH) using a linear gradient (length: 60 minutes, 15% to 35% solvent B (0.1% formic acid in acetonitrile), flow rate: 200 nl/min). Data were acquired in a data-dependent mode (DDA). Full scan MS spectra were acquired in the Orbitrap (scan range 350-1500 m/z, resolution 70000, AGC target 3×10^6, maximum injection time 50 ms). The 10 most intense peaks were selected for HCD fragmentation at 30% of normalised collision energy (resolution 17500, AGC target 5×10^4, maximum injection time 120 ms) with first fixed mass at 180 *m/z*.

Peptide identification and quantification were performed by MaxQuant (version 1.5.0.35i)[7]. MS spectra were searched against the *Mus musculus* UniProt Reference proteome (retrieved 12/01/17) alongside a list of common contaminants. The search results were filtered to a 1% false discovery rate (FDR) for proteins, peptides and peptide-spectrum matches (PSM). Protein intensity distributions were log2 transformed using Perseus (version 1.5.5.3) and missing values were imputed with an estimated background noise value. The three replicates for each condition were grouped and Student's *t*-test (s0 = 0, FDR = 0.05) performed between the active probe samples and the two controls (inactive probe and competitor). Results of this analysis were plotted using Python plotting library Matplotlib.

Chemoproteomics followed by AhR immunoblotting

Human DMD cells were seeded in 10 cm diameter dishes and grown to 80% confluency. Cells were treated with 3 µM probe **3** (with and without 100 µM competitor **7**), 3 µM probe **4** and the DMSO vehicle, in duplicate for each condition, to a final DMSO concentration of 1% in serum-free media, for 2 h at 37 °C and 5% CO_2. The cells were then irradiated and lysed as above. 500 µg of lysate at 1 mg/mL concentration per sample was then Clicked to TAMRA-biotin-azide (FAC of 0.1 mM from 10 mM in DMSO stock) as above. Excess Click reagents were removed by protein precipitation ($MeOH/CHCl_3$) and washing of the protein pellet (MeOH × 4). The proteins were then solubilized with 2% SDS in PBS before dilution to 1

mg/mL protein, 0.2% SDS using 1 × PBS. Streptavidin beads (30 µL per sample, Dynabeads™ MyOne™ T1, Invitrogen) were washed twice (1 × PBS) then added to the labelled lysate samples. After 16 h rocking (65 rpm) at 4 °C, the beads were washed as above. Proteins were eluted from the beads by heating at 95 °C for 5 min in 1 × Laemmli buffer, 50 mM DTT, 8 M urea in 50 mM AB. The eluted proteins were separated by NuPAGE™ 4-12% Bis-Tris protein gel electrophoresis and transferred to a PVDF membrane (Roche). AhR protein was detected with an anti-AhR antibody (1:5000 in 2% BLOT-QuickBlocker™ (#WB57) solution in PBST, Enzo BML-SA210) followed by an IRDye® 800CW anti-rabbit antibody (1: 5000, Licor). Blots were imaged with a Licor Odyssey system.

AhR:ARNT expression

The mAHR (residues 25-433) and mARNT (residues 82-464) proteins were co-expressed in E. coli, using an expression and purification strategy similar to the purification of related ARNT heterdimers (HIF-ARNT, NPAS1-ARNT, NPAS3-ARNT) described previously[8,9]. For AHR-ARNT, the ARNT protein construct was expressed using a pMKH vector which did not provide any affinity tag while the AHR protein was expressed simultaneously in the same cells (BL21-CodonPlus DE3-RIL competent cells, from Agilent Technologies, Santa Clara, CA, #230245) using the PSJ2 vector which produced a C-terminal histidine tagged protein. Bacterial cultures were grown at 37°C, induced at 16°C overnight using 0.2 mM IPTG. Cells were then harvested, lysed using sonication, centrifuged to remove the pellet, and the supernatant applied to a Ni-NTA resin following the manufacturer's recommended procedure. After extensive washing with buffer, the protein complex was eluted using buffer containing 250 mM imidazole, and the heterodimer further purified using a 1-meter Superdex-200) gel filtration column, which eluted at the expected position of the heterodimer. SDS-PAGE gels stained with coommassie blue showed both subunits were co-purified in 1:1 stoichiometric amounts.

AhR:ARNT fluorescence quenching assay

Fluorescence quenching of ezutromid upon binding recombinant AhR:ARNT was monitored using a fluorescence microplate reader (Tecan Spark, 313 nm excitation/390 nm emission). 100 nM compound was incubated with AhR:ARNT (various concentrations ranging 0 – 10 µM) for 18 h (4 °C) in black 96 well plates, with a buffer containing 20 mM Tris pH 8, 200 mM NaCl and 1% DMSO. Fluorescence emission was measured and the K_D calculated by fitting the curve with a four parameter logistic function.

RT-qPCR

For assessment of mouse and human total utrophin, AhR, AhRR and Cyp1b1 transcript levels, two separate vials of H2K *mdx* and human DMD cells (KM571DMD10FL) were seeded in duplicate at a density of 350,000 per 150 mm dish. After 24 h, cells were dosed with 3 µM ezutromid (final concentration 0.3% DMSO) with non-treated controls supplemented with 0.3% DMSO. RNA was extracted using TRIzol™ Reagent (Invitrogen) according to the manufacturer's instructions. 500 ng of RNA from each sample was used to generate cDNA with the QuantiTect Reverse Transcription kit (Qiagen). All RT-qPCR reactions were

amplified using the StepOne™ Real-Time Polymerase chain reaction system (Applied Biosystems) with Fast SYBR™ Green Master Mix (Thermo Fisher). Amplification was performed in n=3 using cDNA synthesised from individual dishes (n = 2) and an RNA mix (n = 3 total) using the following primers: Utrophin exon 7-9 (mouse: forward 5′-GGACCGATGGACTCG CGTTC-3′ reverse 5′- CTTGTCAGGGAGATGCACAGCAAC-3′, human: forward 5′-GAAGGCC TCACAGGAACATCACTG-3′ reverse 5′-TCCATCCACAATGTCAGTTCCCC3′), AhR (mouse: forward 5′-GCTACTCCACTTCAGCCACCCTCC-3′ 5′-GAACTGGTACCCCGATCCTCTTGTG-3′, human: forward primer 5′- GATAGCTACTCCACTTCAGCCACCATCC-3′ reverse 5′-CTCTCGTGCACAGCTCTGCTTCAG-3′), AhRR (mouse: forward 5′-CATGTGGATGACCGGCA GGAC-3′ reverse 5′-GCAAGCCCTTGCCTCCTTCTTG-3′, human: forward 5′-GGAGTGCACGTA CGCGGGCCGGAAG-3′, human reverse 5′-CAGCTTGGAGATGATGTCAGGCG-3′), Cyp1b1 (mouse forward 5′-GCCAGCCAGGACACCCTTTC-3′, mouse reverse primer 5′-GGCAGGTTGGGCTGGTCACT-3′, human forward primer 5′-GGAGAACGTACCGGCCACTAT CAC-3′, human reverse 5′-CACGACCTGATCCAATTCTGCC-3′) and S13 (mouse: forward 5′-CCCGAGGATCTCTACCATT-3′ reverse 5′-GCCACTAGACAGAGGCTGT-3, human: forward 5′- CTGATCTTCCTGAAGATCTCTACC, reverse 5′-GGCAGAGGCTGTAGATGATTCA-3′). Values obtained according to the 2-ΔΔCT method[4] were subject to tailed t-tests and P-values using Prism7 (GraphPad, La Jolla, USA).

AhR localisation immunofluorescence

Human DMD cells (3×10^4) were seeded on 18 mm coverslips. After 24 h (10% CO_2, 33 °C), the cells were dosed with ezutromid (3 µM), ITE (1 µM) or both (from 10 mM solution stocks in DMSO, 1% final DMSO concentration) in serum-free media alongside a vehicle control. After 2 h, the cells were fixed (4% paraformaldehyde in PBS, 15 min), permeabilised (0.1% Triton X-100 in PBS) and blocked (10% FBS, 0.1% Tween-20 in PBS, 1 h). The cells were probed with anti-AhR antibody (1:200, Invitrogen, #MA1-514), anti-mouse AlexaFluor™ 488 (1:2000, #A-11059, Invitrogen) and Hoescht 33342 (10 µM, Sigma # 14533). The coverslips were mounted onto slides with FluorSave™ reagent. The cells were imaged using an Evos fluorescence microscope with a 40 × objective. Immunofluorescence experiments were performed on different cell populations three times.

Statistics

Differences between group means were calculated by unpaired, two-tailed t-tests where p values ≤ 0.05 were considered statistically significant.

Chemistry Experimental

General Procedures:

Reactions were carried out under inert conditions using an atmosphere of nitrogen, anhydrous solvents and oven dried glassware, unless aqueous reagents were used or otherwise stated. Microwave reactions were carried out using a Biotage® Initiator Classic microwave synthesiser.

Anhydrous solvents were dried by passing over an activated alumina column, under an inert atmosphere, using a solvent purification system. Water was purified by an Elix® UV-10 system. All other solvents and reagents were used as supplied (analytical or HPLC grade) without prior purification. PE refers to the fraction of petroleum ether boiling in the range 30-40 °C. rt refers to room temperature. Aqueous solutions of ammonium chloride (NH_4Cl) and sodium bicarbonate ($NaHCO_3$) were saturated. Concentration of solvents *in vacuo* was achieved by rotary evaporation using a diaphragm pump. Purification by flash column chromatography was carried out using Kieselgel 60 and a positive solvent pressure.

Analytical procedures:

TLC was carried out using Merck Kieselgel 60 F254 plates which were visualised using UV light (254 nm). Melting points were recorded on a EZ-Melt automated melting point apparatus. Fourier transform IR spectroscopy was carried out using a Bruker Tensor 27 FT-IR spectrometer as neat samples or thin films. Wavelengths of peak absorption are given in wavenumbers (cm^{-1}), with broad (br) signals indicated.

NMR spectra were recorded using Bruker Advance spectrometers (AVII 400 or AVII 500). ^1H NMR spectra were recorded at 298 K, locked to the relevant solvent standard. ^1H NMR and ^{13}C NMR data were recorded at 298 K, locked to the relevant solvent standard. NMR data are presented as: chemical shift δ (in ppm, $\delta_{TMS} = 0$), multiplicity (s = singlet, d = doublet, t = triplet, q =quartet, m = multiplet, br = broad, obs = obscured), coupling constants (J in Hz) and integration. Proton peak assignments were based on 1D data and COSY analysis and carbon peak assignments were based on 1D data and HSQC and HMBC analysis. Low resolution mass spectroscopy was carried out on an Agilent 6120 mass spectrometer. Accurate mass measurements were run on either a Bruker MicroTOF internally calibrated with polyalanine, or a Micromass GCT instrument fitted with a Scientific Glass Instruments BPX5 column (15 m × 0.25 mm) using amyl acetate as a lock mass. HR ESI and APCI were run on a Waters Acquity Ultraperformance LC system coupled to a Thermo Orbitrap Exactive MS. Optical rotations were recorded on a Perkin-Elmer PE241 polarimeter with a water-jacketed 1 cm cell. Specific rotations are reported in 10^{-1} deg cm^2 g^{-1} and concentrations in g/100 mL.

Chemical Experimental Procedures and Data:

General Procedure A:

Benzoxazole ring closure from a benzoic acid and 2-aminophenol derivative

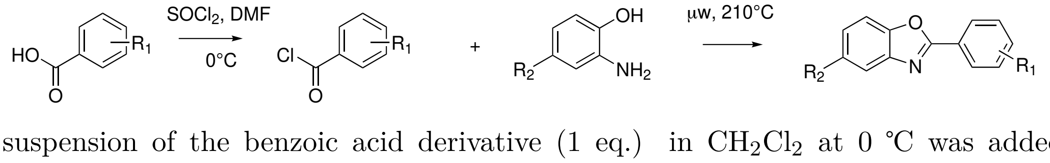

To a suspension of the benzoic acid derivative (1 eq.) in CH_2Cl_2 at 0 °C was added thionyl chloride (3 eq.) and dimethylformamide (cat.) The reaction mixture was warmed to rt and stirred for 16 h. When the reaction was complete, the reaction mixture was concentrated *in vacuo*, dissolved in Et_2O and filtered. The filtrate was concentrated *in vacuo* to yield the acid chloride, which was used immediately without further purification.

To a suspension of the 2-aminophenol derivative (1 eq.) in 1,4-dioxane was added a solution of the prepared acid chloride (1 eq.) in 1,4-dioxane at rt. The reaction vessel was heated under microwave activation at 210 °C for 15 min, then cooled to rt. The reaction mixture was poured into NaOH solution (1 M aq.), and extracted with EtOAc three times. The combined organic fractions were dried (Na_2SO_4) and concentrated *in vacuo* to yield the crude benzoxazole product.

General procedure B:

Formation of benzoxazole carboxamides

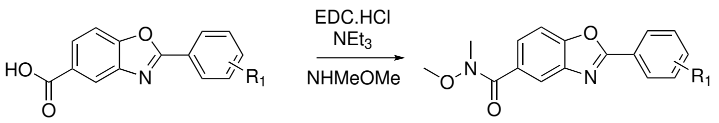

Acid benzoxazole derivatives were obtained by following General Procedure A, with no further purification. To a solution of the crude acid (1 eq.) in DMF was added EDC hydrochloride (2 eq.), *N*,*O*-dimethylhydroxylamine hydrochloride (2 eq.) and NEt_3 (2 eq.). The reaction mixture was stirred at rt for 16 h, then concentrated *in vacuo*. The residue was dissolved in EtOAc, then washed with citric acid solution (10% aq.), $NaHCO_3$ solution(10% aq.) and brine. The organic layer was concentrated *in vacuo* to yield the crude carboxamide product.

General procedure C:

Formation of trifluoromethylketone oximes from carboxamides

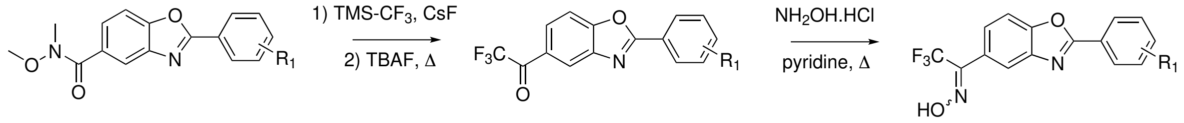

Adapting a known procedure,[10] caesium fluoride (0.2 eq.) and trifluoromethyltrimethylsilane (2 eq.) were added to a solution of Weinreb amide derivative (1 eq.) in toluene. The reaction mixture was stirred at rt with further equivalents of caesium fluoride and trifluoromethyltrimethylsilane added every 24 h until the reaction was complete by TLC. At this point, the reaction was concentrated *in vacuo* and the residue dissolved in THF. TBAF (1.2 eq., 1 M in THF) was added dropwise and the reaction stirred at rt for 3 h at 50 °C. The reaction was cooled to rt, diluted with Et_2O and washed with water and brine. The organic layer was dried (Na_2SO_4) and concentrated *in vacuo* to yield the crude trifluoromethyl ketone product. The crude was filtered through a silica plug, eluting with PE : EtOAc (2:1), and concentrated *in vacuo*.

To a solution of the prepared crude trifluoromethylketone derivative (1 eq.) in pyridine (excess) and ethanol over molecular sieves (3 Å) was added hydroxylammonium chloride (3 eq.). The reaction mixture was heated under reflux for 16 h, then concentrated *in vacuo*. The residue was dissolved in EtOAc, washed with brine/water (1:1), dried (Na_2SO_4) and concentrated *in vacuo* to yield the crude oxime product.

General procedure D:

Formation of trifluoromethylketone tosyl oximes

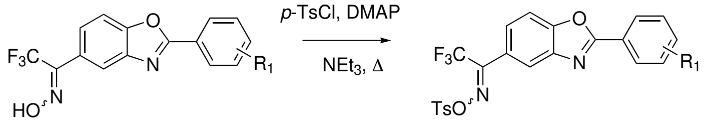

To a solution of oxime (1 eq.), 4-dimethylaminopyridine (0.1 eq.) and triethylamine (3 eq.) in CH_2Cl_2 was added *p*-toluenesulfonyl chloride (2 eq., recrystallised from PE) portionwise. The reaction mixture was stirred at 40 °C for 16 h, then cooled to rt. The mixture was diluted with CH_2Cl_2, washed with water then brine, dried (Na_2SO_4) and concentrated *in vacuo* to yield the crude tosyl oxime products in a mixture of E/Z isomers.

General procedure E:

Formation of the diaziridine

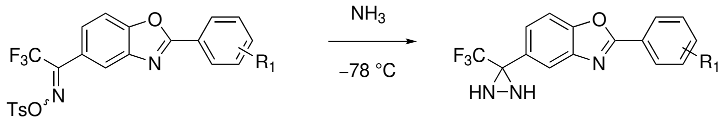

NH_3 (excess) was condensed into a solution of tosyl oxime (1 eq.) in CH_2Cl_2 at −78 °C. The reaction mixture was stirred and warmed to rt over 6 h, then concentrated *in vacuo* to yield the crude diaziridine product.

General procedure F:

Formation of the diazirine

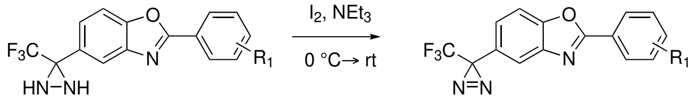

To a solution of diaziridine (1 eq.) and triethylamine (3 eq.) in CH_2Cl_2 was added iodine (1.2 eq.) portionwise. The reaction mixture was covered in foil to exclude light and stirred for 15 min at rt. The mixture was then concentrated *in vacuo* to yield the crude diazirine product.

General procedure G:

Sonagashira coupling of aryl halide with TMS-acetylene

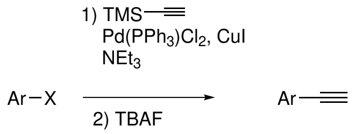

To a solution of aryl halide (1 eq.), bis(triphenylphosphine)palladium(II) dichloride (0.15 eq.) and copper (I) iodide (0.1 eq.) in THF was added NEt_3 (excess) and trimethylsilylacetylene (1 eq.) dropwise. The reaction mixture was degassed, put under a N_2 atmosphere, and stirred at 70 °C until the reaction was complete by TLC. The solvent was removed *in vacuo* and the residue purified by flash column chromatography. The isolated TMS-alkyne intermediate was dissolved in THF and TBAF (1.5 eq., 1 M in THF) was added dropwise. The reaction was stirred at rt until completion. The reaction mixture was diluted with EtOAc, washed with water and the phases separated. The organic layer was dried (Na_2SO_4) and concentrated *in vacuo* to yield the crude alkyne product.

2-(3-Iodophenyl)-*N*-methoxy-*N*-methylbenzo[*d*]oxazole-5-carboxamide 9

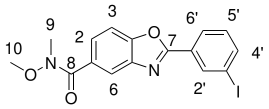

Carboxamide **9** was obtained according to General Procedure B, using 3-iodobenzoic acid (486 mg, 1.96 mmol), thionyl chloride (0.43 mL, 5.88 mmol) and DMF (0.10 mL) in CH_2Cl_2 (20 mL). The acid chloride intermediate formed was reacted with 3-amino-4-hydroxybenzoic acid (300 mg, 1.96 mmol) in 1,4-dioxane (5 mL). Amide coupling was performed on the crude product using EDC hydrochloride (752 mg, 3.92 mmol), *N,O*-dimethylhydroxylamine hydrochloride (382 mg, 3.92 mmol) and NEt_3 (0.60 mL, 4.31 mmol) in DMF (25 mL). The crude product was purified by flash column chromatography (PE : EtOAc, 7:3) to yield carboxamide **9** (392 mg, 0.960 mmol, 49%) as a white solid.

$\mathbf{R_f}$ 0.21 (PE : EtOAc, 7:3); $\mathbf{v_{max}}$ (cm^{-1}) 1635 ((C=O)NH), 1450, 1107 (C-N), 1085; **mp** 113-115 °C; **^{1}HNMR** (400 MHz, $CDCl_3$) δ_H 8.62 (1H, dd, J = 1.3, 1.3 Hz, $H_{2'}$), 8.22 (1H, ddd, J = 7.8, 1.3, 1.3 Hz, $H_{6'}$), 8.15 (1H, d, J = 1.7 Hz, H_6), 7.88 (1H, 1H, ddd, J = 7.8, 1.3, 1.3 Hz, $H_{4'}$), 7.77 (1H, dd, J = 8.5, 1.7 Hz, H_2), 7.61 (1H, d, J = 8.5 Hz, H_3), 7.27 (1H, dd, J = 7.8, 7.8 Hz, $H_{5'}$), 3.57 (3H, s, H_{10}), 3.41 (3H, s, H_9); **^{13}CNMR** (125 MHz, $CDCl_3$) δ_C 169.2 (C_8), 162.5 (C_7), 152.1 (C_4), 141.6 ($C_{4'}$), 140.8 (C_5), 136.6 ($C_{2'}$), 131.0 (C_1), 130.7 ($C_{5'}$), 128.8 ($C_{1'}$), 126.9 ($C_{6'}$), 126.6 (C_2), 120.8 (C_6), 110.5 (C_3), 94.5 ($C_{3'}$), 61.3 (C_{10}), 33.9 (C_9); **HRMS** (APCI$^+$) Calc. for $C_{16}H_{14}IN_2O_3$ $[M+H]^+$ 409.0043, found 409.0035.

2,2,2-Trifluoro-1-(2-(3-iodophenyl)benzo[*d*]oxazol-5-yl)ethan-1-one oxime 10

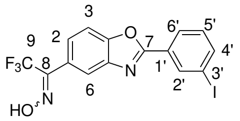

Oxime **10** was obtained according to General Procedure C, using carboxamide **9** (1.45 g, 3.55 mmol), trifluoromethyltrimethylsilane (1.05 mL, 7.10 mmol) and caesium fluoride (108 mg, 0.710 mmol) in toluene (40 mL). The reaction was stirred for three days, with further trifluoromethyltrimethylsilane (1.05 mL, 7.10 mmol) and caesium fluoride (108 mg, 0.710 mmol) added every 24 h. TBAF (4.26 mL, 1 M in THF) was used for desilylation of the intermediate formed. The crude trifluoromethylketone product was dissolved in pyridine (5 mL) and ethanol (25 mL) and refluxed with hydroxylammonium chloride (740 mg, 10.7 mmol). The crude product was purified by flash column chromatography (PE : EtOAc, 9:1) to yield oxime **10** (358 mg, 0.828 mmol, 23%) as a white solid.

$\mathbf{R_f}$ 0.28 (PE : EtOAc, 9:1); $\mathbf{v_{max}}$ (cm^{-1}) 1547, 1471, 1250 (C-F), 1151 (C-F), 973 (N-O); **mp** 182-184 °C; **^{1}HNMR** (500 MHz, CD_3OD) δ_H 8.60 (1H, dd, J = 1.6, 1.6 Hz, $H_{2'}$), 8.25 (1H, ddd, J = 7.8, 1.6, 1.0 Hz, $H_{6'}$), 7.97 (1H, ddd, J = 7.8, 1.6, 1.0 Hz, $H_{4'}$), 7.86 (1H, s, H_6), 7.80 (1H, d, J = 8.5 Hz, H_3), 7.53 (1H, dd, J = 8.5, 1.3 Hz, H_2), 7.36 (1H, dd, J = 7.8, 7.8 Hz, $H_{5'}$); **^{13}CNMR** (125 MHz, $CDCl_3$) δ_C 163.8 (C_7), 152.7 (C_4), 146.5 (q, $^2J_{C\text{-}F}$ = 32.6 Hz, C_8), 142.9 (C_5), 142.2 ($C_{4'}$), 137.5 ($C_{2'}$), 132.0 ($C_{5'}$), 129.7 ($C_{1'}$), 127.9 ($C_{6'}$), 127.8 (C_2), 125.5 (C_1), 122.5 (q, $^1J_{C\text{-}F}$ = 272.5 Hz, C_9), 121.8 (C_6), 112.1 (C_3), 95.1 ($C_{3'}$); **^{19}FNMR** (376 MHz, $CDCl_3$) δ_F −66.5 (s); **HRMS** (ES$^+$) Calc. for $C_{15}H_7F_3IN_2O_2$ $[M-H]^-$ 430.9510, found 430.9513.

2,2,2-Trifluoro-1-(2-(3-iodophenyl)benzo[*d*]oxazol-5-yl)ethan-1-one *O*-tosyl oxime 11

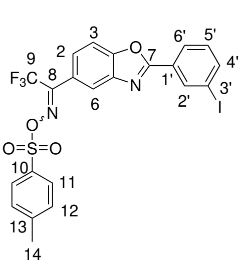

Tosyl oxime **11** was obtained according to General Procedure D, using oxime **10** (358 mg, 0.828 mmol), *p*-toluenesulfonyl chloride (316 mg, 1.66 mmol), 4-dimethylaminopyridine (10.1 mg, 82.8 µmol) and NEt_3 (0.35 mL, 2.48 mmol) in CH_2Cl_2 (20 mL). The crude product was purified by flash column chromatography (PE : EtOAc, 37:3) to yield tosyl oxime **11** (402 mg, 0.685 mmol, 83%) as a pale yellow solid in a mixture of isomers (7:1).

R_f 0.24 (PE : EtOAc, 9:1); **v_{max}** (cm^{-1}) 2932 (C-H), 1622 (C=N-O), 1391 (S=O), 1250 (C-F), 1181 (S=O), 1149; **mp** 229-231 °C; **^{1}HNMR** (500 MHz, $CDCl_3$) δ_H 8.64 (1H, dd, J = 1.6, 1.6 Hz, $H_{2'}$), 8.25 (1H, ddd, J = 7.9, 1.6, 1.0 Hz, $H_{6'}$), 7.94 (1H, ddd, J = 7.9, 1.6, 1.0 Hz, $H_{4'}$), 7.93 (2H, d, J = 8.4 Hz, H_{11}), 7.82 (1H, d, J = 1.7 Hz, H_6), 7.72 (1H, d, J = 8.5 Hz, H_3), 7.46 (1H, dd, J = 8.5, 1.7 Hz, H_2), 7.43 (2H, d, J = 8.4 Hz, H_{12}), 7.32 (1H, dd, J = 7.9, 7.9 Hz, $H_{5'}$), 2.52 (3H, s, H_{14}); **^{13}CNMR** (125 MHz, $CDCl_3$)δ_C 162.9 (C_7), 153.5 (q, $^2J_{C\text{-}F}$ = 33.7 Hz, C_8), 152.3 (C_4), 146.3 (C_{13}), 142.2 (C_5), 141.0 ($C_{4'}$), 136.6 ($C_{2'}$), 131.1 (C_{10}), 130.7 ($C_{5'}$), 129.9 (C_{12}), 129.3 (C_{11}), 128.2 ($C_{1'}$), 127.0 ($C_{6'}$), 126.0 (C_2), 121.2 (C_1), 121.0 (C_6), 119.6 (q, $^1J_{C\text{-}F}$ = 277.6 Hz, C_9), 111.5 (C_3), 94.4 ($C_{3'}$), 21.8 (C_{14}); **^{19}FNMR** (376 MHz, $CDCl_3$) δ_F −66.7 (s); **HRMS** (CI^+) Calc. for $C_{22}H_{15}F_3IN_2O_4$ $[M+H]^+$ 586.9744, found 586.9759.

2-(3-Iodophenyl)-5-(3-(trifluoromethyl)diaziridin-3-yl)benzo[*d*]oxazole 12

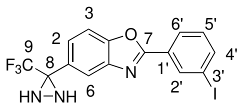

Diaziridine **12** was obtained according to General Procedure E, using tosyl oxime **11** (375 mg, 0.640 mmol) and NH_3 (5 mL) in CH_2Cl_2 (5 mL). The crude product was purified by flash column chromatography (PE : EtOAc, 4:1) to yield diaziridine **12** (270 mg, 0.626 mmol, 98%) as a white solid.

R_f 0.16 (PE : EtOAc, 9:1); **v_{max}** (cm^{-1}) 3243 (N-H), 1570 (N-H), 1216 (C-F), 1150; **mp** 128-130 °C; **^{1}HNMR** (500 MHz, $CDCl_3$) δ_H 8.62 (1H, dd, J = 1.7, 1.7 Hz, $H_{2'}$), 8.22 (1H, ddd, J = 7.9, 1.7, 1.1 Hz, $H_{6'}$), 8.06 (1H, d, J = 1.6 Hz, H_6), 7.90 (1H, ddd, J = 7.9, 1.7, 1.1 Hz, $H_{4'}$), 7.66 (1H, dd, J = 8.5, 1.6 Hz, H_2), 7.63 (1H, d, J = 8.5 Hz, H_3), 7.28 (1H, dd, J = 7.9, 7.9 Hz, $H_{5'}$), 2.89 (1H, d, J = 8.7 Hz, -NH), 2.32 (1H, d, J = 8.7 Hz, -NH); **^{13}CNMR** (125 MHz, $CDCl_3$) δ_C 162.6 (C_7), 151.6 (C_4), 142.2 (C_5), 140.8 ($C_{4'}$), 136.5 ($C_{2'}$), 130.6 ($C_{5'}$), 128.7 (C_1), 128.5 ($C_{1'}$), 126.9 ($C_{6'}$), 125.5 (C_2), 123.5 (q, $^1J_{C\text{-}F}$ = 278.2 Hz, C_9), 120.6 (C_6), 111.1 (C_3), 94.4 ($C_{3'}$), 58.2 (q, $^2J_{C\text{-}F}$ = 36.2 Hz, C_8); **^{19}FNMR** (376 MHz, $CDCl_3$) δ_F −75.5 (s); **HRMS** (ES^+) Calc. for $C_{15}H_{10}F_3IN_3O$ $[M+H]^+$ 431.9815, found 431.9815.

2-(3-Iodophenyl)-5-(3-(trifluoromethyl)-3*H*-diazirin-3-yl)benzo[*d*]oxazole 13

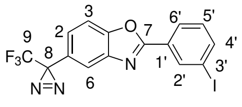

Diazirine **13** was obtained according to General Procedure F, using diaziridine **12** (250 mg, 0.580 mmol), iodine (176 mg, 0.696 mmol) and NEt_3 (0.24 mL, 1.74 mmol) in CH_2Cl_2 (15 mL). The crude product was purified by flash column chromatography (PE : EtOAc, 19:1 → 9:1) to yield diazirine **13** (242 mg, 0.564 mmol, 97%) as a white solid.

R_f 0.81 (PE : EtOAc, 9:1); **v_{max}** (cm^{-1}) 1547, 1254 (C-F), 1194, 1154 (C-F), 723; **mp** 101-103 °C; **^{1}HNMR** (500 MHz, $CDCl_3$) δ_H 8.60 (1H, t, J = 1.7 Hz, $H_{2'}$), 8.21 (1H, ddd, J = 7.9, 1.7, 1.0 Hz, $H_{6'}$), 7.90 (1H, ddd, J = 7.9, 1.7, 1.0 Hz, $H_{4'}$), 7.72 (1H, d, J = 1.8 Hz, H_6), 7.61 (1H, d, J = 8.6 Hz, H_3), 7.28 (1H, t, J = 7.9 Hz, $H_{5'}$), 7.20 (1H, dd, J = 8.6, 1.8 Hz, H_2); **^{13}CNMR** (125 MHz, $CDCl_3$) δ_C 162.9 (C_7), 151.5 (C_4), 142.6 (C_5), 141.0 ($C_{4'}$), 136.7 ($C_{2'}$), 130.8 ($C_{5'}$), 128.5 ($C_{1'}$), 127.0 ($C_{6'}$), 126.0 (C_1), 124.1 (C_2), 122.3 (q, $^1J_{C\text{-}F}$ = 274.7 Hz, C_9), 119.5 (C_6), 111.5 (C_3), 94.6 ($C_{3'}$), 28.7 (q, $^2J_{C\text{-}F}$ = 40.6 Hz, C_8); **^{19}FNMR** (376 MHz, CD_3OD) δ_F −67.4 (s); **HRMS** (ES^+) Calc. for $C_{15}H_8F_3IN_3O$ $[M+H]^+$ 429.9659, found 429.9655.

2-(3-Ethynylphenyl)-5-(3-(trifluoromethyl)-3*H*-diazirin-3-yl)benzo[*d*]oxazole 2

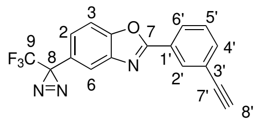

Diazirine **2** was obtained according to General Procedure G, using diazirine **13** (100 mg, 0.233 mmol), bis(triphenylphosphine)palladium(II) dichloride (24.5 mg, 35.0 µmol), copper(I) iodide (4.44 mg, 23.3 µmol), NEt_3 (0.70 mL) and trimethylsilylacetylene (40 µL, 0.280 mmol) in THF (10 mL). The reaction was complete after 1 h. The TMS-protected alkyne intermediate formed was purified by flash column chromatography (PE : EtOAc, 1:0 → 99:1), then deprotected with TBAF (0.70 mL, 1 M in THF) in THF (5 mL). The reaction was complete after 30 min. The crude product was purified by flash column chromatography (PE : EtOAc, 1:0 → 49:1) to yield alkyne **2** (53.2 mg, 0.163 mmol, 70%) as a white solid.

$\mathbf{R_f}$ 0.62 (PE : EtOAc, 49:1); $\mathbf{v_{max}}$ (cm^{-1}) 3310 (sharp, alkyne C-H), 1556, 1259 (C-F), 1197, 1154, 803; **mp** 105-107 °C; **^{1}HNMR** (500 MHz, $CDCl_3$) δ_H 8.40 (1H, dd, J = 1.7, 1.7 Hz, $H_{2'}$), 8.25 (1H, ddd, J = 7.9, 1.4, 1.4 Hz, $H_{6'}$), 7.75 (1H, d, J = 1.3 Hz, H_6), 7.70 (1H, ddd, J = 7.9, 1.4, 1.4 Hz, $H_{4'}$), 7.64 (1H, d, J = 8.6 Hz, H_3), 7.54 (1H, dd, J = 7.9, 7.9 Hz, $H_{5'}$), 7.24 (1H, dd, J = 8.6, 1.3 Hz, H_2), 3.20 (1H, s, $H_{8'}$); **^{13}CNMR** (125 MHz, $CDCl_3$) δ_C 163.6 (C_7), 151.4 (C_4), 142.6 (C_5), 135.4 ($C_{4'}$), 131.4 ($C_{2'}$), 129.1 ($C_{5'}$), 127.9 ($C_{6'}$), 126.9 ($C_{1'}$), 125.8 (C_1), 123.9 (C_2), 123.3 ($C_{3'}$), 122.2 (q, $^1J_{C\text{-}F}$ = 274.7 Hz, C_9), 119.3 (C_6), 111.3 (C_3), 82.4 ($C_{7'}$), 78.6 ($C_{8'}$), 28.7 (q, $^2J_{C\text{-}F}$ = 40.7 Hz, C_8); **^{19}FNMR** (376 MHz, CD_3OD) δ_F −67.4 (s); **HRMS** (ES^+) Calc. for $C_{17}H_9F_3N_3O$ $[M+H]^+$ 328.0692, found 328.0691.

2-(4-Iodophenyl)-*N*-methoxy-*N*-methylbenzo[*d*]oxazole-5-carboxamide 14

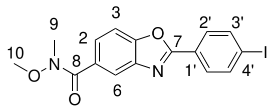

Carboxamide **14** was obtained according to General Procedure B, using 4-iodobenzoic acid (500 mg, 2.01 mmol), thionyl chloride (0.44 mL, 6.03 mmol) and DMF (0.10 mL) in CH_2Cl_2 (20 mL). The acid chloride intermediate formed was added to a solution of 3-amino-4-hydroxybenzoic acid (277 mg, 1.81 mmol, 0.9 eq.) in 1,4-dioxane (5 mL). Amide coupling was performed on the crude product using EDC hydrochloride (769 mg, 4.02 mmol), *N,O*-dimethylhydroxylamine hydrochloride (392 mg, 4.02 mmol) and NEt_3 (0.56 mL, 4.01 mmol) in DMF (25 mL). The crude product was purified by flash column chromatography (PE : EtOAc, 3:1) to yield carboxamide **14** (281 mg, 0.688 mmol, 38%) as a white solid.

$\mathbf{R_f}$ 0.11 (PE : EtOAc, 9:1); $\mathbf{v_{max}}$ (cm^{-1}) 2980 (C-H), 1636 ((C=O)NH), 1396, 1261, 1060; **mp** 143-144 °C; **^{1}HNMR** (400 MHz, $CDCl_3$) δ_H 8.13 (1H, d, J = 1.6 Hz, H_6), 7.95 (2H, m, $H_{2'}$), 7.87 (2H, m, $H_{3'}$), 7.75 (1H, dd, J = 1.6, 8.6 Hz, H_2), 7.58 (1H, d, J = 8.6 Hz, H_3), 3.55 (3H, s, H_{10}), 3.39 (3H, s, H_9); **^{13}CNMR** (125 MHz, $CDCl_3$) δ_C 169.2 (C_8), 163.4 (C_7), 152.1 (C_4), 141.7 (C_5), 138.4 ($C_{3'}$), 130.9 (C_1), 129.2 ($C_{2'}$), 126.5 (C_2), 126.3 ($C_{1'}$), 120.7 (C_6), 110.4 (C_3), 99.1 ($C_{4'}$), 61.2 (C_{10}), 33.9 (C_9); **HRMS** (ES^+) Calc. for $C_{16}H_{14}IN_2O_3$ $[M+H]^+$ 409.0043, found 409.0041.

2,2,2-Trifluoro-1-(2-(4-iodophenyl)benzo[*d*]oxazol-5-yl)ethan-1-one oxime 15

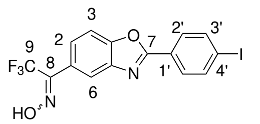

Oxime **15** was obtained according to General Procedure C, using carboxamide **14** (1.55 g, 3.81 mmol), trifluoromethyltrimethylsilane (1.13 mL, 7.62 mmol) and caesium fluoride (116 mg, 0.762

mmol) in toluene (50 mL). The reaction was stirred for three days, with further trifluoromethyltrimethylsilane (1.13 mL, 7.62 mmol) and caesium fluoride (116 mg, 0.762 mmol) added every 24 h. TBAF (4.57 mL, 1 M in THF) was used for desilylation of the intermediate formed. The crude trifluoromethylketone product was dissolved in pyridine (5 mL) and ethanol (25 mL) and refluxed after addition of hydroxylammonium chloride (794 mg, 11.4 mmol). The crude product was purified by flash column chromatography (PE : EtOAc, 9:1 → 4:1) to yield oxime **15** (527 mg, 1.22 mmol, 32%) as a pale green solid.

$\mathbf{R_f}$ 0.69 (PE : EtOAc, 4:1); $\mathbf{v_{max}}$ (cm^{-1}) 1476 (aromatic C=C), 1259 (C-F), 1151 (C-F), 970 (N-O); **mp** 197-199 °C; **^{1}HNMR** (400 MHz, $CDCl_3$) δ_H 7.97 (2H, m, $H_{3'}$), 7.95 (1H, d, J = 1.2 Hz, H_6), 7.91 (2H, m, $H_{2'}$), 7.68 (1H, d, J = 8.4 Hz, H_3), 7.51 (1H, dd, J = 8.4, 1.2 Hz, H_2); **^{13}CNMR** (125 MHz, $CDCl_3$) δ_C 165.1 (C_7), 152.9 (C_4), 145.1 (q, $^2J_{C\text{-}F}$ = 32.6 Hz, C_8), 143.3 (C_5), 139.9 ($C_{3'}$), 130.4 ($C_{2'}$), 128.0 (C_1), 127.5 (C_2), 125.8 ($C_{1'}$), 121.9 (C_6), 120.9 (q, $^1J_{C\text{-}F}$ = 271.9 Hz, C_9), 112.3 (C_3), 100.3 ($C_{4'}$); **^{19}FNMR** (376 MHz, $CDCl_3$) δ_F −67.5 (s); **HRMS** (ES$^+$) Calc. for $C_{15}H_7F_3IN_2O_2$ $[M-H]^-$ 430.9510, found 430.9513.

2,2,2-Trifluoro-1-(2-(4-iodophenyl)benzo[*d*]oxazol-5-yl)ethan-1-one *O*-tosyl oxime 16

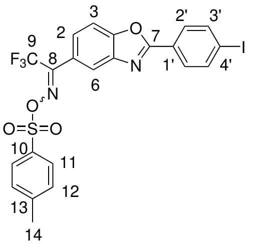

Tosyl oxime **16** was obtained according to General Procedure D, using oxime **15** (1.80 g, 4.17 mmol), *p*-toluenesulfonyl chloride (1.59 g, 8.33 mmol), 4-dimethylaminopyridine (50.9 mg, 0.417 mmol) and NEt_3 (1.73 mL, 12.5 mmol) in CH_2Cl_2 (50 mL). The crude product was purified by flash column chromatography (PE : EtOAc, 19:1 → 4:1) to yield tosyl oxime **16** (1.35 g, 2.30 mmol, 55%) as a pale yellow solid in a mixture of isomers (2:1).

$\mathbf{v_{max}}$ (cm^{-1}) 2981 (C-H), 1622 (C=N-O), 1394 (S=O), 1245 (C-F), 1194 (S=O), 1159; **mp** 171-173 °C;

Major isomer: $\mathbf{R_f}$ 0.45 (PE : EtOAc, 17:3); **^{1}HNMR** (500 MHz, $CDCl_3$) δ_H 7.97 (2H, m, $H_{3'}$), 7.92 (2H, m, $H_{2'}$), 7.90 (2H, d, J = 8.1 Hz, H_{11}), 7.81 (1H, d, J = 1.6 Hz, H_6), 7.68 (1H, d, J = 8.5 Hz, H_3), 7.42 (1H, dd, J = 8.5, 1.6 Hz, H_2), 7.40 (2H, d, J = 8.1 Hz, H_{12}), 2.49 (3H, s, H_{14}). **^{13}CNMR** (125 MHz, $CDCl_3$) δ_C 163.9 (C_7), 153.6 (q, $^2J_{C\text{-}F}$ = 33.5 Hz, C_8), 152.3 (C_4), 146.3 (C_{13}), 142.3 (C_5), 138.4 ($C_{3'}$), 131.1 (C_{10}), 129.9 (C_{12}), 129.3 (C_{11}), 129.2 ($C_{2'}$), 125.9 (C_2), 125.8 (C_{10}), 121.1 (C_1), 120.9 (C_6), 119.6 (q, $^1J_{C\text{-}F}$ = 281.3 Hz, C_9), 111.4 (C_3), 99.4 ($C_{4'}$), 21.8 (C_{14}); **^{19}FNMR** (376 MHz, $CDCl_3$) δ_F −66.7 (s).

Minor isomer: $\mathbf{R_f}$ 0.39 (PE : EtOAc, 17:3); **^{1}HNMR** (500 MHz, $CDCl_3$) δ_H 7.96 (2H, m, $H_{3'}$), 7.91 (2H, m, $H_{2'}$), 7.89 (2H, d, J = 8.1 Hz, H_{11}), 7.85 (1H, d, J = 1.6 Hz, H_6), 7.63 (1H, d, J = 8.5 Hz, H_3), 7.48 (1H, dd, J = 8.5, 1.6 Hz, H_2), 7.39 (2H, d, J = 8.1 Hz, H_{12}), 2.48 (3H, s, H_{14}). **^{13}CNMR** (125 MHz, $CDCl_3$) δ_C 163.9 (C_7), 153.6 (q, $^2J_{C\text{-}F}$ = 33.5 Hz, C_8), 152.7 (C_4), 146.1 (C_{13}), 142.3 (C_5), 138.4 ($C_{3'}$), 131.4 (C_{10}), 123.0 (C_{12}), 129.3 (C_{11}), 129.1 ($C_{2'}$), 125.9 (C_2), 125.8 (C_{10}), 121.2 (C_1), 120.9 (C_6), 119.6 (q, $^1J_{C\text{-}F}$ = 281.3 Hz, C_9), 111.1 (C_3), 99.5 ($C_{4'}$), 21.8 (C_{14}); **^{19}FNMR** (376 MHz, $CDCl_3$) δ_F −66.7 (s);

HRMS (ES$^+$) Calc. for $C_{22}H_{15}F_3IN_2O_4S$ $[M+H]^+$ 586.9744, found 586.9741.

2-(4-Iodophenyl)-5-(3-(trifluoromethyl)diaziridin-3-yl)benzo[*d*]oxazole 17

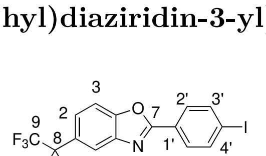

Diaziridine **17** was obtained according to General Procedure E, using tosyl oxime **16** (1.82 g, 3.11 mmol) and NH_3 (5 mL) in CH_2Cl_2 (25 mL). The crude product was purified by flash column chromatography (PE : EtOAc, 4:1) to yield diaziridine **17** (1.05 g, 2.43 mmol, 78%) as a pale yellow solid.

$\mathbf{R_f}$ 0.39 (PE : EtOAc, 4:1); $\mathbf{v_{max}}$ (cm^{-1}) 1593 (N-H), 1479, 1397, 1216 (C-F), 1150; **mp** 152-154 °C; **^{1}HNMR** (500 MHz, $CDCl_3$) δ_H 8.05 (1H, d, J = 1.5 Hz, H_6), 7.97 (2H, m, $H_{3'}$), 7.90 (2H, m, $H_{2'}$), 7.66 (1H, dd, J = 8.5, 1.5 Hz, H_2), 7.62 (1H, d, J = 8.5 Hz, H_3), 2.89 (1H, d, J = 8.9 Hz, -NH), 2.32 (1H, d, J = 8.9 Hz, -NH); **^{13}CNMR** (125 MHz, $CDCl_3$) δ_C 164.0 (C_7), 152.0 (C_4), 142.7 (C_5), 138.7 ($C_{2'}$), 129.5 ($C_{3'}$), 129.0 (C_1), 126.5 ($C_{1'}$), 125.8 (C_2), 123.5 (q, $^1J_{C\text{-}F}$ = 277.6 Hz, C_9), 120.9 (C_6), 111.4 (C_3), 99.5 ($C_{4'}$), 58.2 (q, $^2J_{C\text{-}F}$ = 36.2 Hz, C_8); **^{19}FNMR** (376 MHz, $CDCl_3$) δ_F −75.5 (s); **HRMS** (ES^+) Calc. for $C_{15}H_{10}F_3IN_3O$ $[M+H]^+$ 431.9815, found 431.9815.

2-(4-Iodophenyl)-5-(3-(trifluoromethyl)-3*H*-diazirin-3-yl)benzo[*d*]oxazole 18

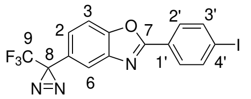

Diazirine **18** was obtained according to General Procedure F, using diaziridine **17** (580 mg, 1.35 mmol), iodine (409 mg, 1.61 mmol) and NEt_3 (0.56 mL, 4.04 mmol) in CH_2Cl_2 (25 mL). The crude product was purified by flash column chromatography (PE : EtOAc, 19:1 → 9:1) to yield diazirine **18** (564 mg, 1.31 mmol, 98%) as a white solid.

$\mathbf{R_f}$ 0.84 (PE : EtOAc, 9:1); $\mathbf{v_{max}}$ (cm^{-1}) 1478, 1397, 1252 (C-F), 1150, 810; **mp** 117-119 °C; **^{1}HNMR** (500 MHz, $CDCl_3$) δ_H 7.95 (2H, m, $H_{3'}$), 7.89 (2H, m, $H_{2'}$), 7.71 (1H, d, J = 1.8 Hz, H_6), 7.59 (1H, d, J = 8.5 Hz, H_3), 7.20 (1H, dd, J = 8.5, 1.8 Hz, H_2); **^{13}CNMR** (125 MHz, $CDCl_3$) δ_C 164.0 (C_7), 151.5 (C_4), 142.7 (C_5), 138.5 ($C_{2'}$), 129.3 ($C_{3'}$), 126.1 ($C_{1'}$), 125.9 (C_1), 124.0 (C_2), 122.2 (q, $^1J_{C\text{-}F}$ = 274.7 Hz, C_9), 119.3 (C_6), 111.4 (C_3), 99.4 ($C_{4'}$). 28.7 (q, $^2J_{C\text{-}F}$ = 40.7 Hz, C_8); **^{19}FNMR** (376 MHz, CD_3OD) δ_F −67.4 (s); **HRMS** (ES^+) Calc. for $C_{15}H_8F_3IN_3O$ $[M+H]^+$ 429.9659, found 429.9655.

2-(4-Ethynylphenyl)-5-(3-(trifluoromethyl)-3*H*-diazirin-3-yl)benzo[*d*]oxazole 3

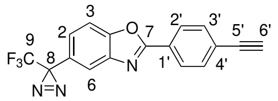

Diazirine **3** was obtained according to General Procedure G, using diazirine **18** (100 mg, 0.233 mmol), bis(triphenylphosphine)palladium(II) dichloride (24.5 mg, 35.0 µmol), copper(I) iodide (4.44 mg, 23.3 µmol), NEt_3 (0.70 mL) and trimethylsilylacetylene (39 µL, 0.280 mmol) in THF (10 mL). The reaction was complete after 1 h. The TMS-protected alkyne intermediate formed was purified by flash column chromatography (PE : EtOAc, 1:0 → 49:1), then deprotected with TBAF (0.70 mL, 1 M in THF) in THF (5 mL). The reaction was complete after 30 min. The crude product was purified by flash column chromatography (PE : EtOAc, 1:0 → 49:1) to yield alkyne **3** (38.2 mg, 0.117 mmol, 50%) as a white solid.

$\mathbf{R_f}$ 0.69 (PE : EtOAc, 49:1); $\mathbf{v_{max}}$ (cm^{-1}) 3311 (sharp, alkyne C-H), 1262 (C-F), 1149, 810, 740; **mp** 125-127 °C; **^{1}HNMR** (500 MHz, $CDCl_3$) δ_H 8.20 (2H, m, $H_{2'}$), 7.72 (1H, d, J = 1.8 Hz, H_6), 7.64 (2H, m, $H_{3'}$), 7.60 (1H, d, J = 8.6 Hz, H_3), 7.20 (1H, dd, J = 8.6, 1.8 Hz, H_2), 3.26 (1H, s, $H_{6'}$); **^{13}CNMR** (125 MHz, $CDCl_3$) δ_C 163.9 (C_7), 151.5 (C_4), 142.8 (C_5), 132.9 ($C_{3'}$), 127.8 ($C_{2'}$), 126.7 ($C_{4'}$), 126.0 ($C_{1'}$), 125.9 (C_1), 124.0 (C_2), 122.3 (q, $^1J_{C\text{-}F}$ = 274.7 Hz, C_9), 119.4 (C_6), 111.4 (C_3), 82.9 ($C_{5'}$), 80.4 ($C_{6'}$), 28.8 (q, $^2J_{C\text{-}F}$ = 40.6 Hz, C_8); **^{19}FNMR** (376 MHz, CD_3OD) δ_F −67.4 (s); **HRMS** (ES^+) Calc. for $C_{17}H_9F_3N_3O$ $[M+H]^+$ 328.0692, found 328.0691.

2-(5-Bromonaphthalen-1-yl)-*N*-methoxy-*N*-methylbenzo[*d*]oxazole-5-carboxamide 19

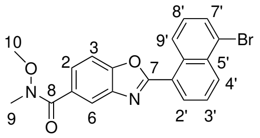

Carboxamide **19** was obtained by a procedure based on General Procedure B except with the addition of 1-hydroxybenzotriazole in the amide coupling. 5-Bromonaphthoic acid (502 mg, 2.00 mmol), thionyl chloride (0.44 mL, 6.00 mmol) and DMF (0.10 mL) were used in CH_2Cl_2 (20 mL). The acid chloride intermediate formed was reacted with 3-amino-4-hydroxybenzoic acid (277 mg, 1.80 mmol, 0.9 eq.) in 1,4-dioxane (5 mL). Amide coupling was performed on the crude product using EDC hydrochloride (575 mg, 3.00 mmol, 1.5 eq.), *N,O*-dimethylhydroxylamine hydrochloride (293 mg, 3.00 mmol, 1.5 eq.), 1-hydroxybenzotriazole (135 mg, 1.00 mmol, 0.5 eq.) and NEt_3 (0.42 mL, 3.00 mmol, 1.5 eq.) in DMF (25 mL). The crude product was purified by flash column chromatography (PE : EtOAc, 1:1) to yield carboxamide **20** (459 mg, 1.12 mmol, 62%) as a white solid.

$\mathbf{R_f}$ 0.54 (PE : EtOAc, 1:1); $\mathbf{v_{max}}$ (cm^{-1}) 2930 (C-H), 1679 ((C=O)NH), 1460, 1251; **mp** 137-138 °C; $^{\mathbf{1}}$**HNMR** (500 MHz, $CDCl_3$) δ_H 9.48 (1H, ddd, J = 8.7, 1.0, 1.0 Hz, $H_{9'}$), 8.48 (1H, ddd, J = 8.6, 1.1, 1.1 Hz, $H_{4'}$), 8.43 (1H, dd, J = 7.3, 1.1 Hz, $H_{2'}$), 8.24 (1H, d, J = 1.6 Hz, H_6), 7.87 (1H, dd, J = 7.4, 1.1 Hz, $H_{7'}$), 7.80 (1H, dd, J = 8.4, 1.6 Hz, H_2), 7.67 (1H, dd, J = 8.5, 7.3 Hz, $H_{3'}$), 7.64 (1H, d, J = 8.4 Hz, H_3), 7.50 (1H, dd, J = 8.7, 7.4 Hz, $H_{8'}$), 3.59 (3H, s, H_{10}), 3.42 (3H, s, H_9); $^{\mathbf{13}}$**CNMR** (125 MHz, $CDCl_3$) δ_C 169.2 (C_8), 163.2 (C_7), 151.4 (C_4), 141.8 (C_5), 132.4 ($C_{5'}$), 132.0 ($C_{10'}$), 131.6 ($C_{4'}$), 130.9 ($C_{7'}$), 130.7 (C_1), 130.2 ($C_{2'}$), 128.2 ($C_{8'}$), 126.5 (C_2), 126.3 ($C_{3'}$), 126.2 ($C_{9'}$), 123.6 ($C_{1'}$ or $C_{6'}$), 123.5 ($C_{1'}$ or $C_{6'}$), 120.8 (C_6), 110.2 (C_3), 61.1 (C_{10}), 33.9 (C_9); **HRMS** (ES$^+$) Calc. for $C_{20}H_{16}{}^{79}BrN_2O_3$ $[M+H]^+$ 411.0339, found 411.0339.

1-(2-(5-Bromonaphthalen-1-yl)benzo[*d*]oxazol-5-yl)-2,2,2-trifluoroethan-1-one oxime 21

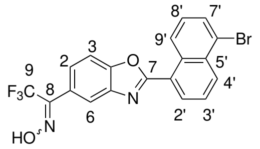

Oxime **21** was obtained according to General Procedure C, using carboxamide **22** (1.18 g, 2.87 mmol), trifluoromethyltrimethylsilane (0.85 mL, 5.74 mmol) and caesium fluoride (87.2 mg, 0.574 mmol) in toluene (40 mL). The reaction was stirred for three days, with further trifluoromethyltrimethylsilane (0.85 mL, 5.74 mmol) and caesium fluoride (87.2 mg, 0.574 mmol) added every 24 h. TBAF (3.44 mL, 1 M in THF) was used for desilylation of the intermediate formed. The crude trifluoromethylketone product was dissolved in pyridine (5 mL) and ethanol (25 mL) and refluxed with hydroxylammonium chloride (598 mg, 8.61 mmol). The crude product was purified by flash column chromatography (PE : EtOAc, 9:1) to yield oxime **21** (699 mg, 1.61 mmol, 56%) as a yellow solid.

$\mathbf{R_f}$ 0.31 (PE : EtOAc, 9:1); $\mathbf{v_{max}}$ (cm^{-1}) 3223 (-OH), 2981 (C-H), 1696 (C=N-OH), 1253 (C-F), 1154; **mp** 182-183 °C; $^{\mathbf{1}}$**HNMR** (500 MHz, $(CD_3)_2CO$) δ_H 11.96 (1H, *br* s, -OH), 9.63 (1H, ddd, J = 8.7, 1.1, 1.1 Hz, $H_{9'}$), 8.61 (1H, dd, J = 7.4, 1.1 Hz, $H_{2'}$), 8.57 (1H, ddd, J = 8.6, 1.1, 1.1 Hz, $H_{4'}$), 8.06 (1H, s, H_6), 8.05 (1H, *obs* m, $H_{7'}$), 7.81 (1H, d, J = 8.4 Hz, H_3), 7.76 (1H, dd, J = 8.6, 7.4 Hz, $H_{3'}$), 7.67 (1H, dd, J = 8.7, 7.6 Hz, $H_{8'}$), 7.65 (1H, dd, J = 8.4, 1.6 Hz, H_2); $^{\mathbf{13}}$**CNMR** (125 MHz, $CDCl_3$) δ_C 163.8 (C_7), 151.9 (C_8), 151.7 (C_4), 143.1 (C_5), 133.1 (C_1), 132.8 ($C_{5'}$), 132.2 ($C_{10'}$), 132.0 ($C_{4'}$), 131.5 ($C_{7'}$), 131.4 ($C_{2'}$), 129.3 ($C_{8'}$), 127.8 ($C_{3'}$), 127.5 (C_2), 127.4 ($C_{9'}$), 124.6 ($C_{1'}$), 123.7 ($C_{6'}$), 122.2 (q, $^1J_{C\text{-}F}$ = 273.1 Hz, C_9), 121.9 (C_6), 111.8 (C_3); $^{\mathbf{19}}$**FNMR** (376 MHz, $CDCl_3$) δ_F −67.6 (s); **HRMS** (ES$^+$) Calc. for $C_{19}H_{11}F_3{}^{79}BrN_2O_2$ $[M+H]^+$ 434.9951, found 434.9951.

1-(2-(5-Bromonaphthalen-1-yl)benzo[*d*]oxazol-5-yl)-2,2,2-trifluoroethan-1-one *O*-tosyl oxime 23

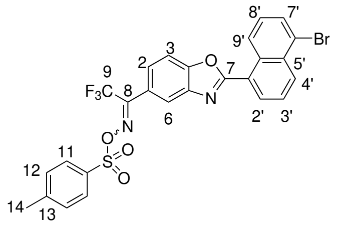

Tosyl oxime **23** was obtained according to General Procedure D, using oxime **24** (818 mg, 1.88 mmol), *p*-toluenesulfonyl chloride (717 mg, 3.76 mmol), 4-dimethylaminopyridine (23.0 mg, 0.188 mmol) and NEt_3 (0.78 mL, 5.64 mmol) in CH_2Cl_2 (30 mL). The crude product was purified by flash column chromatography (PE : EtOAc, 19:1) to yield tosyl oxime **23** (820 mg, 1.39 mmol, 74%) as a pale yellow solid in a mixture of isomers (50:50).

$\mathbf{v_{max}}$ (cm^{-1}) 2980 (C-H), 1334 (S=O), 1255 (C-F), 1196 (S=O), 1154; **mp** 148-150 ℃;

Minor isomer: $\mathbf{R_f}$ (0.35, PE : EtOAc, 19:1); $\mathbf{^1HNMR}$ (500 MHz, $CDCl_3$) δ_H 9.48 (1H, ddd, J = 8.8, 1.0, 1.0 Hz, $H_{9'}$), 8.54 (1H, ddd, J = 8.6, 1.1, 1.1 Hz, $H_{4'}$), 8.46 (1H, dd, J = 7.3, 1.2 Hz, $H_{2'}$), 7.94-7.89 (4H, m, H_6, $H_{7'}$ and H_{11}), 7.73 (1H, d, J = 8.4 Hz, H_3), 7.71 (1H, dd, J = 8.6, 7.3 Hz, $H_{3'}$), 7.54 (1H, dd, J = 8.8, 7.4 Hz, $H_{8'}$), 7.47 (1H, dd, J = 8.5, 1.7 Hz, H_2), 7.41 (2H, m, H_{12}), 2.49 (3H, s, H_{14}); $\mathbf{^{13}CNMR}$ (125 MHz, $CDCl_3$) δ_C 163.8 (C_7), 153.7 (q, $^2J_{C\text{-}F}$ = 34.1 Hz, C_8), 151.8 (C_4), 146.2 (C_{14}), 142.6 (C_5), 132.6 ($C_{5'}$), 132.2 ($C_{10'}$), 132.1 ($C_{4'}$), 131.3 ($C_{1'}$), 131.1 ($C_{7'}$), 130.6 ($C_{2'}$), 130.1 (C_{12}), 129.5 (C_{10}), 129.3 (C_{11}), 128.5 ($C_{8'}$), 126.4 ($C_{3'}$), 126.2 (C_2), 126.2 ($C_{9'}$), 123.7 ($C_{6'}$), 123.4 (C_1), 121.3 (C_6), 119.8 (q, $^1J_{C\text{-}F}$ = 277.5 Hz, C_9), 111.2 (C_3), 22.0 (C_{14}); $\mathbf{^{19}FNMR}$ (376 MHz, $CDCl_3$) δ_F −66.6 (s);

Major isomer: $\mathbf{R_f}$ (0.28, PE : EtOAc, 19:1); $\mathbf{^1HNMR}$ (500 MHz, $CDCl_3$) δ_H 9.49 (1H, ddd, J = 8.8, 1.0, 1.0 Hz, $H_{9'}$), 8.54 (1H, ddd, J = 8.6, 1.1, 1.1 Hz, $H_{4'}$), 8.46 (1H, dd, J = 7.3, 1.2 Hz, $H_{2'}$), 7.96-7.91 (4H, m, H_6, $H_{7'}$ and H_{11}), 7.71 (1H, dd, J = 8.6, 7.3 Hz, $H_{3'}$), 7.68 (1H, d, J = 8.4 Hz, H_3), 7.55 (1H, dd, J = 8.8, 7.4 Hz, $H_{8'}$), 7.52 (1H, dd, J = 8.5, 1.7 Hz, H_2), 7.40 (2H, m, H_{12}), 2.47 (3H, s, H_{14}); $\mathbf{^{13}CNMR}$ (125 MHz, $CDCl_3$) δ_C 163.9 (C_7), 153.9 (q, $^2J_{C\text{-}F}$ = 32.9 Hz, C_8), 152.2 (C_4), 146.4 (C_{13}), 142.7 (C_5), 132.6 ($C_{5'}$), 132.2 ($C_{10'}$), 132.1 ($C_{4'}$), 131.3 ($C_{1'}$), 131.1 ($C_{7'}$), 130.6 ($C_{2'}$), 130.1 (C_{12}), 129.5 (C_{10}), 129.3 (C_{11}), 128.5 ($C_{8'}$), 126.4 ($C_{3'}$), 126.3 (C_2), 126.2 ($C_{9'}$), 123.7 ($C_{6'}$), 123.6 (C_1), 121.6 (C_6), 119.8 (q, $^1J_{C\text{-}F}$ = 277.5 Hz, C_9), 111.5 (C_3), 22.0 (C_{14}); $\mathbf{^{19}FNMR}$ (376 MHz, $CDCl_3$) δ_F −66.6 (s);

HRMS (ES^+) Calc. for $C_{26}H_{17}F_3{}^{79}BrN_2O_4S$ $[M+H]^+$ 589.0039, found 589.0035.

2-(5-Bromonaphthalen-1-yl)-5-(3-(trifluoromethyl)diaziridin-3-yl)benzo[*d*]oxazole 25

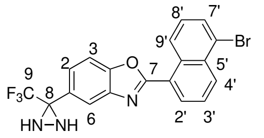

Diaziridine **25** was obtained according to General Procedure E, using tosyl oxime **23** (630 mg, 1.07 mmol) and NH_3 (5 mL) in CH_2Cl_2 (10 mL). The crude product was used without further purification, yielding diaziridine **25** (469 mg, 1.07 mmol, quant.) as a white solid.

$\mathbf{R_f}$ (0.67, PE : EtOAc, 4:1); $\mathbf{v_{max}}$ (cm^{-1}) 2981 (C-H), 1457, 1157, 1036; **mp** 149-150 ℃; $\mathbf{^1HNMR}$ (500 MHz, $CDCl_3$) δ_H 9.51 (1H, ddd, J = 8.5, 1.1, 1.1 Hz, $H_{9'}$), 8.55 (1H, ddd, J = 8.6, 1.1, 1.1 Hz, $H_{4'}$), 8.49 (1H, dd, J = 7.4, 1.1 Hz, $H_{2'}$), 8.17 (1H, s, H_6), 7.92 (1H, dd, J = 7.4, 1.1 Hz, $H_{7'}$), 7.73 (1H, dd, J = 8.6, 7.4 Hz, $H_{3'}$), 7.70 (2H, s, H_2 and H_3), 7.55 (1H, dd, J = 8.5, 7.4 Hz, $H_{8'}$), 2.91 (1H, d, J = 8.9 Hz, -NH), 2.34 (1H, d, J = 8.9 Hz, -NH); $\mathbf{^{13}CNMR}$ (125 MHz, $CDCl_3$) δ_C 163.5 (C_7), 151.0 (C_4), 142.5 (C_5), 132.5 ($C_{5'}$), 132.0 ($C_{10'}$), 131.9 ($C_{4'}$), 130.9 ($C_{7'}$), 130.3 ($C_{2'}$), 129.7 (C_1), 128.3 ($C_{8'}$), 127.2 ($C_{1'}$), 126.3 ($C_{3'}$ or $C_{9'}$), 126.2 ($C_{3'}$ or $C_{9'}$), 125.6 (C_2), 124.5 (q, $^1J_{C\text{-}F}$ = 276.6 Hz, C_9),

123.6 ($C_{6'}$), 120.8 (C_6), 110.9 (C_3), 58.3 (q, $^2J_{C\text{-}F}$ = 36.1 Hz, C_8);**^{19}FNMR** (376 MHz, $CDCl_3$) δ_F −75.5 (s); **HRMS** (ES^+) Calc. for $C_{19}H_{12}{}^{79}BrF_3N_3O$ $[M+H]^+$ 434.0105, found 434.0110.

2-(5-Bromonaphthalen-1-yl)-5-(3-(trifluoromethyl)-3*H*-diazirin-3-yl)benzo[*d*]oxazole 26

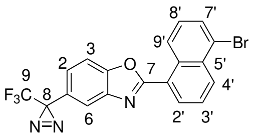

Diazirine **26** was obtained according to General Procedure F, using diaziridine **25** (439 mg, 1.01 mmol), iodine (308 mg, 1.21 mmol) and NEt_3 (0.42 mL, 3.03 mmol) in CH_2Cl_2 (20 mL). The crude product was purified by flash column chromatography (PE : EtOAc, 49:1 → 19:1) to yield diazirine **26** (407 mg, 0.945 mmol, 94%) as a white solid.

R_f (0.53, PE : EtOAc, 49:1); **v_{max}** (cm^{-1}) 2981 (C-H), 1258 (C-F), 1154, 773; **mp** 104-106 °C; **^{1}HNMR** (500 MHz, $CDCl_3$) δ_H 9.50 (1H, ddd, J = 8.7, 1.1, 1.1 Hz, $H_{9'}$), 8.54 (1H, ddd, J = 8.6, 1.1, 1.1 Hz, $H_{4'}$), 8.47 (1H, dd, J = 7.3, 1.1 Hz, $H_{2'}$), 7.92 (1H, dd, J = 7.4, 1.0 Hz, $H_{7'}$), 7.83 (1H, dd, J = 1.8, 0.6 Hz, H_6), 7.72 (1H, dd, J = 8.6, 7.3 Hz, $H_{3'}$), 7.66 (1H, d, J = 8.6, 0.6 Hz, H_3), 7.54 (1H, dd, J = 8.7, 7.4 Hz, $H_{8'}$), 7.24 (1H, dd, J = 8.6, 1.8 Hz, H_2); **^{13}CNMR** (125 MHz, $CDCl_3$) δ_C 163.6 (C_7), 150.7 (C_4), 142.8 (C_5), 132.4 ($C_{5'}$), 132.1 ($C_{10'}$), 132.0 ($C_{4'}$), 131.0 ($C_{7'}$), 130.4 ($C_{2'}$), 128.3 ($C_{8'}$), 126.3 ($C_{3'}$), 126.1 ($C_{9'}$), 125.6 (C_1), 124.0 (C_2), 123.6 ($C_{1'}$ or $C_{6'}$), 123.4 ($C_{1'}$ or $C_{6'}$), 122.3 (q, $^1J_{C\text{-}F}$ = 274.7 Hz, C_9), 119.5 (C_6), 111.2 (C_3), 28.8 (q, $^2J_{C\text{-}F}$ = 40.6 Hz, C_8); **^{19}FNMR** (376 MHz, $CDCl_3$) δ_F −65.4 (s); **HRMS** (ES^+) Calc. for $C_{19}H_{10}{}^{79}BrF_3N_3O$ $[M+H]^+$ 431.9954, found 431.9950.

2-(5-Ethynylnaphthalen-1-yl)-5-(3-(trifluoromethyl)-3*H*-diazirin-3-yl)benzo[*d*]oxazole 4

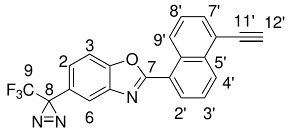

Diazirine **4** was obtained according to General Procedure G, using diazirine **26** (200 mg, 0.465 mmol), bis(triphenylphosphine)palladium(II) dichloride (48.9 mg, 69.7 µmol), copper(I) iodide (8.86 mg, 46.5 µmol), NEt_3 (1.50 mL) and trimethylsilylacetylene (78.2 µL, 0.558 mmol) in THF (10 mL). The reaction was worked up after 6 h, although starting material was still present. The TMS-protected alkyne intermediate formed was purified by flash column chromatography (PE : EtOAc, 1:0 → 99:1), then deprotected with TBAF (0.93 mL, 1 M in THF) in THF (5 mL). The reaction was complete after 30 min. The crude product was purified by flash column chromatography (pentane : toluene, 19:1) to yield alkyne **4** (58.9 mg, 0.156 mmol, 34%) as an off-white solid.

R_f (0.16, pentane : toluene, 19:1); **v_{max}** (cm^{-1}) 2981 (C-H), 1256 (C-F), 1154, 773; **mp** 87-89 °C; **^{1}HNMR** (500 MHz, $CDCl_3$) δ_H 9.52 (1H, ddd, J = 8.6, 1.0, 1.0 Hz, $H_{9'}$), 8.64 (1H, ddd, J = 8.4, 1.1, 1.1 Hz, $H_{4'}$), 8.46 (1H, dd, J = 7.3, 1.3 Hz, $H_{2'}$), 7.85 (1H, dd, J = 7.1, 1.2 Hz, $H_{7'}$), 7.82 (1H, d, J = 1.7 Hz, H_6), 7.70 (1H, dd, J = 8.4, 7.3 Hz, $H_{3'}$), 7.65 (1H, dd, J = 8.7, 7.1 Hz, $H_{8'}$), 7.64 (1H, d, J = 8.7 Hz, H_3), 7.24 (1H, dd, J = 8.4, 1.9 Hz, H_2), 3.53 (1H, s, $H_{12'}$); **^{13}CNMR** (125 MHz, $CDCl_3$) δ_C 163.9 (C_7), 150.7 (C_4), 142.8 (C_5), 134.0 ($C_{5'}$), 131.9 ($C_{7'}$), 130.9 ($C_{4'}$), 130.5 ($C_{2'}$), 130.2 ($C_{10'}$), 127.4 ($C_{9'}$), 127.3 ($C_{8'}$), 125.9 ($C_{3'}$), 125.6 (C_1), 123.9 (C_2), 123.2 ($C_{1'}$), 122.3 (q, $^1J_{C\text{-}F}$ = 274.7 Hz, C_9), 120.6 ($C_{6'}$), 119.4 (C_6), 111.1 (C_3), 82.7 ($C_{12'}$), 81.5 ($C_{11'}$), 28.8 (q, $^2J_{C\text{-}F}$ = 40.6 Hz, C_8); **^{19}FNMR** (376 MHz, CD_3OD) δ_F −65.4 (s); **HRMS** (CI^+) Calc. for $C_{21}H_{10}F_3N_3O$ $[M^+]$ 377.0770 found 377.0778.

5-bromo-2-(4-fluorophenyl)benzo[*d*]oxazole 27

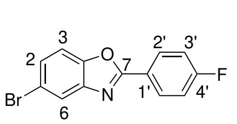

4-fluorobenzoyl chloride (18.6 g, 0.12 mol) was added to a suspension of 2-amino-4-bromophenol (20.0 g, 0.11 mol) in xylenes (100 mL) and refluxed for 1 h. Then methanesulfonic acid (2.0 g, 0.02 mol) was added and the mixture was refluxed for a further 1 h. The reaction was cooled to RT, diluted with EtOAc (500 mL) and washed with water, sat. aq. $NaHCO_3$ and brine. The combined organic layers were dried over Na_2SO_4, filtered and concentrated *in vacuo*. The residue was recrystallized from petroleum ether/EtOAc (2:1) and filtered to afford the title compound (20.0 g, 90%).

mp 149-151 °C; **^{1}H NMR** (500 MHz, $CDCl_3$) δ 8.27 - 8.21 (2H, m, $H_{2'}$), 7.89 (1H, d, J = 1.6 Hz, H_6), 7.47 (1H, dd, J = 8.6, 1.7 Hz, H_2), 7.45 (1H, d, J = 8.5 Hz, H_3), 7.25 - 7.20 (2H, m, $H_{3'}$); **^{13}C NMR** (125 MHz, $CDCl_3$) δ 165.20 (d, $^1J_{C\text{-}F}$ = 253.6 Hz, $C_{4'}$), 163.4 (C_7), 149.9 (C_4), 143.9 (C_5), 130.2 (d, $^3J_{C\text{-}F}$ = 9.0 Hz, $C_{2'}$), 128.3 (C_2), 123.2 ($C_{1'}$), 123.1 (C_6), 117.5 (C_1), 116.5 (d, $^2J_{C\text{-}F}$ = 22.2 Hz, $C_{3'}$), 111.9 (C_3); **^{19}F NMR** (376 MHz, $CDCl_3$) xx; **HRMS** (ES^+) Calc. for $C_{13}H_8{}^{79}BrFNO$ $[M + H]^+$ 291.9768, found 291.9769.

methyl ethyl(2-(4-fluorophenyl)benzo[*d*]oxazol-5-yl)phosphinate (SMT021256) 8

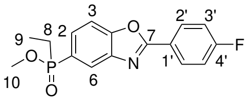

To a degassed solution of 5-bromo-2-(4-fluorophenyl)benzo[*d*]oxazole (20.0 g, 0.07 mol), ethylphosphinic acid (14.8 g, 0.14 mol) and DIPEA (25 mL, 0.14 mol) in DME (100 mL) and toluene (300 mL) $Pd(OAc)_2$ (0.92 g, 0.004 mol) and XantPhos (2.27 g, 0.004 mol) were added under N_2 and the reaction was stirred at 90°C for 2 h. The solvents were removed *in vacuo* and the residue partitioned between EtOAc and NaOH (1M). The aqueous layer was extracted into EtOAc, acidified with 1M HCl (pH 3) and extracted with EtOAc. The combined organic layers were washed with brine, dried over Na_2SO_4, and the desired product was obtained by column chromatography (5% MeOH/DCM) as an off-white solid (18.9 g, 86%).

mp 90-2 °C; **^{1}H NMR** (500 MHz, $(CD_3)_2SO$) δ 8.32 - 8.25 (2H, m, $H_{2'}$), 8.16 - 8.10 (1H, m, H_6), 7.97 (1H, dd, J = 8.3, 2.3 Hz, H_3), 7.79 (1H, ddd, J = 10.8, 8.3, 1.4 Hz, H_2), 7.53 - 7.44 (2H, m, $H_{3'}$), 3.53 (3H, d, J = 10.9 Hz, H_{10}), 2.09 - 1.91 (2H, m, H_8), 0.97 (3H, dt, J = 18.9, 7.6 Hz, H_9); **^{13}C NMR** (125 MHz, $(CD_3)_2SO$) δ 164.5 (d, $^1J_{C\text{-}F}$ = 250.8 Hz, $C_{4'}$), 162.7 (C_7), 152.7 (d, $^4J_{C\text{-}P}$ = 3.4 Hz, C_4), 141.8 (d, $^3J_{C\text{-}P}$ = 16.4 Hz, C_5), 130.3 (d, $^3J_{C\text{-}F}$ = 9.4 Hz, $C_{2'}$), 128.9 (d, $^2J_{C\text{-}P}$ = 11.3 Hz, C_2), 126.9 (d, $^1J_{C\text{-}P}$ = 121.1 Hz, C_1), 123.4 (d, $^4J_{C\text{-}F}$ = 11.1 Hz, $C_{1'}$), 122.7 (d, $^2J_{C\text{-}P}$ = 3.4 Hz, C_6), 116.7 (d, $^2J_{C\text{-}F}$ = 22.1 Hz, $C_{3'}$), 111.7 (d, $^3J_{C\text{-}P}$ = 13.5 Hz, C_3), 50.8 (d, $^2J_{C\text{-}P}$ = 6.6 Hz, C_{10}), 21.2 (d, $^1J_{C\text{-}P}$ = 101.3 Hz, C_8), 5.8 (d, $^2J_{C\text{-}P}$ = 4.7 Hz, C_9); **^{31}P NMR {H}** (162 MHz, $(CD_3)_2SO$) δ 46.8 (s); **^{19}F NMR** (376 MHz, $(CD_3)_2SO$) δ -106.8 (m); **LC-MS** (254 nm, 100%) R_T 2.34 min, MS (ES^+) Calc. for $C_{16}H_{15}FNO_3P$ $[M + H]^+$ 320.1, found 320.1; **HRMS** (ES^+) Calc. for $C_{16}H_{15}FNO_3P$ $[M + H]^+$ 320.0846, found 320.0843.

The two enantiomers were isolated by chiral separation. SMT021256 was dissolved in MeOH (100 mg/mL) and then purified by supercritical flash chromatography with a Lux C4 column (21.2 mm x 250 mm, 5 μm) and isocratic $MeOH/CO_2$ (35:65). From 10076.2 mg racemic mixture, 3647.3 mg of the first eluting enantiomer (R_T 1.85 min, 49.8%) were collected and assigned the name SMT022331 and 4962.1 mg of the second enantiomer (R_T 3.15 min, 50.2%) were collected and assigned the name SMT022332.

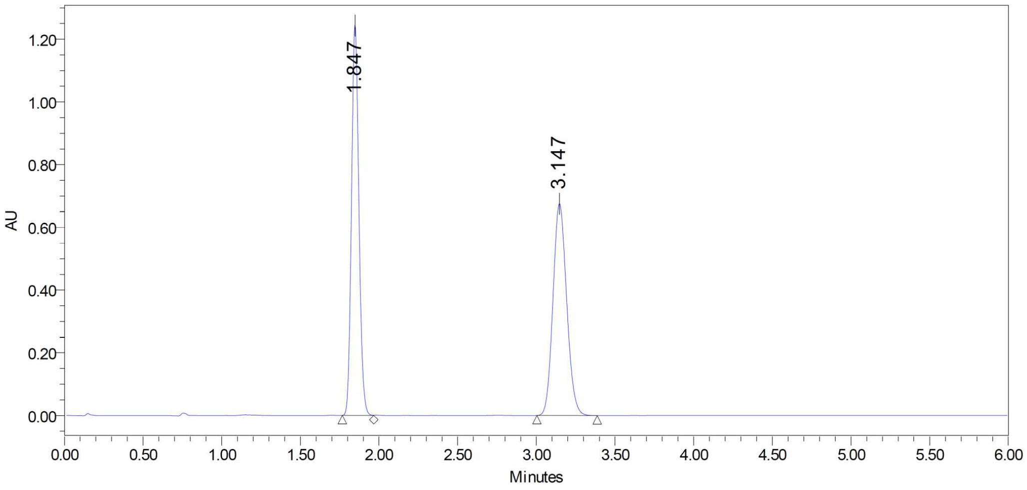

methyl (−)-ethyl(2-(4-fluorophenyl)benzo[*d*]oxazol-5-yl)phosphinate (SMT022331) 28

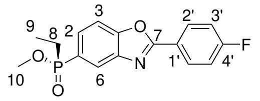

mp 88 °C. **^{1}H NMR** (500 MHz, $(CD_3)_2SO$) δ 8.32 - 8.25 (2H, m, $H_{2'}$), 8.16 - 8.10 (1H, m, H_6), 7.97 (1H, dd, J = 8.3, 2.3 Hz, H_3), 7.79 (1H, ddd, J = 10.8, 8.3, 1.4 Hz, H_2), 7.53 - 7.44 (2H, m, $H_{3'}$), 3.53 (3H, d, J = 10.9 Hz, H_{10}), 2.09 - 1.91 (2H, m, H_8), 0.97 (3H, dt, J = 18.9, 7.6 Hz, H_9); **^{13}C NMR** (125 MHz, $(CD_3)_2SO$) δ 164.5 (d, $^{1}J_{\text{C-F}}$ = 250.8 Hz, $C_{4'}$), 162.7 (C_7), 152.7 (d, $^{4}J_{\text{C-P}}$ = 3.4 Hz, C_4), 141.8 (d, $^{3}J_{\text{C-P}}$ = 17.2 Hz, C_5), 130.3 (d, $^{3}J_{\text{C-F}}$ = 9.7 Hz, $C_{2'}$), 128.9 (d, $^{2}J_{\text{C-P}}$ = 11.2 Hz, C_2), 126.9 (d, $^{1}J_{\text{C-P}}$ = 120.9 Hz, C_1), 123.4 (d, $^{4}J_{\text{C-F}}$ = 11.0 Hz, $C_{1'}$), 122.7 (d, $^{2}J_{\text{C-P}}$ = 3.1 Hz, C_6), 116.7 (d, $^{2}J_{\text{C-F}}$ = 22.1 Hz, $C_{3'}$), 111.7 (d, $^{3}J_{\text{C-P}}$ = 13.7 Hz, C_3), 50.8 (d, $^{2}J_{\text{C-P}}$ = 6.5 Hz, C_{10}), 21.2 (d, $^{1}J_{\text{C-P}}$ = 101.7 Hz, C_8), 5.8 (d, $^{2}J_{\text{C-P}}$ = 4.6 Hz, C_9); **^{31}P NMR {H}** (162 MHz, $(CD_3)_2SO$) δ 46.8. **^{19}F NMR** (376 MHz, $(CD_3)_2SO$) δ -106.7 (m); **HRMS** (ES^+) Calc. for $C_{16}H_{15}FNO_3P$ $[M + H]^+$ 320.0846, found 320.0845; $[\alpha_D^{25}] - 40.7[c1.00, \text{THF}]$.

methyl (+)-ethyl(2-(4-fluorophenyl)benzo[*d*]oxazol-5-yl)phosphinate (SMT022332) 7

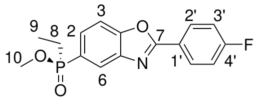

mp 86-7 °C; **^{1}H NMR** (500 MHz, $(CD_3)_2SO$) δ 8.31 - 8.25 (2H, m, $H_{2'}$), 8.13 (1H, dd, J = 11.6, 1.4 Hz, H_6), 7.97 (1H, dd, J = 8.3, 2.3 Hz, H_3), 7.79 (1H, ddd, J = 10.8, 8.3, 1.4 Hz, H_2), 7.53 - 7.44 (2H, m, $H_{3'}$), 3.53 (3H, d, J = 10.9 Hz, H_{10}), 2.09 - 1.91 (2H, m, H_8), 0.97 (3H, dt, J = 18.9, 7.6 Hz, H_9); **^{13}C NMR** (125 MHz, $(CD_3)_2SO$) δ 164.5 (d, $^{1}J_{\text{C-F}}$ = 251.5 Hz, $C_{4'}$), 162.7 (C_7), 152.7 (d, $^{4}J_{\text{C-P}}$ = 3.4 Hz, C_4), 141.8 (d, $^{3}J_{\text{C-P}}$ = 17.0 Hz, C_5), 130.3 (d, $^{3}J_{\text{C-F}}$ = 9.3 Hz, $C_{2'}$), 128.9 (d, $^{2}J_{\text{C-P}}$ = 11.0 Hz, C_2), 126.9 (d, $^{1}J_{\text{C-P}}$ = 121.6 Hz, C_1), 123.4 (d, $^{4}J_{\text{C-F}}$ = 11.0 Hz, $C_{1'}$), 122.7 (d, $^{2}J_{\text{C-P}}$ = 3.3 Hz, C_6), 116.7 (d, $^{2}J_{\text{C-F}}$ = 22.0 Hz, $C_{3'}$), 111.7 (d, $^{3}J_{\text{C-P}}$ = 13.6 Hz, C_3), 50.8 (d, $^{2}J_{\text{C-P}}$ = 6.4 Hz, C_{10}), 21.2 (d, $^{1}J_{\text{C-P}}$ = 101.6 Hz, C_8), 5.8 (d, $^{2}J_{\text{C-P}}$ = 4.6 Hz, C_9);**^{31}P NMR {H}** (162 MHz, $(CD_3)_2SO$) δ 46.8; **^{19}F NMR** (376 MHz, $(CD_3)_2SO$) δ -106.8 (m); **HRMS** (ES^+) Calc. for $C_{16}H_{15}FNO_3P$ $[M + H]^+$ 320.0846, found 320.0846; $[\alpha_D^{25}] + 40.7[c1.00, \text{THF}]$.

Chemical and chiral purity of SMT022331 and SMT022332

Chemical purity analysis conditions

Column Details	XSelect CSH C18 (50 x 2.1 mm, 1.7 µm)		
Column Temperature	40 ℃		
Flow Rate	0.6 mL/minute		
Detector Wavelength	240 nm		
Injection Volume	1.0 µL		
Mobile Phase A	Water (0.1% v/v TFA)		
Mobile Phase B	MeCN (0.1% v/v TFA)		
Gradient Profile	Time (min)	%A	%B
	0	97	3
	4	3	97
	4.02	0	100
	4.5	0	100
	4.52	97	3
	6	97	3

Chiral purity analysis conditions

Column Details	Lux C4 (4.6 mm x 250 mm, 5 µm)
Column Temperature	40 ℃
Flow Rate	4 mL/minute
Detector Wavelength	210-240 nm
Injection Volume	1.0 µL
Isocratic conditions	36:65 MeOH/CO_2

methyl (−)-ethyl(2-(4-fluorophenyl)benzo[*d*]oxazol-5-yl)phosphinate (SMT022331)
Chemical purity (100%)

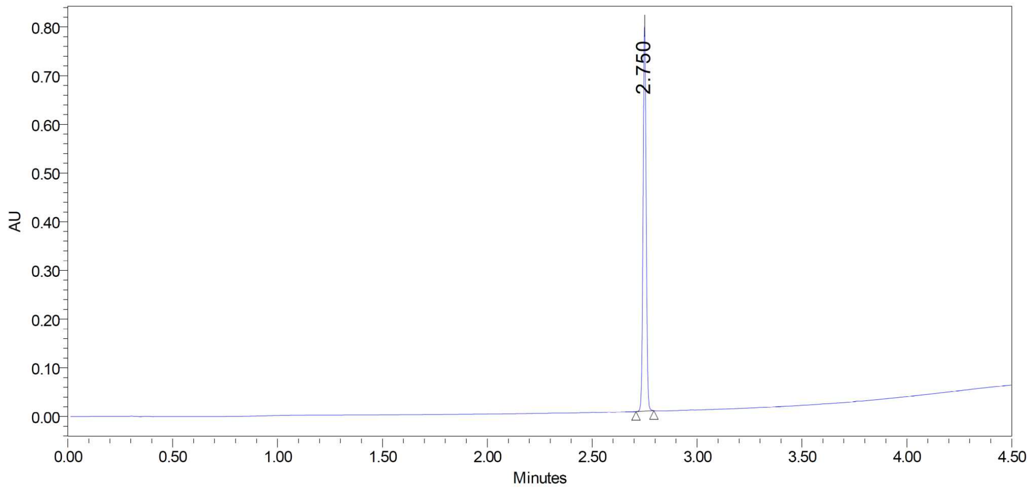

Chiral purity (100%)

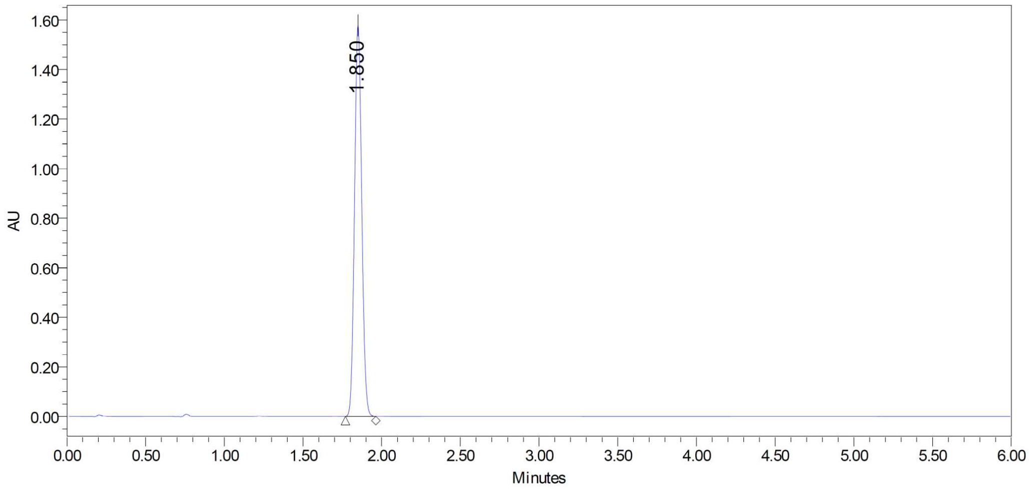

methyl (+)-ethyl(2-(4-fluorophenyl)benzo[*d*]oxazol-5-yl)phosphinate (SMT022332)
Chemical purity (99.7%)

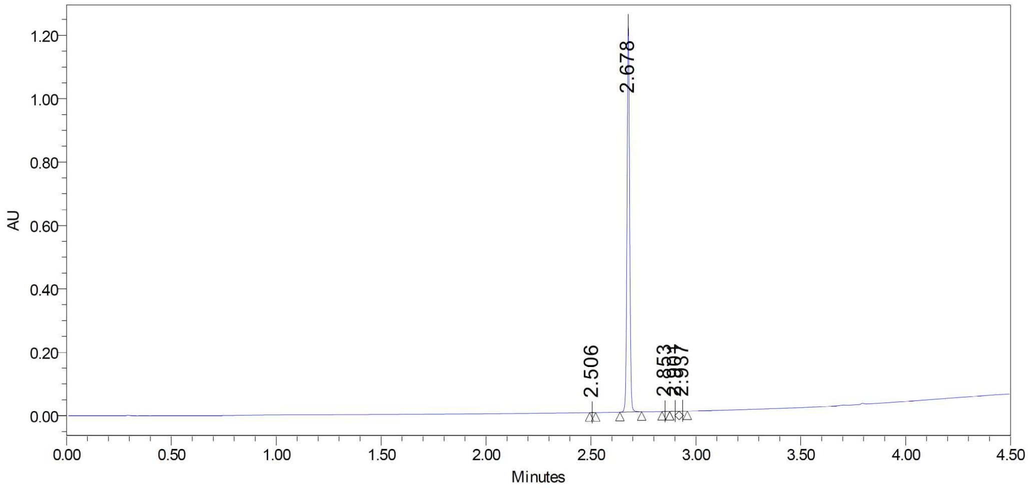

Chiral purity (99.9%)

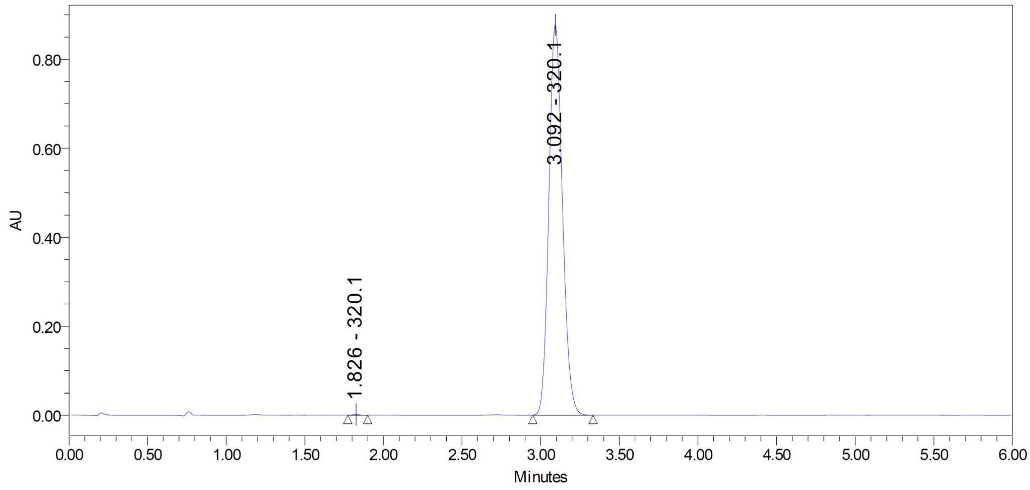

Spectra:

2-(4-ethynylphenyl)-5-(3-(trifluoromethyl)-3H-diazirin-3-yl)benzo[d]oxazole 3

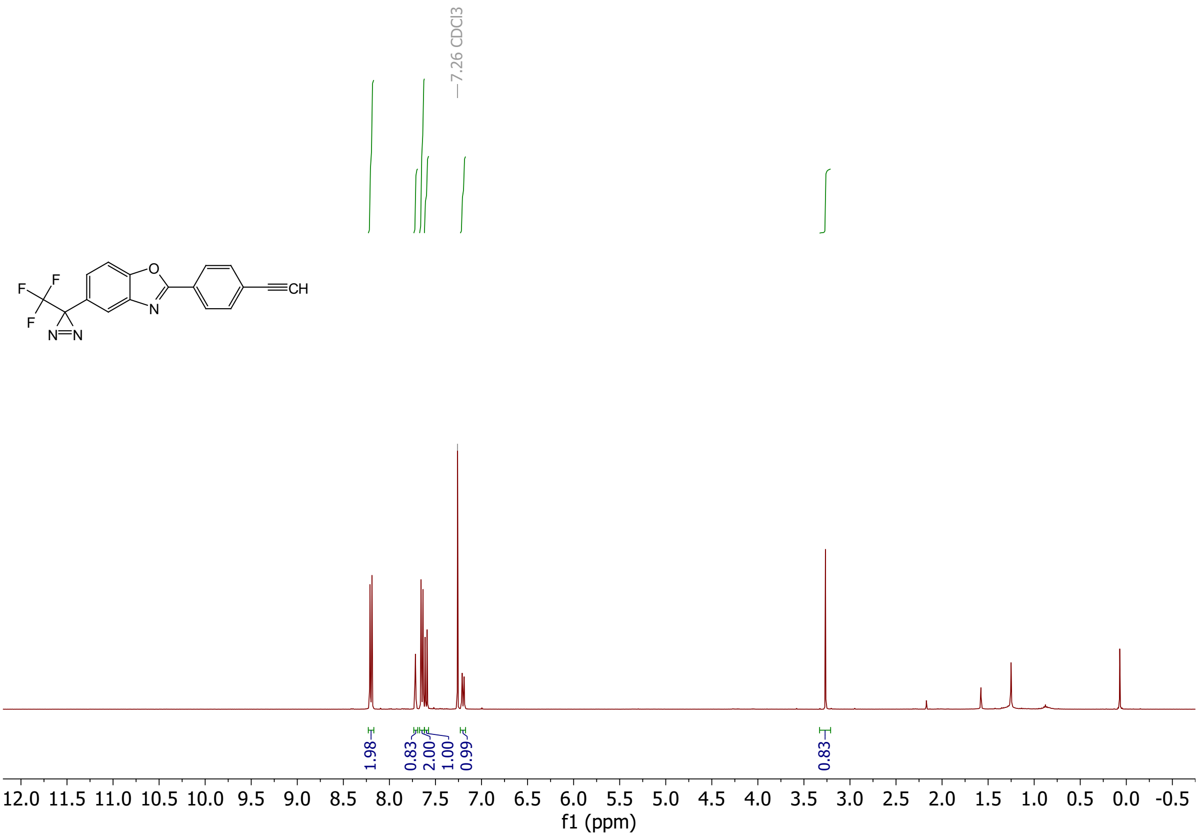

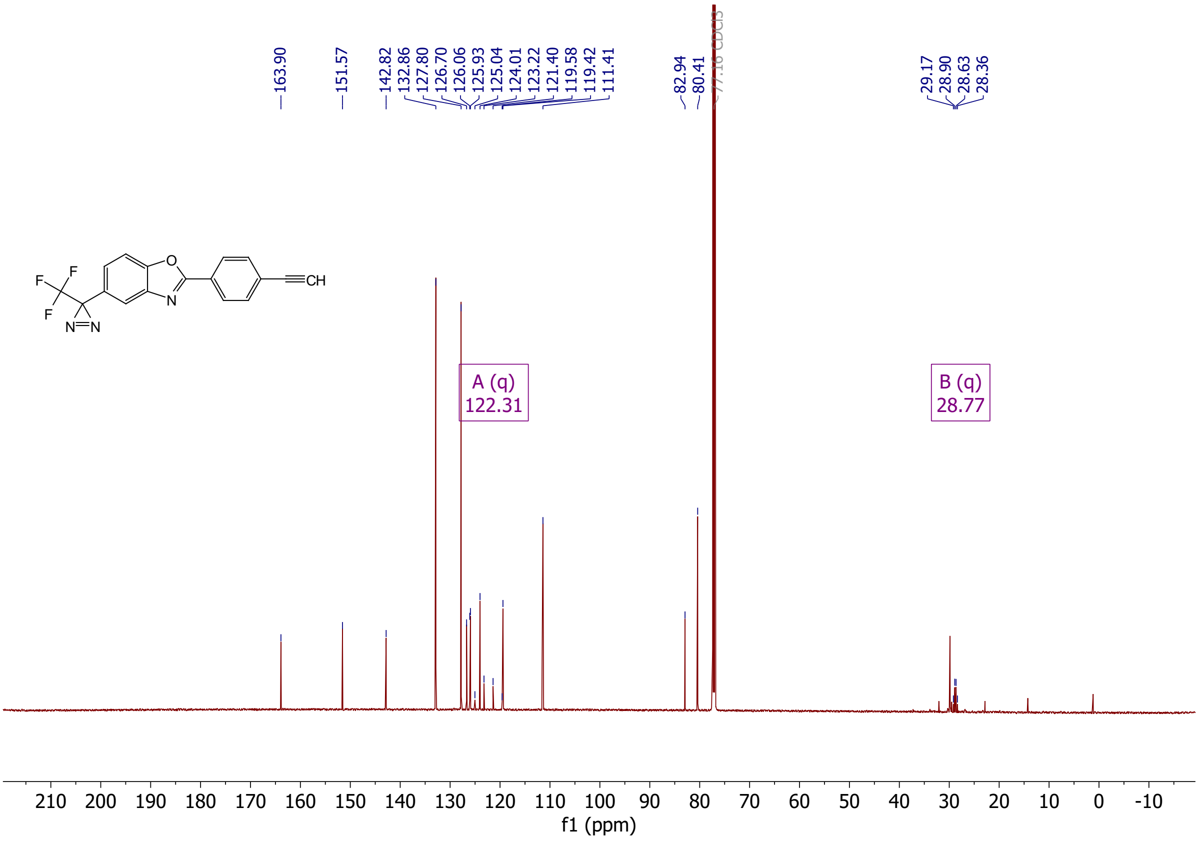

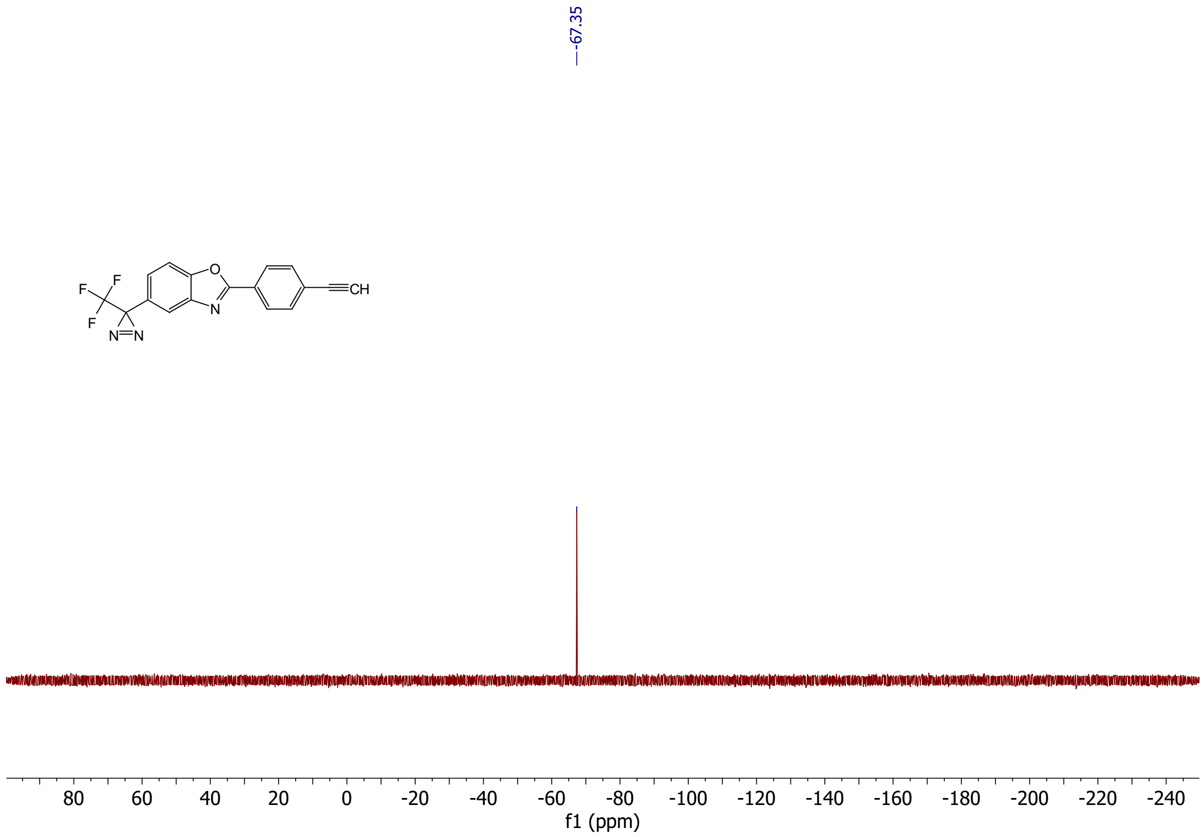

2-(3-ethynylphenyl)-5-(3-(trifluoromethyl)-3H-diazirin-3-yl)benzo[d]oxazole 2

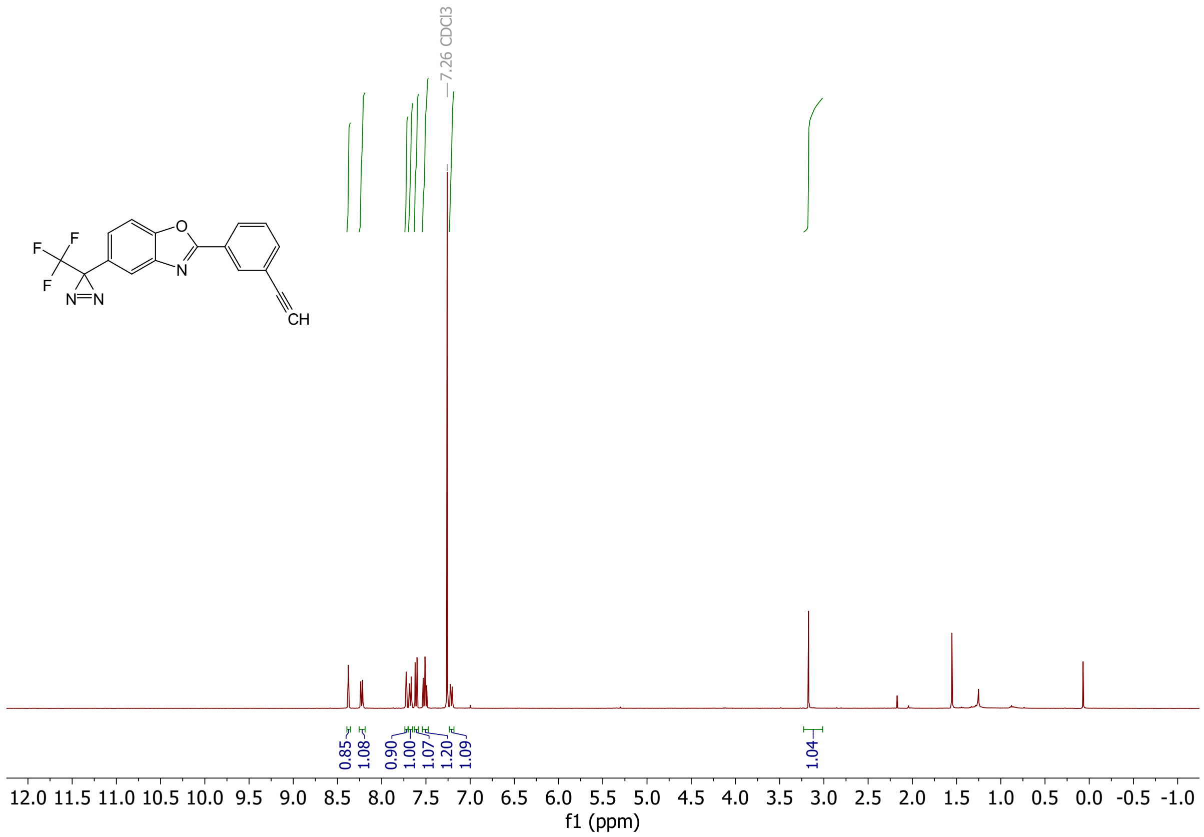

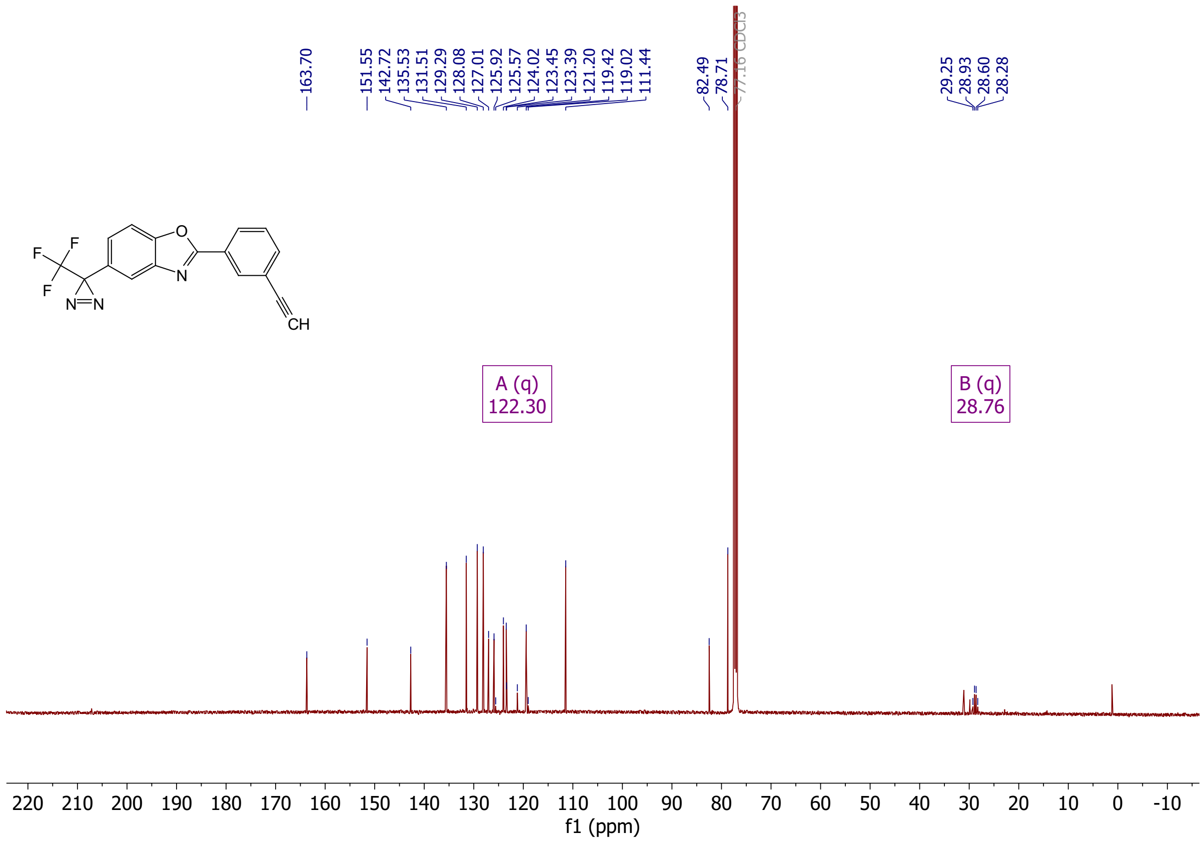
163.70
151.55
142.72
135.53
131.51
129.29
128.08
127.01
125.92
125.57
124.02
123.45
123.39
121.20
119.42
119.02
111.44
82.49
78.71
77.16 CDCl3
29.25
28.93
28.60
28.28
A (q)
122.30
B (q)
28.76
F
F
F
N
N
O
N
CH
220 210 200 190 180 170 160 150 140 130 120 110 100 90 80 70 60 50 40 30 20 10 0 -10
f1 (ppm)

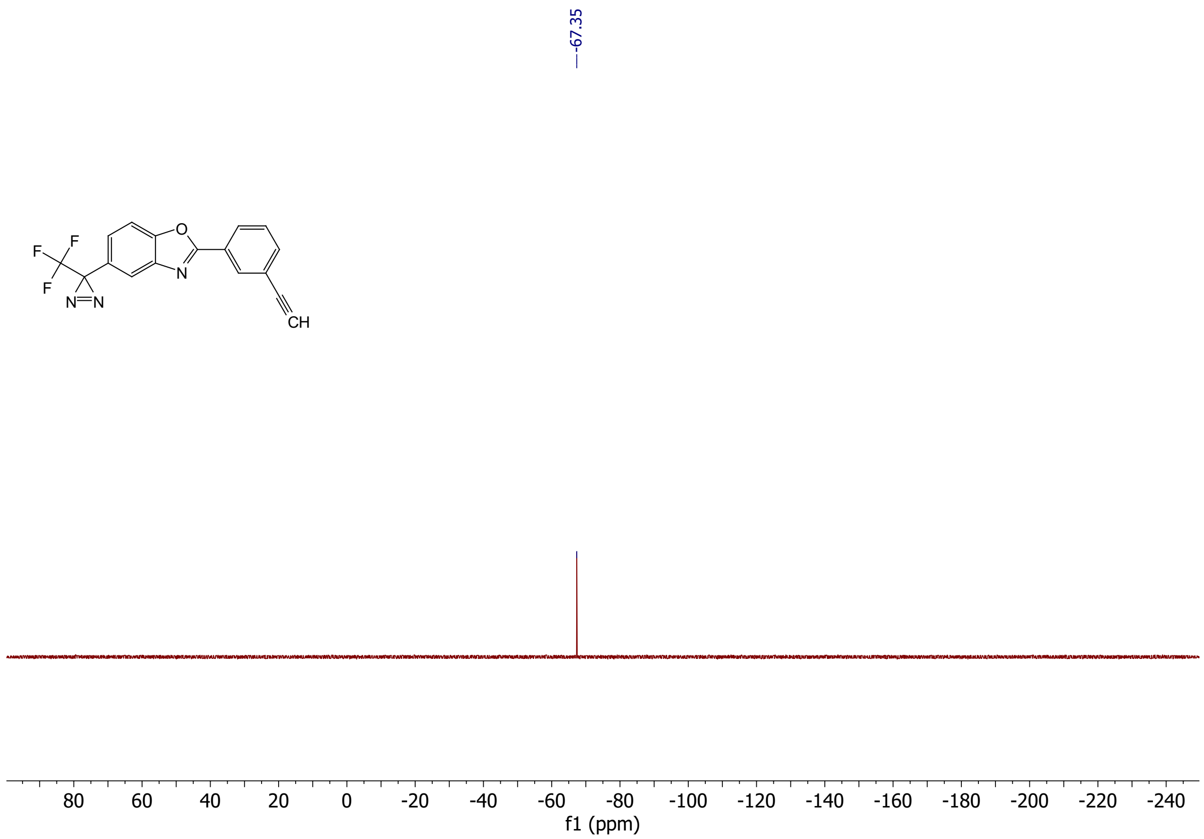
-67.35
F
F
F
N
N
O
N
CH
80 60 40 20 0 -20 -40 -60 -80 -100 -120 -140 -160 -180 -200 -220 -240
f1 (ppm)

2-(5-ethynylnaphthalen-1-yl)-5-(3-(trifluoromethyl)-3H-diazirin-3-yl)benzo[d]oxazole 4

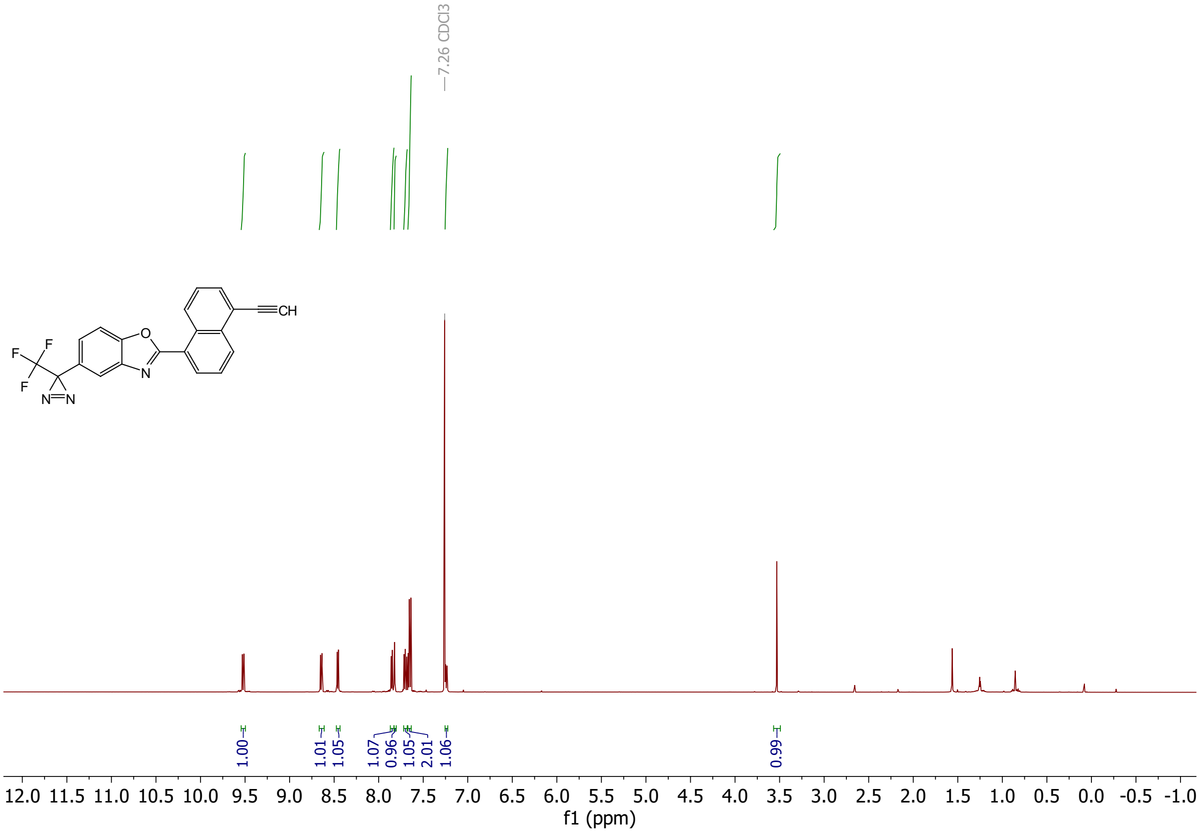

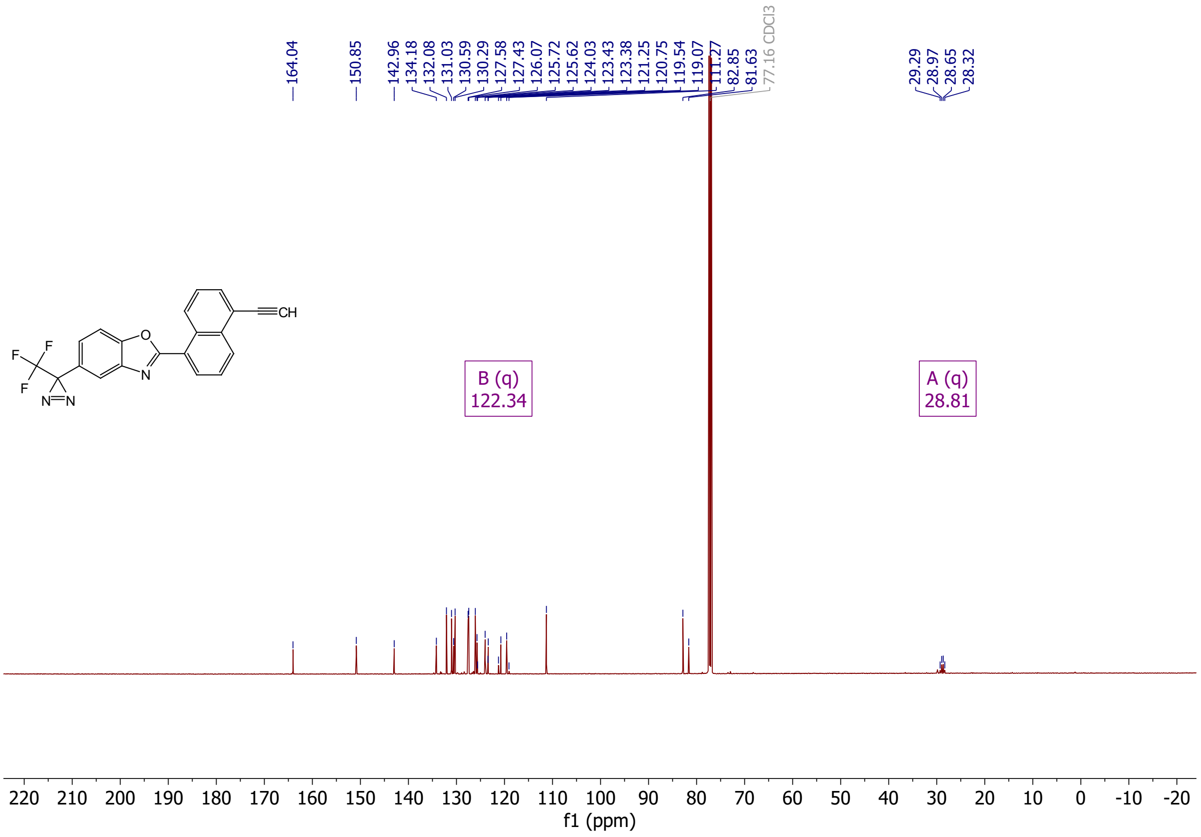

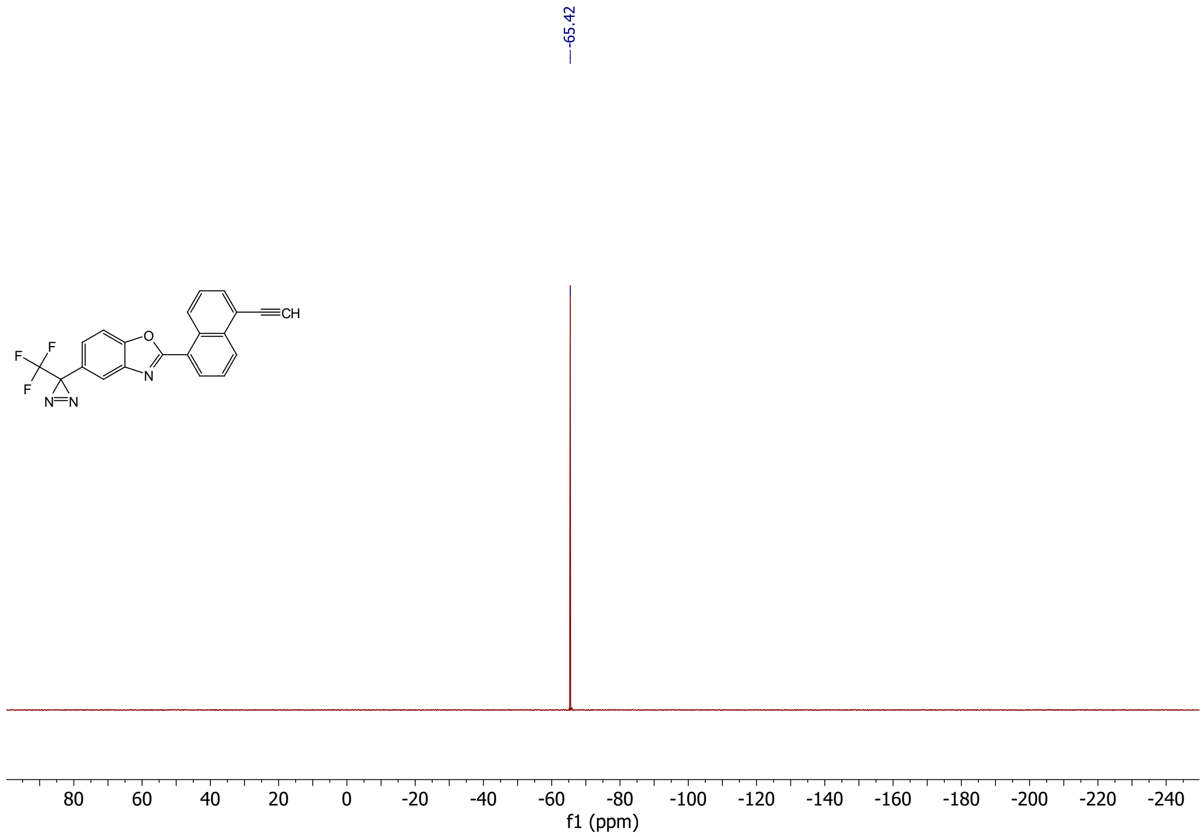

methyl (+)-ethyl(2-(4-fluorophenyl)benzo[d]oxazol-5-yl)phosphinate 7

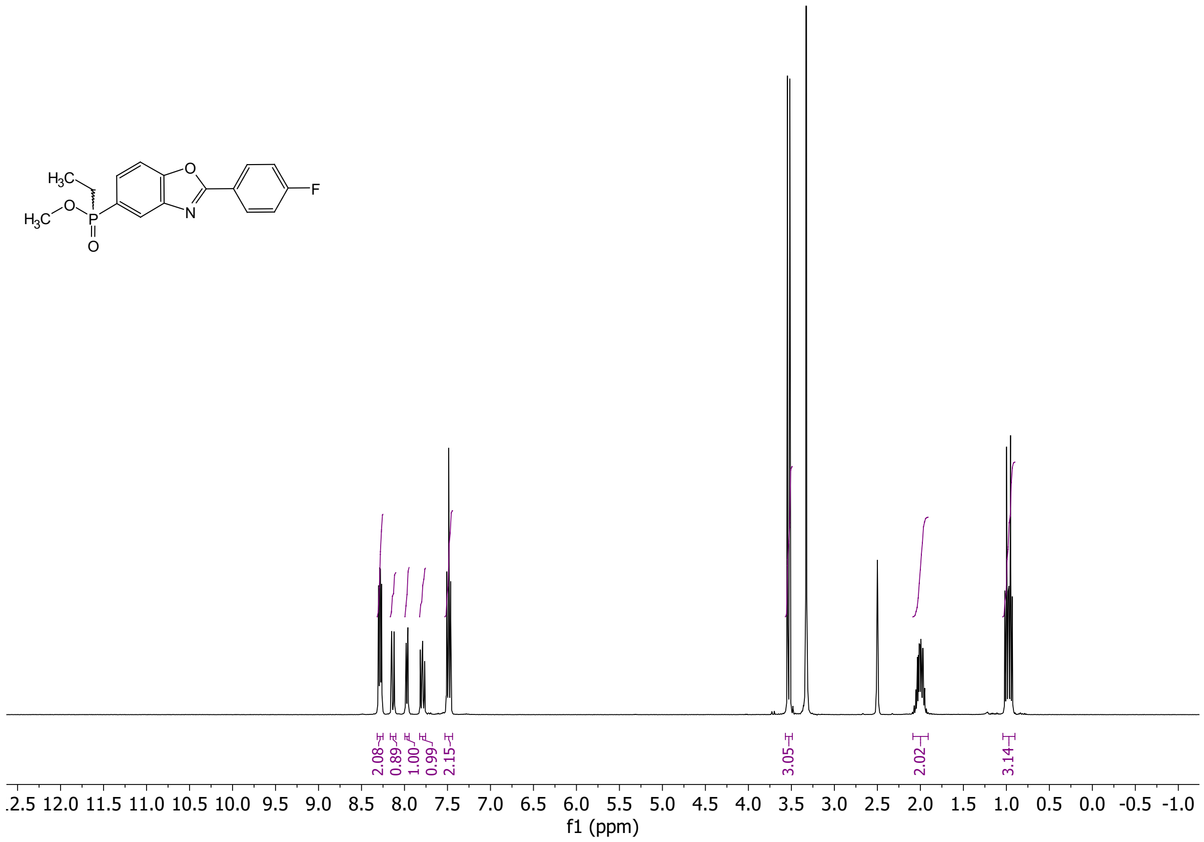

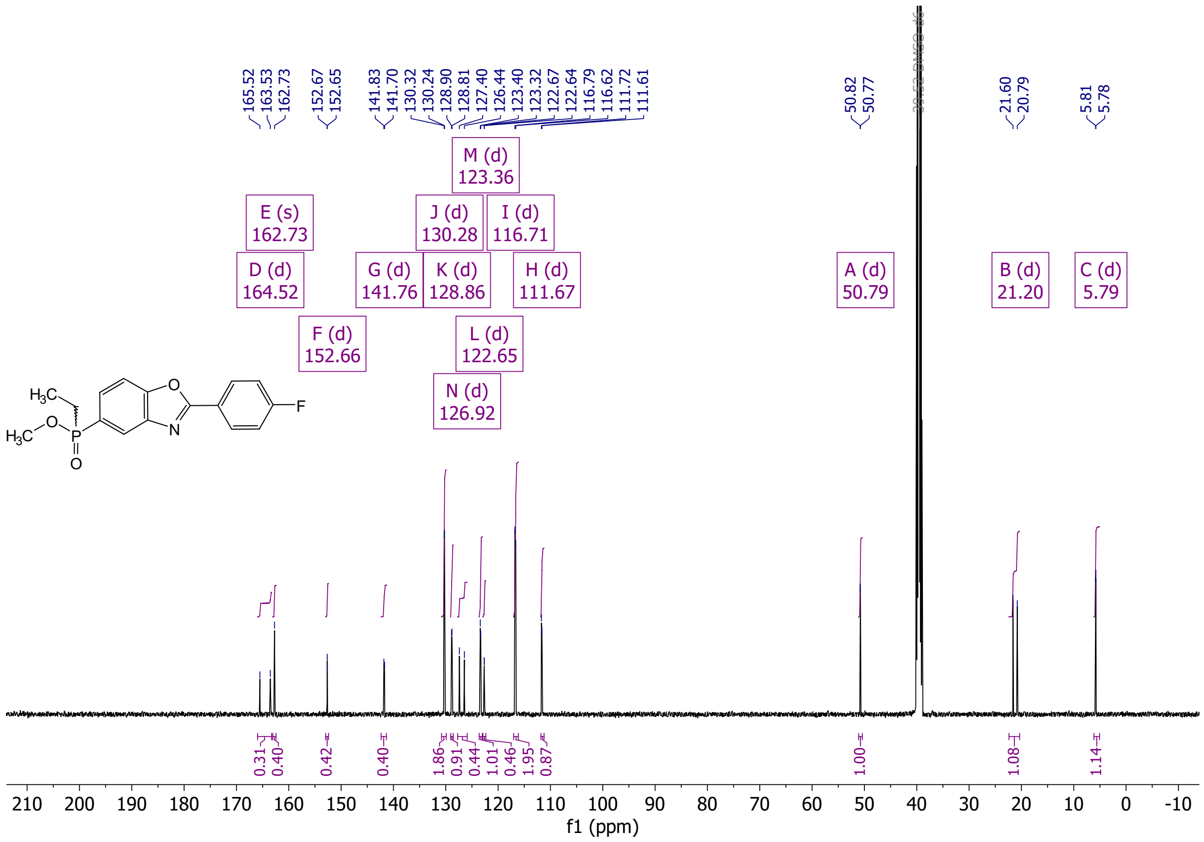
165.52
163.53
162.73
152.67
152.65
141.83
141.70
130.32
130.24
128.90
128.81
127.40
126.44
123.40
123.32
122.67
122.64
116.79
116.62
111.72
111.61
50.82
50.77
21.60
20.79
5.81
5.78
M (d) 123.36
E (s) 162.73
J (d) 130.28
I (d) 116.71
D (d) 164.52
G (d) 141.76
K (d) 128.86
H (d) 111.67
A (d) 50.79
B (d) 21.20
C (d) 5.79
F (d) 152.66
L (d) 122.65
N (d) 126.92
0.31
0.40
0.42
0.40
1.86
0.91
0.44
1.01
0.46
1.95
0.87
1.00
1.08
1.14
f1 (ppm)

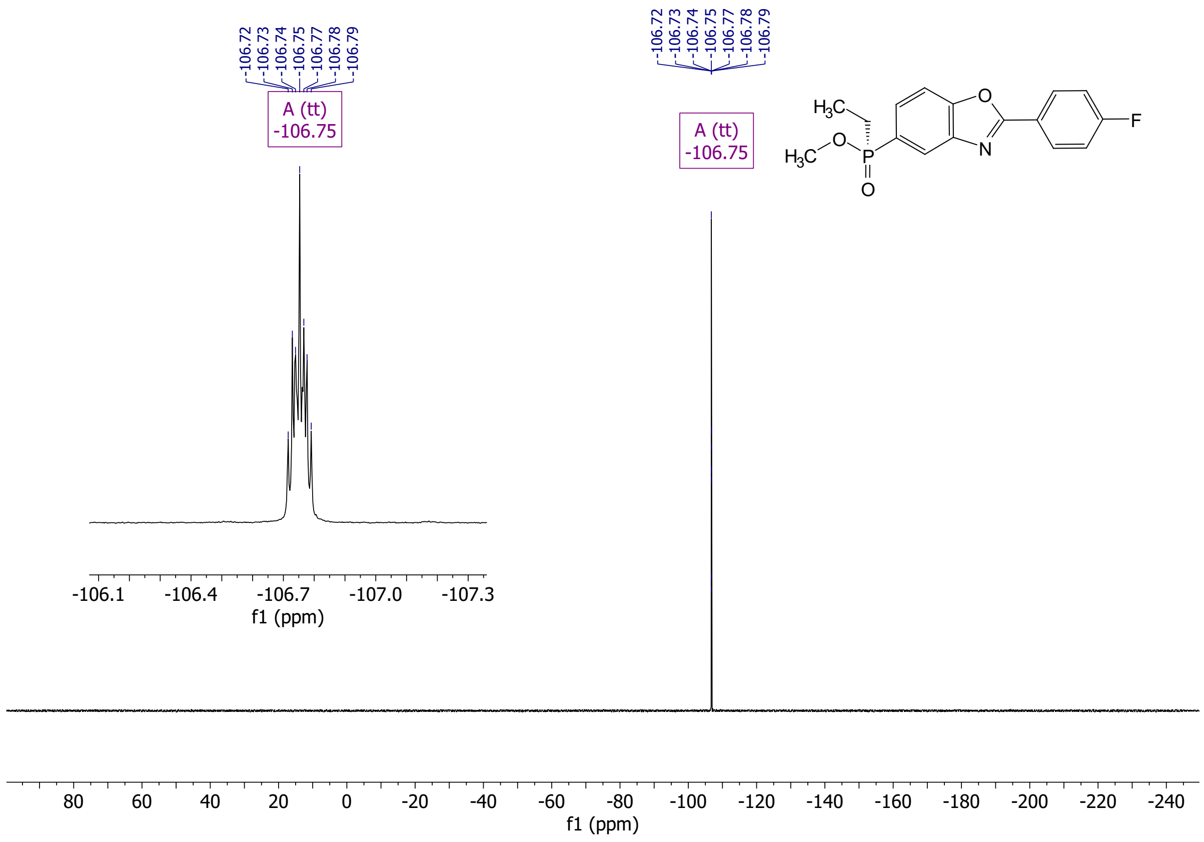
-106.72
-106.73
-106.74
-106.75
-106.77
-106.78
-106.79
A (tt) -106.75
f1 (ppm)

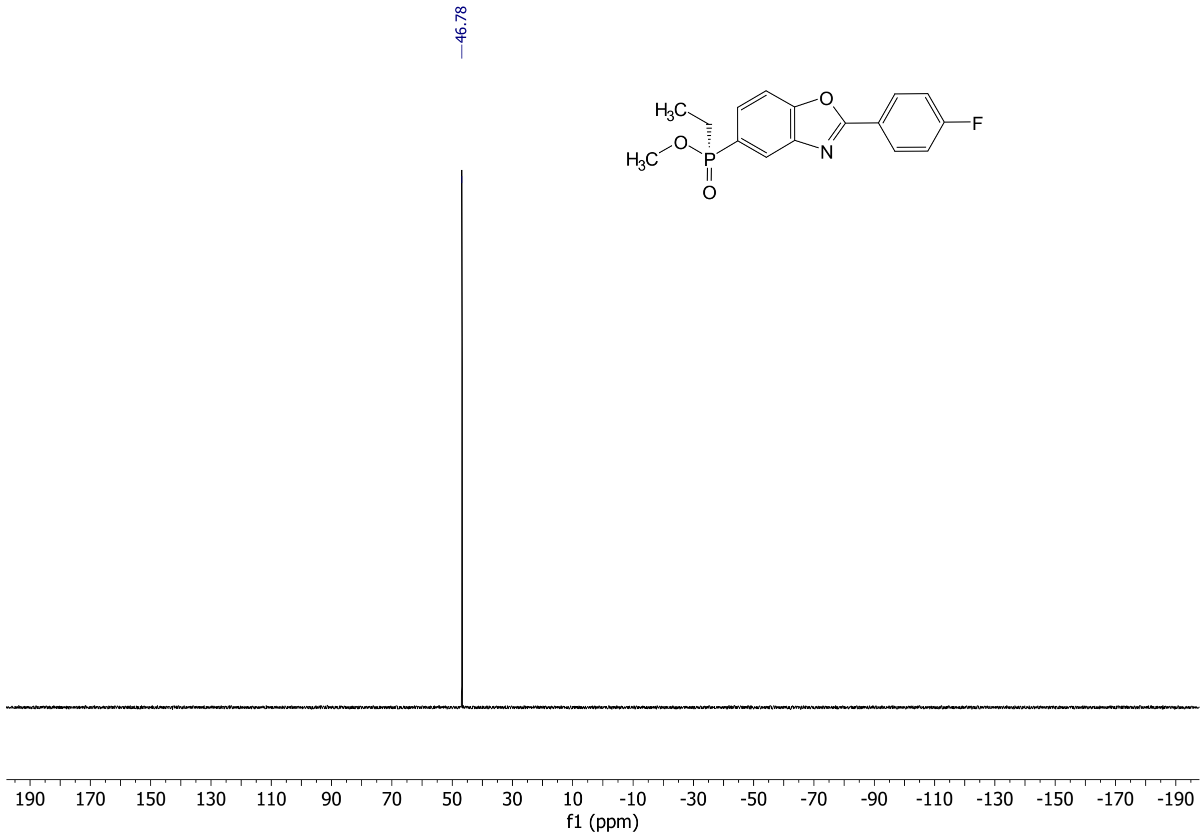
46.78
H3C
H3C
O
P
O
O
N
F
190 170 150 130 110 90 70 50 30 10 -10 -30 -50 -70 -90 -110 -130 -150 -170 -190
f1 (ppm)

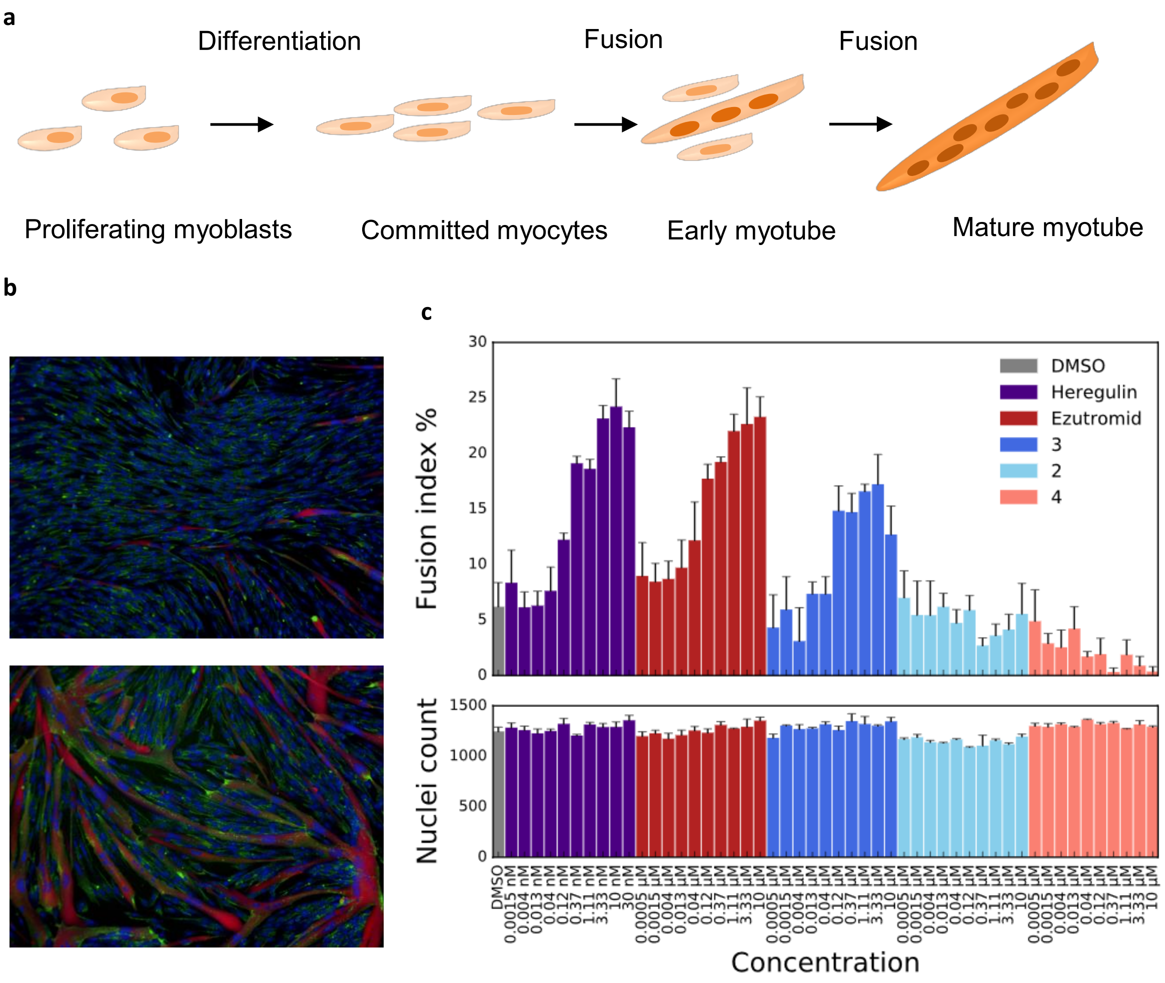

Figure S1. Utrophin modulators such as heregulin, ezutromid and probe 3 increase fusion of DMD myoblasts into mature myotubes. a) Schematic of the process of fusion of proliferating myoblasts into myotubes. **b**) Immortalised DMD myoblasts were treated with compound or vehicle (0.1% DMSO) during five days of differentiation, then fixed and stained for utrophin (green), myosin heavy chain (red) and DAPI (blue). Images taken with a Perkin Elmer Operetta high-content imaging system with a 10 $\times$ objective; top: vehicle control, bottom: 10 μM ezutromid. **c**) Dose-dependent increase in fusion of myoblasts to mature myotubes after 5 days observed after heregulin and ezutromid treatment is also observed after treatment with probe **3**, but not **2** or **4**. Fusion index is shown (top) as mean $\pm$ SD and nuclei count (bottom) mean $\pm$ SD , each n = 4.

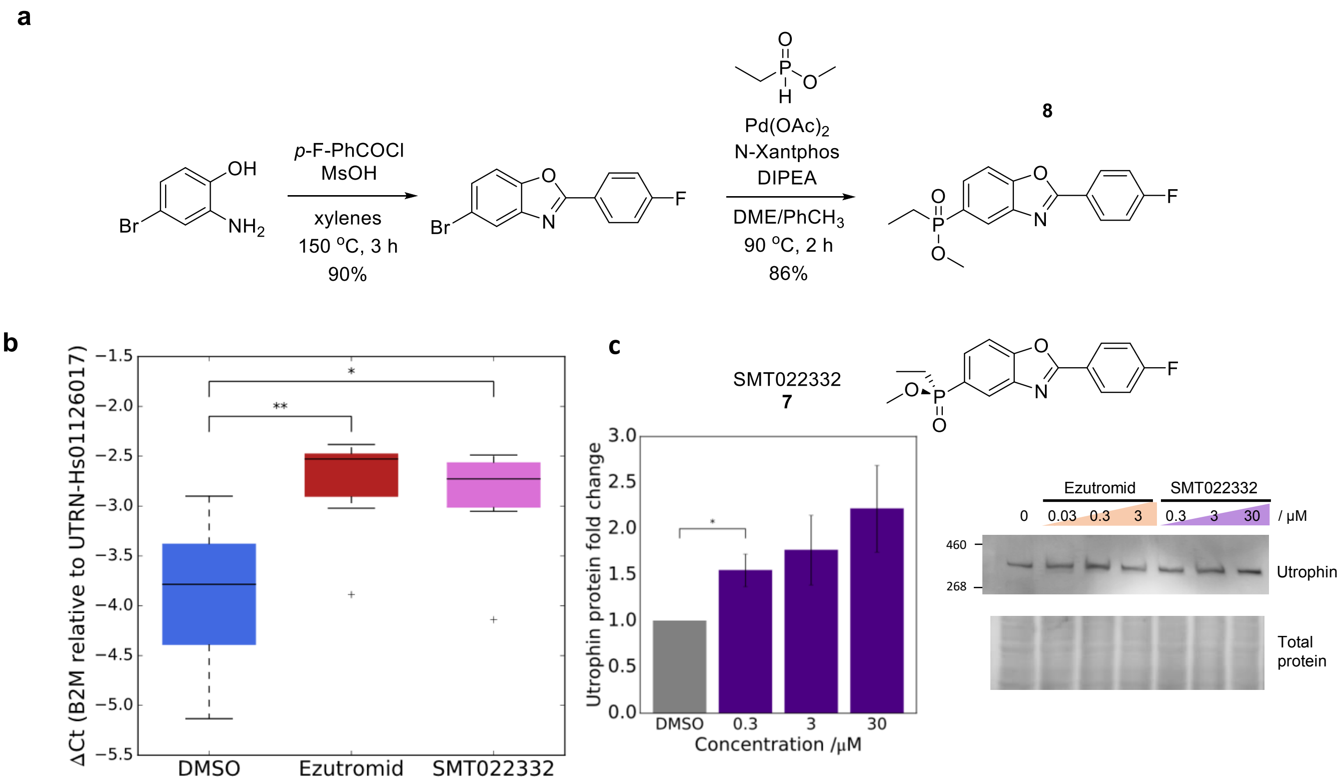

Figure S2. Second generation compound SMT022332 7 upregulates utrophin mRNA and protein levels, to a similar degree as the first generation compound ezutromid. **a**) Synthesis of second generation compound **7**; chiral separation of racemic compound **8** yielded the two enantiomers. **b**) Ezutromid and **7** treatment (each 0.3 μM, 48 h incubation) increases utrophin mRNA expression (normalized to B2M), n = 6. * denotes $p < 0.05$, ** is $p < 0.005$. **c**) SMT022332 increases utrophin protein expression comparable to ezutromid after 24 h of compound treatment in human DMD myoblasts, determined by Western blot. Bars represent means of utrophin expression normalised to total protein and relative to DMSO, error bars are standard error of the mean, n = 4, * denotes $p < 0.05$. Representative blot shown right, uncropped blot presented in Supplementary Fig. 8

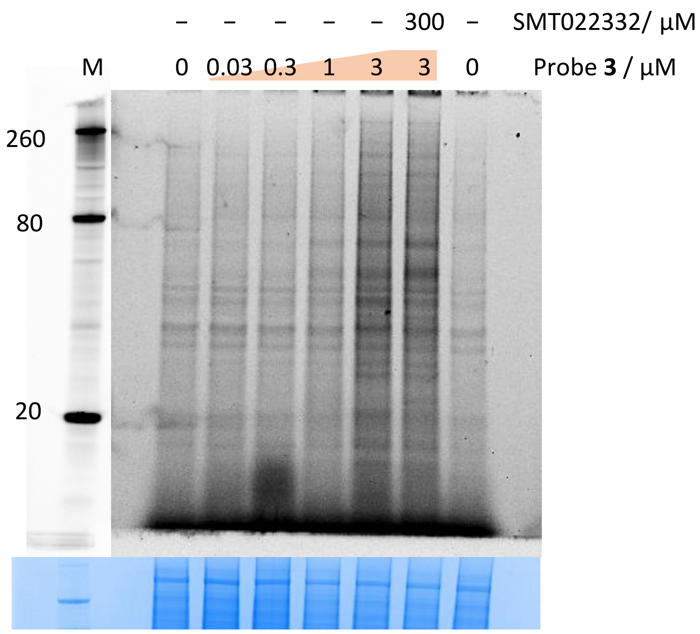

Figure S3. Probe 3 shows concentration-dependent labelling of protein targets. H2K *mdx* cells were treated with **3** (2 h) and irradiated (365 nm, 3 min, 0 °C) to trigger crosslinking. Probe labelled proteins were ligated to TAMRA, separated by SDS-PAGE and visualised by in gel fluorescence. M: protein standard, bottom: Coomassie staining as loading control.

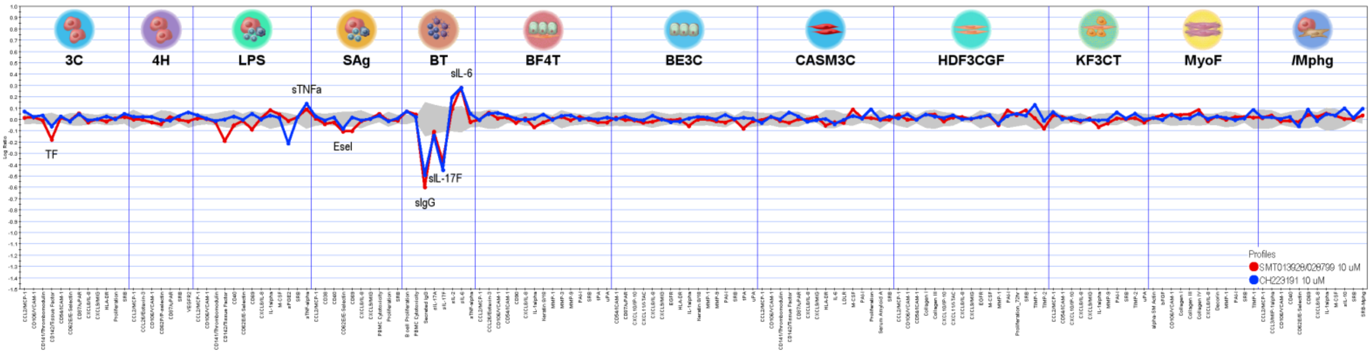

Figure S4. Ezutromid was profiled in the DiscoverX BioMap Diversity PLUS panel, which monitors changes in activity of 148 biomarkers across 12 different human primary cell-based co-culture systems upon compound treatment, and compares the signature with a reference database of compounds with known mechanism. The profile of ezutromid (10 µM, red) best matched the profile of AhR antagonist CH223191 (10 µM, blue), Pearson correlation of 0.854. Grey profiles represent baseline variation, biomarkers with activity deviating from the baseline are annotated.

Figure S5. Uncropped blots from Figure 2

H2K *mdx* cell line

Utrophin

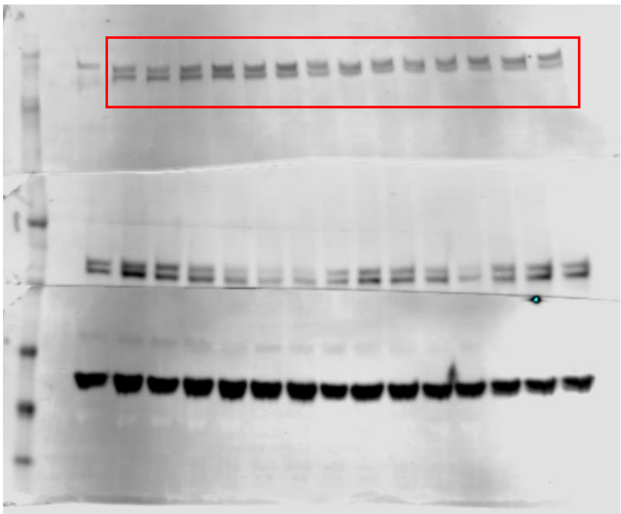

REVERT total protein stain.

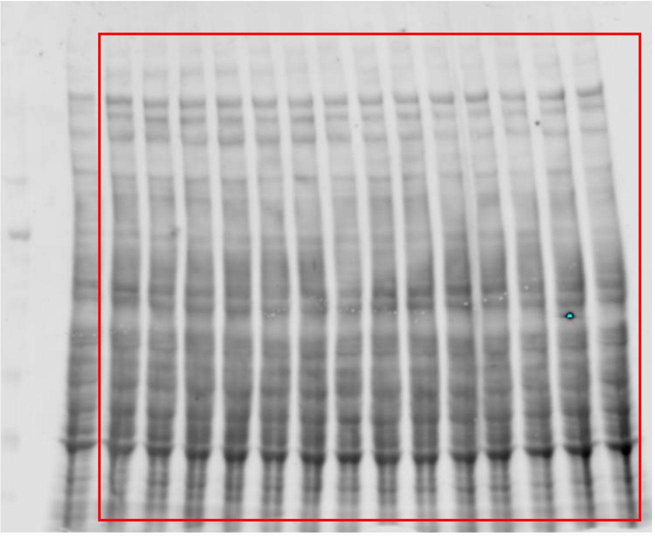

Human DMD cell line

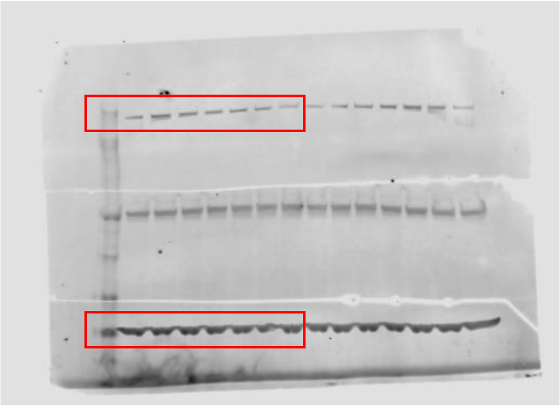

Utrophin

β-actin

Figure S6. Uncropped blot from Figure 4

Human DMD cell line

Anti-AhR antibody staining

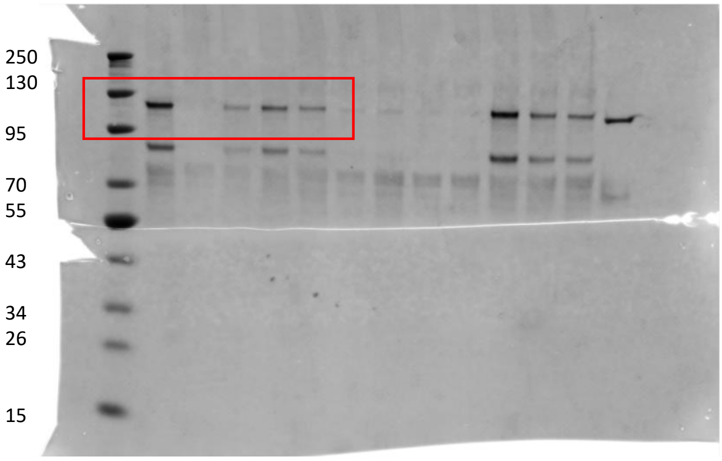

Figure S7. Uncropped blots from Figure 6

Human DMD cell line

Utrophin

AhR

β-tubulin

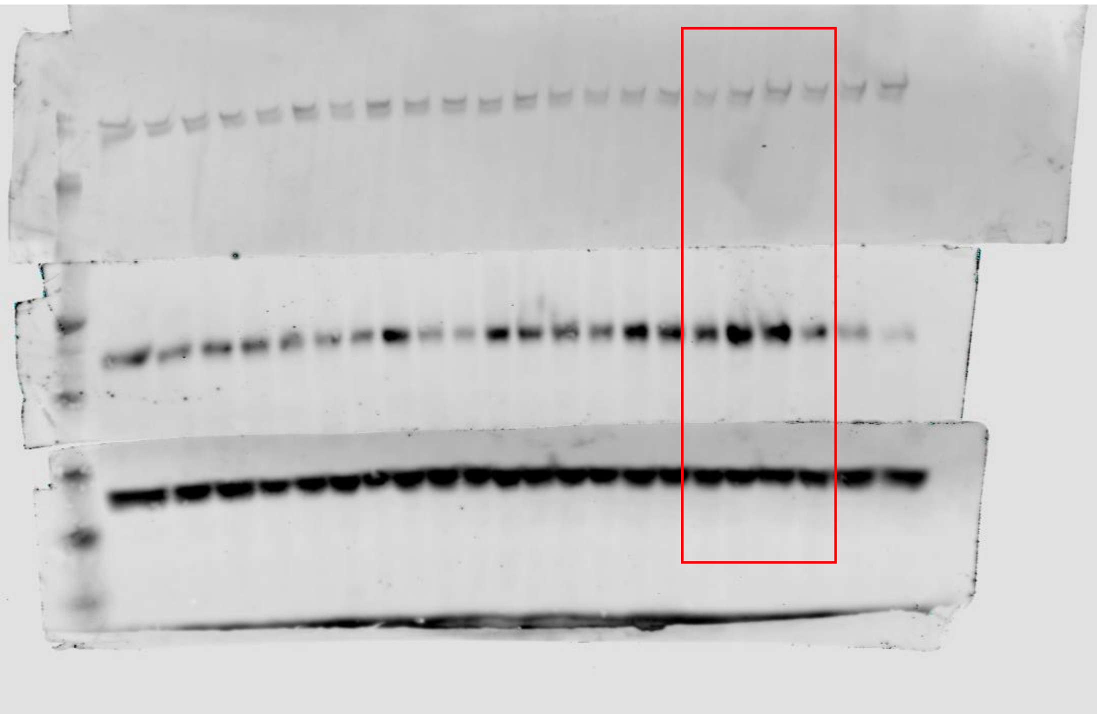

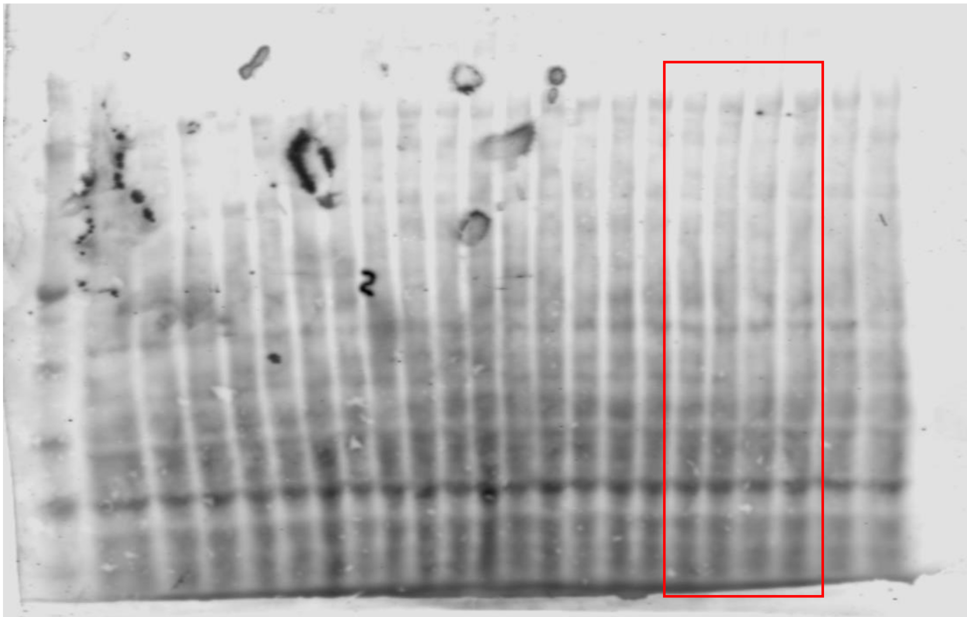

REVERT total protein stain.

Figure S8. Uncropped blots from Supplementary Figure 2

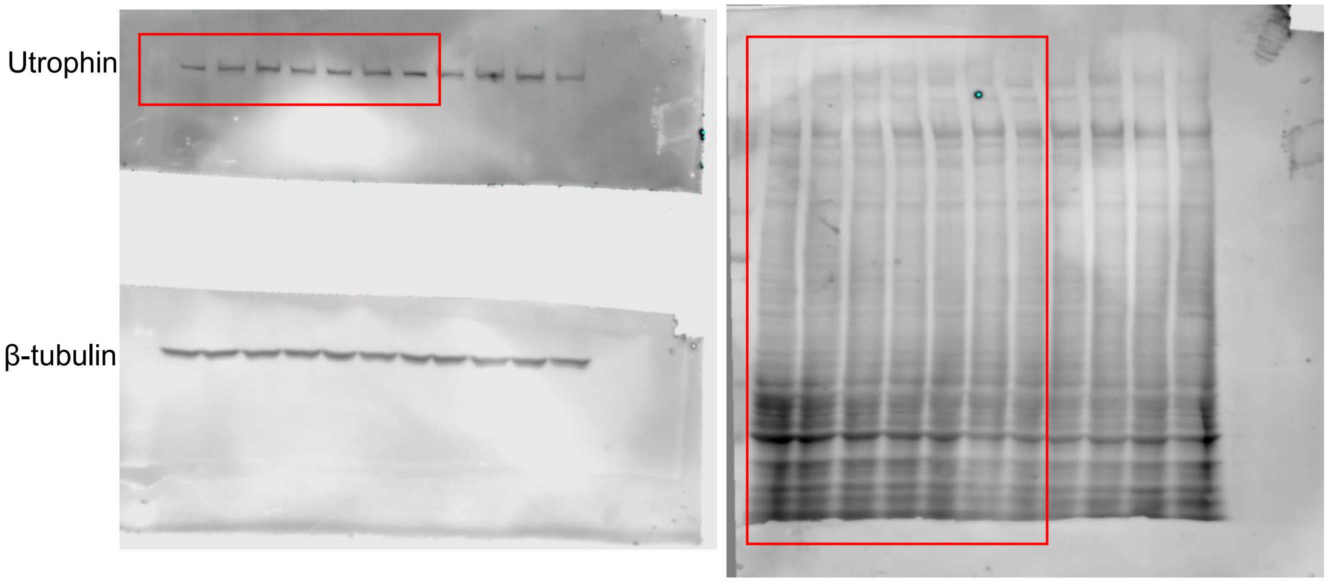

REVERT total protein stain.

References

[1] J. E. Morgan, J. R. Beauchamp, C. N. Pagel, M. Peckham, P. Ataliotis, P. S. Jat, M. D. Noble, K. Farmer, T. A. Partridge, *Dev. Biol.* **1994**, *162*, 486–498.

[2] G. M. Wynne, O. De Moor, C. Lecci, R. Van Well, P. D. Johnson, R. Storer, S. Poignant, S. P. Wren, *Benzoxazole, Benzimidazole and Benzothiazole Derivatives and Their Use in the Treatment of Duchenne Muscular Dystrophy*, **2009**, WO/2009/021750.

[3] J. M. Tinsley, R. J. Fairclough, R. Storer, F. J. Wilkes, A. C. Potter, S. E. Squire, D. S. Powell, A. Cozzoli, R. F. Capogrosso, A. Lambert, et al., *PLoS One* **2011**, *6*, e19189.

[4] K. J. Livak, T. D. Schmittgen, *Methods* **2001**, *25*, 402–408.

[5] W. P. Heal, M. H. Wright, E. Thinon, E. W. Tate, *Nat. Protoc.* **2012**, *7*, 105–117.

[6] J. R. Wiśniewski, A. Zougman, N. Nagaraj, M. Mann, *Nat. Methods* **2009**, *6*, 359–362.

[7] J. Cox, M. Mann, *Nat. Biotechnol.* **2008**, *26*, 1367–1372.

[8] D. Wu, N. Potluri, J. Lu, Y. Kim, F. Rastinejad, *Nature* **2015**, *524*, 303–308.

[9] D. Wu, X. Su, N. Potluri, Y. Kim, F. Rastinejad, *Elife* **2016**, *5*, DOI 10.7554/eLife.18790.

[10] D. M. Rudzinski, C. B. Kelly, N. E. Leadbeater, *Chem. Commun.* **2012**, *48*, 9610–9612.

				Active probe 3			Inactive probe 4			Active probe 3 + competitor 7																
Gene names	Protein names	Protein IDs	Majority protein IDs	1	2	3	1	2	3	1	2	3	Peptides	Razor + unique peptides	Unique peptides	Sequence coverage [%]	Unique + razor sequence coverage [%]	Unique sequence coverage [%]	Mol. weight [kDa]	Q-value	Intensity	MS/MS Count	-Log T-test p-value 3 vs 4	Student's T-test Difference 3 vs 4	-Log Student's T-test p-value 3 vs 3 + 7	Student's T-test Difference 3 vs 3 + 7
4732456N10Rik		E9Q1Z0	E9Q1Z0	17.5	21.5469	22.4186	22.5255	21.2353	17.5	17.5	17.5	17.5	17	1	0	16.2	2.2	0	58.224	1	28891000	1	0.010525	0.0682379	0.921315	2.98852
Acaca	Acetyl-CoA carboxylase 1;Biotin carl	Q5SWU9;Q5SWU	Q5SWU9;Q5SWU9-2	17.5	30.3583	30.2624	29.5646	30.2657	30.0961	26.6333	17.5	26.0209	62	62	56	31.9	31.9	28.8	265.25	0	6.21E+09	300	0.387822	-3.93527	0.196702	2.65548
Acacb		E9Q4Z2	E9Q4Z2	17.5	23.3306	22.5018	22.7159	23.3403	17.5	17.5	17.5	17.5	7	1	1	3.9	0.4	0.4	275.75	1	33998000	3	0.00945559	-0.07459	0.926503	3.6108
Acot7	Cytosolic acyl coenzyme A thioester	Q91V12;Q91V12	Q91V12;Q91V12-2;Q91V12	17.5	22.634	17.5	17.5	17.5	17.5	17.5	17.5	22.5037	2	2	2	3.1	3.1	3.1	42.536	0.004057	15183000	2	0.427243	1.71132	0.00595951	0.0434265
Acta1;Actc1;Acta2;Actg2	Actin, alpha skeletal muscle;Actin, a	P68134;P68033	P68134;P68033;P62737;P	23.3701	24.732	24.3592	25.7192	25.627	27.0194	20.2375	23.7492	24.1486	22	4	4	48.3	19.9	19.9	42.051	0	4.02E+08	13	1.50253	-1.96807	0.478998	1.442
Actb;Actg1	Actin, cytoplasmic 1;Actin, cytoplas	P60710;P63260	P60710;P63260;E9Q1F2;E	29.1471	31.0347	31.6311	31.5161	31.9012	31.3296	30.5149	31.1388	32.179	28	28	10	67.2	67.2	36.8	41.736	0	3.38E+10	715	0.566227	-0.977982	0.307596	-0.673251
Actn1	Alpha-actinin-1	Q7TPR4;A1BN54	Q7TPR4;A1BN54	20.5484	23.0159	22.7926	23.4483	17.5	23.2429	17.5	17.5	24.8553	12	7	7	17.8	11	11	103.07	0	80344000	11	0.125728	0.721897	0.349288	2.16719
Actn4	Alpha-actinin-4	P57780;E9Q2W	P57780	21.5434	24.5164	24.9859	25.5951	23.5769	24.7175	17.5	24.0792	25.9508	13	13	8	19.3	19.3	12.6	104.98	0	2.85E+08	32	0.316467	-0.947946	0.158001	1.17191
Aga	N(4)-(beta-N-acetylglucosaminyl)-L-	B7ZNK6;Q64191	B7ZNK6;Q64191	17.5	17.5	17.5	17.5	17.5	17.5	17.5	17.5	17.5	1	1	1	3.6	3.6	3.6	35.925	0.004065	4887600	1	-4.34E-08	0	-4.34E-08	0
Ahcy	Adenosylhomocysteinase	P50247;A2ALT5	P50247;A2ALT5	21.4763	17.5	23.421	23.6171	17.5	17.5	17.5	22.4471	23.5394	2	2	2	5.6	5.6	5.6	47.688	0	54908000	9	0.178509	1.26006	0.0488846	-0.363092
Ahnak		E9Q616	E9Q616	27.8561	27.8526	28.0592	28.1962	27.049	27.9803	25.0241	28.0702	28.2138	66	66	66	38.1	38.1	38.1	604.25	0	3.9E+09	229	0.193481	0.180852	0.322859	0.819949
Ahnak2		F7CVJ5;F7DBB3;	F7CVJ5;F7DBB3;E9PYB0	17.5	17.5	17.5	17.5	17.5	22.8498	17.5	22.6427	21.1073	4	4	4	40.9	40.9	40.9	115.14	0	30464000	6	0.427243	-1.78327	0.892051	-2.91665
Ahr	Aryl hydrocarbon receptor	P30561;Q3U5D	P30561;Q3U5D9	17.5	24.6702	24.8291	17.5	17.5	17.5	20.0222	17.5	17.5	3	3	3	5	5	5	95.016	0	57569000	4	0.934927	4.83311	0.712763	3.99239
Akr1b1	Aldose reductase	P45376	P45376	22.3209	22.0259	21.8716	23.8989	23.6403	23.4945	17.5	24.2327	24.6706	1	1	1	4.4	4.4	4.4	35.732	0	1.38E+08	14	3.08558	-1.60511	0.00872494	-0.0616506
Alb	Serum albumin	P07724	P07724	17.5	24.4972	17.5	17.5	28.2043	17.5	17.5	17.5	17.5	6	5	5	12.5	10.2	10.2	68.692	0	3.93E+08	7	0.104405	-1.23571	0.427243	2.33239
Aldh2	Aldehyde dehydrogenase, mitochor	P47738;A0A0G2	P47738;A0A0G2JF60;A0A0	17.5	17.5	17.5	21.9299	17.5	17.5	17.5	17.5	22.5645	1	1	1	2.7	2.7	2.7	56.537	0	10198000	3	0.427243	-1.47662	0.427243	-1.68818
Aldh9a1	4-trimethylaminobutyraldehyde de	Q9JLJ2;Q3U367	Q9JLJ2;Q3U367	17.5	17.5	17.5	17.5	20.603	17.5	17.5	17.5	17.5	1	1	1	3	3	3	53.514	0.003992	15627000	1	0.427243	-1.03435	-4.34E-08	0
Aldoa;Aldoart1	Fructose-bisphosphate aldolase A;Fr	P05064;A6ZI44;	P05064;A6ZI44;A0A0U1RP	23.2289	25.8336	26.1552	26.3057	24.0402	22.3996	21.8198	25.8045	27.1996	12	12	12	38.7	38.7	38.7	39.355	0	6.1E+08	34	0.21942	0.824048	0.0236002	0.131249
Anxa1	Annexin A1	P10107	P10107	24.6384	25.8524	25.0098	25.5089	24.5549	23.5586	23.4966	24.8885	25.748	7	7	7	30.9	30.9	30.9	38.734	0	4.53E+08	33	0.396218	0.626074	0.24027	0.45585
Anxa2	Annexin A2	P07356;B0V2N7	P07356;B0V2N7;B0V2N5;E	25.4227	26.748	28.1859	26.9346	26.1345	26.3159	24.7592	27.0401	27.0087	16	16	16	42.5	42.5	42.5	38.676	0	1.43E+09	134	0.144184	0.323874	0.178561	0.516232
Anxa5	Annexin A5	P48036;A0A0G2	P48036;A0A0G2JGQ0	17.5	17.5	17.5	20.3826	17.5	17.5	17.5	21.5817	22.5759	2	2	2	6.6	6.6	6.6	35.752	0.002208	14824000	2	0.427243	-0.960861	0.917992	-3.05253
Arf3;Arf1;Arf2	ADP-ribosylation factor 3;ADP-ribos	P61205;P84078	P61205;P84078;Q8BSL7	17.5	17.5	17.5	23.5221	17.5	23.2698	17.5	17.5	24.234	2	2	1	18.8	18.8	7.7	20.601	0	64293000	7	0.934426	-3.93063	0.427243	-2.24468
Arfip1		G5E8V9;E9Q3G5	G5E8V9;E9Q3G5;A2RSX9	19.812	28.0853	28.8273	21.9859	21.8835	21.8532	24.4839	17.5	24.5033	11	11	11	49.1	49.1	49.1	41.518	0	8.31E+08	31	0.563487	3.66732	0.387191	3.41244
Arfip2	Arfaptin-2	Q8K221;A0A1B0	Q8K221;A0A1B0GS63;D3Z	17.5	23.3237	23.2348	17.5	17.5	17.5	17.5	17.5	17.5	2	2	2	7.9	7.9	7.9	37.772	0	20370000	2	0.935018	3.85284	0.935018	3.85284
Arg1	Arginase-1	Q61176	Q61176	21.8659	23.9236	27.3535	17.5	23.8479	22.2512	20.3331	23.6534	23.3377	2	2	2	6.8	6.8	6.8	34.807	0	3.28E+08	8	0.568062	3.18135	0.432691	1.93963
Arglu1	Arginine and glutamate-rich proteir	Q3UL36;Q3UL3	Q3UL36;Q3UL36-2;A0A14	21.4908	17.5	17.5	22.0594	17.5	21.823	17.5	17.5	17.5	2	2	2	5.5	5.5	5.5	32.887	0.00409	42586000	3	0.338295	-1.63055	0.427243	1.33026
Arhgdia	Rho GDP-dissociation inhibitor 1	Q99PT1	Q99PT1	21.0063	23.4491	23.4569	23.9323	21.6288	21.5907	22.6455	22.0689	24.0119	3	3	3	23.5	23.5	23.5	23.407	0	96334000	10	0.0795145	0.253505	0.0973047	-0.271334
Arpc1b	Actin-related protein 2/3 complex s	Q9WV32;F6THG	Q9WV32;F6THG2;Q91Z25	17.5	21.9345	17.5	20.764	21.4578	21.0513	19.7165	21.4534	17.5	2	2	2	8.3	8.3	8.3	41.063	0.003929	17256000	3	0.638978	-2.11287	0.112156	-0.578487
Atic	Bifunctional purine biosynthesis pr	Q9CWJ9	Q9CWJ9	17.5	21.5898	21.3223	23.0933	17.5	20.7674	17.5	17.5	22.3674	2	2	2	5.2	5.2	5.2	64.217	0.004211	28863000	2	0.0519866	-0.316184	0.185102	1.01492
Atp1a1	Sodium/potassium-transporting AT	Q8VDN2;Q6PIC	Q8VDN2	17.5	23.0901	23.9951	22.6467	17.5	22.5759	17.5	21.3901	23.5003	3	3	3	4.9	4.9	4.9	112.98	0	60593000	4	0.0828445	0.620875	0.0975593	0.731588
Atp2a2	Sarcoplasmic/endoplasmic reticulu	O55143;O5514	O55143;O55143-2	17.5	24.7591	25.1144	23.5844	17.5	23.8863	19.8616	17.5	23.1455	8	8	8	12	12	12	114.86	0	1.21E+08	9	0.0878861	0.800944	0.314936	2.28881
Atp5a1	ATP synthase subunit alpha, mitoch	Q03265;D3Z6F5	Q03265;D3Z6F5	24.4432	22.9364	23.6882	26.0368	25.0979	26.2151	17.5	24.4347	27.065	9	9	9	20.1	20.1	20.1	59.752	0	5.57E+08	22	1.7059	-2.09401	0.0846336	0.689369
Atp5b	ATP synthase subunit beta, mitocho	P56480	P56480	22.743	23.1001	23.001	23.699	17.5	17.5	17.5	21.9422	24.4352	4	4	4	12.7	12.7	12.7	56.3	0	75125000	7	0.750771	3.38171	0.33655	1.65558
Atp5o	ATP synthase subunit O, mitochond	Q9DB20;F7D3P	Q9DB20;F7D3P8	17.5	17.5	22.61	17.5	17.5	23.3776	17.5	17.5	22.1454	1	1	1	5.2	5.2	5.2	23.363	0.00396	24205000	3	0.0332779	-0.255845	0.0224638	0.15487
Atpif1	ATPase inhibitor, mitochondrial	O35143;E9PV44	O35143;E9PV44	17.5	17.5	17.5	17.5	17.5	17.5	17.5	17.5	22.3432	2	2	2	17.9	17.9	17.9	12.159	0	5320600	2	-4.34E-08	0	0.427243	-1.61439
Atxn2l	Ataxin-2-like protein	Q7TQH0;Q7TQH	Q7TQH0;Q7TQH0-2;E9Q5Q	20.6786	20.5561	17.5	17.5	17.5	17.5	17.5	17.5	22.1726	1	1	1	1.6	1.6	1.6	110.65	0.004376	9419500	0	0.934532	2.07822	0.099761	0.520672
Basp1	Brain acid soluble protein 1	Q91XV3	Q91XV3	22.2464	23.5496	17.5	24.3318	17.5	17.5	21.531	24.5971	25.3988	6	6	6	61.9	61.9	61.9	22.086	0	3.94E+08	29	0.170683	1.32139	0.557016	-2.74364
Bclaf1	Bcl-2-associated transcription facto	Q8K019;Q8K01	Q8K019;Q8K019-3;A0A087	21.6136	22.0713	22.6187	22.7868	17.5	21.8522	20.9961	23.0116	22.8298	6	6	6	8.5	8.5	8.5	106	0	85754000	11	0.348028	1.3882	0.0896591	-0.177955
Bst2	Bone marrow stromal antigen 2	Q8R2Q8	Q8R2Q8	21.5023	24.1272	17.5	23.9431	23.6674	22.8786	26.5536	17.5	22.3941	2	2	2	17.4	17.4	17.4	19.152	0	1.62E+08	3	0.55683	-2.45321	0.124543	-1.10606
C030048H21Rik		F6VUL6	F6VUL6	21.1783	23.2416	25.5157	22.4476	22.0184	20.9558	17.5	22.5565	20.6058	1	1	1	5	5	5	24.547	0.00381	97600000	1	0.493811	1.50461	0.7327	3.09109
Cad		B2RQC6;B2RQC	B2RQC6;B2RQC6-2;E9QAI5	21.2901	20.3975	17.5	22.8774	17.5	24.9256	17.5	17.5	24.7763	6	6	6	4.7	4.7	4.7	243.24	0	75729000	6	0.337874	-2.03847	0.0244872	-0.196246
Cald1		E9QA16;Q8VCQ	E9QA16;Q8VCQ8;E9Q0M9;	24.6157	24.3121	24.1624	25.5263	24.2967	24.4157	22.0551	25.6189	26.7614	11	11	1	28	28	2.4	61.836	0	4.68E+08	44	0.390344	-0.382848	0.114345	-0.448393
Calr	Calreticulin	P14211	P14211	20.3444	22.8038	22.493	24.4901	17.5	22.0571	17.5	23.1285	21.7302	3	3	3	11.8	11.8	11.8	47.994	0	64708000	11	0.0860734	0.531339	0.230617	1.0942
Camk2a	Calcium/calmodulin-dependent pro	P11798;F8WHB	P11798;F8WHB5;P11798-	25.4401	17.5	17.5	17.5	17.5	17.5	17.5	17.5	17.5	1	1	1	4.2	4.2	4.2	54.114	0.005587	45522000	2	0.427243	2.64669	0.427243	2.64669
Cand1	Cullin-associated NEDD8-dissociate	Q6ZQ38	Q6ZQ38	17.5	21.0604	21.4986	22.7675	22.1516	23.9069	20.6434	17.5	23.0871	4	4	4	4.4	4.4	4.4	136.33	0	45336000	6	1.0032	-2.92232	0.0662764	-0.390486
Cap1	Adenylyl cyclase-associated protein	P40124	P40124	17.5	17.5	17.5	21.0472	17.5	17.5	17.5	17.5	23.6931	1	1	1	3.8	3.8	3.8	51.564	0.005682	30841000	4	0.427243	-1.1824	0.427243	-2.06436
Capg	Macrophage-capping protein	P24452;Q99LB4	P24452;Q99LB4	17.5	20.4403	21.3827	21.1558	17.5	20.8589	17.5	17.5	21.1736	1	1	1	3.7	3.7	3.7	39.24	0.003984	11617000	1	0.0127463	-0.0638701	0.245038	1.04982
Caprin1	Caprin-1	Q60865	Q60865	20.8272	22.6853	22.7673	22.8277	17.5	17.5	17.5	22.4514	23.6175	2	2	2	3.3	3.3	3.3	78.168	0.002336	49795000	6	0.678967	2.81736	0.17282	0.903624
Capza2	F-actin-capping protein subunit alp	P47754;A0A0N4	P47754;A0A0N4SVM0;D6R	21.8352	23.351	22.7948	22.3986	17.5	20.8937	17.5	17.5	24.0006	3	3	3	14.7	14.7	14.7	32.967	0	52580000	7	0.723874	2.39622	0.606741	2.99348
Cat	Catalase	P24270	P24270	20.8954	20.7188	23.2528	17.5	17.5	17.5	17.5	17.5	17.5	2	2	2	4.4	4.4	4.4	59.795	0	22253000	2	2.13977	4.12234	2.13977	4.12234
Cav1;Cav3	Caveolin;Caveolin-1;Caveolin-3	D3Z0J2;P49817	D3Z0J2;P49817-2;P49817;	20.6392	22.9865	24.6282	21.1839	17.5	17.5	21.5971	17.5	22.5441	2	2	2	36.2	36.2	36.2	5.3828	0	48819000	2	1.12122	4.02336	0.497906	2.20423
Cbx3	Chromobox protein homolog 3	P23198;D3Z1A9	P23198;D3Z1A9;D3Z313;Q	17.5	22.1161	23.0136	21.8731	22.1627	21.4377	17.5	22.6579	23.1275	1	1	1	7.7	7.7	7.7	20.855	0	55341000	5	0.213902	-0.947945	0.0296062	-0.218553
Cct2	T-complex protein 1 subunit beta	P80314	P80314	20.1934	22.3921	23.5818	24.752	21.1216	17.5	17.5	21.0645	24.1997	4	4	4	13.1	13.1	13.1	57.477	0	76960000	7	0.149794	0.931214	0.200981	1.13436
Cct3	T-complex protein 1 subunit gamma	P80318;E9Q133	P80318;E9Q133;Q3U0I3	26.3476	30.5801	31.1861	31.1025	29.6051	28.0338	28.2624	30.7773	30.8733	9	9	9	22.4	22.4	22.4	60.629	0	1.54E+10	43	0.0403995	-0.209179	0.125834	-0.59973
Cct4	T-complex protein 1 subunit delta	P80315;G5E839	P80315;G5E839	20.3853	22.6785	17.5	25.0325	25.4146	26.1657	23.0411	25.2308	25.1128	3	3	3	7.8	7.8	7.8	58.066	0	3.03E+08	19	1.59827	-5.34964	1.21098	-4.27359
Cct5	T-complex protein 1 subunit epsilor	P80316;E0CZA1	P80316;E0CZA1	17.5	22.6104	17.5	23.2322	17.5	22.841	17.5	17.5	24.2616	4	4	4	9.2	9.2	9.2	59.623	0	49240000	7	0.324753	-1.98758	0.0680192	-0.550406
Cct6a	T-complex protein 1 subunit zeta	P80317	P80317	20.0626	22.3804	17.5	23.6913	23.539	23.9519	20.8605	17.5	24.2708	5	5	5	13.6	13.6	13.6	58.004	0	81826000	7	1.24351	-3.74634	0.137357	-0.8961
Cct7	T-complex protein 1 subunit eta	P80313;A0A0N4	P80313;A0A0N4SV00	20.9037	21.4044	20.7634	24.072	22.4654	22.8678	17.5	20.1651	23.4366	4	4	4	10.7	10.7	10.7	59.652	0	66715000	1	1.8129	-2.11125	0.140724	0.656609
Cct8	T-complex protein 1 subunit theta	P42932;H3BL49	P42932;H3BL49;H3BJB6	20.8518	22.6906	23.5089	24.0794	25.2356	25.1067	21.7925	23.6982	25.0987	8	8	8	17.9	17.9	17.9	59.555	0	2.01E+08	24	1.3268	-2.45677	0.403295	-1.17934
Cd44	CD44 antigen	P15379;P15379	P15379;P15379-2;Q3U8S1	17.5	20.9627	17.5	21.8125	17.5	20.5395	18.6984	20.4357	21.4314	1	1	1	1.7	1.7	1.7	85.616	0.004292	16440000	1	0.306586	-1.29646	0.473974	-1.53425
Cdc42	Cell division control protein 42 hon	P60766;P60766	P60766;P60766-1	17.5	17.5	21.034	23.0173	17.5	17.5	17.5	17.5	17.5	1	1	1	11	11	11	21.258	0.004246	13026000	2	0.109466	-0.661119	0.427243	1.17799
Cdsn	Corneodesmosin	Q7TPC1	Q7TPC1	20.1337	21.815	24.1248	22.0982	20.4565	17.5	17.5	20.9606	20.2023	1	1	1	1.6	1.6	1.6	54.312	0.003899	49475000	7	0.492973	2.00626	0.723449	2.47023
Cdv3	Protein CDV3	Q4VAA2;A0A087	Q4VAA2;A0A087WNP6;Q4	22.9556	17.5	17.5	17.5	17.5	17.5	17.5	23.0942	23.9544	3	3	3	30.2	30.2	30.2	29.729	0	1.14E+08	7	0.427243	1.81853	0.333015	-2.19765
Cfdp1	Craniofacial development protein 1	O88271	O88271	19.9529	17.5	22.4124	21.313	17.5	17.5	17.5	21.8341	21.8756	2	2	2	12.5	12.5	12.5	32.921	0.002227	25469000	5	0.245841	1.18412	0.0777698	-0.448125
Cfl1	Cofilin-1	P18760;F8WGL	P18760;F8WGL3	24.9091	25.3506	27.0422	26.0031	25.215	25.6381	23.3581	27.2238	27.4433	8	8	8	60.8	60.8	60.8	18.559	0	1.11E+09	57	0.0758177	0.148535	0.0563701	-0.241109
Chchd1;Pip5kl1	Coiled-coil-helix-coiled-coil-helix d	Q9CQA6;Q6U7H	Q9CQA6;Q6U7H8-4;Q6U7H	22.968	17.5	17.5	17.5	17.5	17.5	17.5	17.5	17.5	1	1	1	5.9	5.9	5.9	13.608	1	22114000	1	0.427243	1.82266	0.427243	1.82266
Ckap4	Cytoskeleton-associated protein 4	Q8BMK4	Q8BMK4	22.9082	26.0502	26.8873	25.1427	24.3166	23.9867	24.5952	22.253	25.672	17	17	17	40.2	40.2	40.2	63.691	0	5.99E+08	39	0.252	0.799882	0.283359	1.10849
Clic1	Chloride intracellular channel prote	Q9Z1Q5	Q9Z1Q5	21.3066	23.1227	17.5	17.5	21.2576	17.5	17.5	23.2775	25.0123	3	3	3	23.7	23.7	23.7	27.013	0	1.25E+08	7	0.382831	1.89057	0.173365	-1.28684
Clic4	Chloride intracellular channel prote	Q9QYB1	Q9QYB1	17.5	17.5	17.5	17.5	17.5	17.5	17.5	17.5	23.2433	1	1	1	4.7	4.7	4.7	28.729	0	9929800	1	-4.34E-08	0	0.427243	-1.91444
Cltc	Clathrin heavy chain 1	Q68FD5;Q5SXR6	Q68FD5;Q5SXR6	21.5854	25.1545	25.1011	26.054	22.9417	25.876	17.5	23.2397	26.3265	12	12	12	11.3	11.3	11.3	191.55	0	3.92E+08	46	0.258909	-1.01022	0.21792	1.59161
Cnn3	Calponin-3	Q9DAW9;A0A0G	Q9DAW9;A0A0G2JFC8;A0A	17.5	17.5	17.5	17.5	17.5	22.0175	17.5	17.5	17.5	1	1	1	5.8	5.8	5.8	36.428	0.004202	4245600	1	0.427243	-1.50585	-4.34E-08	0
Col1a1	Collagen alpha-1(I) chain	P11087;P11087	P11087;P11087-2	22.3061	22.5553	17.5	22.2579	22.3591	22.2974	17.5	23.9618	24.1858	10	10	10	14.6	14.6	14.6	138.03	0	1.56E+08	22	0.388773	-1.51767	0.148848	-1.09542
Copa	Coatomer subunit alpha;Xenin;Prox	Q8CIE6;F8WHL2	Q8CIE6;F8WHL2	17.5	17.5	22.2065	23.0527	23.832	26.0077	17.5	17.5	24.244	5	5	5	5.1	5.1	5.1	138.43	0	1.28E+08	7	1.35688	-5.22865	0.0880326	-0.679177
Copg1	Coatomer subunit gamma-1	Q9QZE5	Q9QZE5	17.5	17.5	17.5	17.5	17.5	23.0201	21.5777	17.5	21.9936	2	2	2	3.3	3.3	3.3	97.512	0.004255	18667000	3	0.427243	-1.84002	0.93162	-2.85709
Cyb5b	Cytochrome b5 type B	Q9CQX2	Q9CQX2	21.382	17.5	24.6715	17.5	17.5	17.5	17.5	17.5	17.5	1	1	1	23.3	23.3	23.3	16.318	0	87212000	1	0.823676	3.68449	0.823676	3.68449
Cyb5r3	NADH-cytochrome b5 reductase 3;N	Q9DCN2;A0A0A	Q9DCN2;A0A0A0MQM3;Q9	19.9015	21.3884	22.7157	23.3749	17.5	21.4586	17.5	17.5	22.3507	2	2	2	9.6	9.6	9.6	34.127	0	31068000	3	0.105085	0.557335	0.541338	2.21829
Dad1	Dolichyl-diphosphooligosaccharide	P61804	P61804	17.5	20.7372	22.7916	17.5	17.5	21.7049	17.5	17.5	22.2527	1	1	1	10.6	10.6	10.6	12.497	0	23391000	3	0.278224	1.44129	0.222299	1.25869
Ddx21	Nucleolar RNA helicase 2	Q9JIK5	Q9JIK5	18.6777	24.2995	22.9986	24.6193	23.4442	25.2553	17.5	22.7728	24.0955	11	11	11	17.7	17.7	17.7	93.55	0	1.74E+08	21	0.617805	-2.44769	0.0711846	0.535801
Ddx39b	Spliceosome RNA helicase Ddx39b	Q9Z1N5	Q9Z1N5	19.5961	22.5773	17.5	22.8299	21.6104	17.5	21.2907	21.4934	24.1156	3	3	1	7.7	7.7	3.3	49.035	0	59979000	7	0.126814	-0.755654	0.626113	-2.40878
Ddx3x;D1Pas1;Ddx3y	ATP-dependent RNA helicase DDX3X	Q62167;P16381	Q62167;P16381;Q62095	17.5	20.8187	21.3523	24.9135	24.6881	25.6758	17.5	17.5	24.5033	7	7	6	16.6	16.6	15	73.101	0	1.63E+08	12	1.8599	-5.20209	0.00698793	0.0559158
Ddx46	Probable ATP-dependent RNA helica	Q569Z5;Q569Z5	Q569Z5;Q569Z5-2;F8WHR	25.1045	24.7815	24.1505	24.8887	24.7891	24.6779	25.1285	25.4322	24.5148	2	2	2	2.1	2.1	2.1	117.45	0.002299	4.37E+08	11	0.137046	-0.106387	0.373055	-0.346299
Ddx5	Probable ATP-dependent RNA helica	Q61656;Q8BTS0	Q61656;Q8BTS0;S4R1I6	22.3692	17.5	17.5	24.4232	23.6461	28.1713	19.961	21.0123	23.9138	6	5	4	13	11.2	9.3	69.289	0	3.73E+08	22	1.37164	-6.29047	0.552733	-2.50597
Ddx6	Probable ATP-dependent RNA helica	P54823	P54823	17.5	17.5	17.5	21.6953	17.5	21.7938	17.5	17.5	22.5613	1	1	1	3.9	3.9	3.9	54.191	0.003839	16649000	1	0.934905	-2.82971	0.427243	-1.68711
Degs1	Sphingolipid delta(4)-desaturase DE	O09005	O09005	21.7524	22.1584	22.1187	22.6166	17.5	17.5	17.5	17.5	23.3526	2	2	2	10.5	10.5	10.5	38.241	0	68669000	11	0.753404	2.80432	0.583834	2.55898
Dek	Protein DEK	Q7TNV0;E9Q8Y1	Q7TNV0;E9Q8Y1;D3YVJ6	21.3232	21.5846	17.5	24.3331	17.5	21.4969	17.5	20.473	22.8339	6	6	6	13.7	13.7	13.7	43.158	0	1.61E+08	14	0.152751	-0.974045	0.0218593	-0.133032
Dhx15	Putative pre-mRNA-splicing factor A	O35286;A0A0G2	O35286;A0A0G2JG10;Q49	17.5	17.5	17.5	17.5	17.5	17.5	17.5	21.4668	20.863	2	2	2	3.3	3.3	3.3	91.006	0.00432	4805500	2	-4.34E-08	0	0.925152	-2.44328
Dhx9	ATP-dependent RNA helicase A	O70133;O70133	O70133;O70133-2;O70133	17.5	17.5	22.7939	17.5	17.5	22.3867	17.5	17.5	21.3114	1	1	1	1.2	1.2	1.2	149.47	0	19201000	1	0.0188008	0.13575	0.0802156	0.494195
Dnase1	Deoxyribonuclease-1	P49183;H3BKL7	P49183;H3BKL7;E9Q797	20.6135	17.5	17.5	21.8766	17.5	20.3007	17.5	21.3559	21.592	1	1	1	3.2	3.2	3.2	32.027	0.003891	19917000	2	0.339898	-1.35461	0.405785	-1.61146
Dpysl2;Dpysl3;Crmp1	Dihydropyrimidinase-related protei	O08553;Q6218	O08553;Q62188;P97427;D	17.5	20.6761	22.6467	17.5	21.1704	20.9039	17.5	20.7206	23.42	2	2	2	7.5	7.5	7.5	62.277	0.005608	35745000	2	0.0767124	0.416173	0.0407661	-0.272592
Dsg1b;Dsg1a	Desmoglein-1-beta;Desmoglein-1-al	Q7TSF1;Q61495	Q7TSF1;Q61495	26.1256	25.3714	27.695	23.1178	23.8199	21.3589	17.5	24.3033	24.3803	4	4	4	7.3	7.3	7.3	114.45	0	5.74E+08	29	1.65224	3.63176	0.845544	4.33613
Dsp	Desmoplakin	E9Q557;E9PZW	E9Q557;E9PZW0	29.6596	30.5836	32.4867	28.66	28.9495	26.1776	26.3799	29.3682	28.2176	83	83	83	23.1	23.1	23.1	332.91	0	1.32E+10	459	1.15755	2.98093	1.14099	2.92141
Dstn	Destrin	Q9R0P5	Q9R0P5	17.5	21.4534	22.6831	22.4482	17.5	17.5	17.5	17.5	23.0895	2	2	2	18.2	18.2	18.2	18.521	0	26933000	2	0.242412	1.39608	0.185534	1.18233

Dync1h1	Cytoplasmic dynein 1 heavy chain 1	Q9JHU4	Q9JHU4	19.9095	20.6064	19.6306	22.5184	17.5	21.6579	17.5	17.5	17.5	4	4	4	1.6	1.6	1.6	532.04	0	19045000	5	0.117743	-0.509938	3.03321	2.54882
Dync1i2	Cytoplasmic dynein 1 intermediate	O88487;A2BFF8	O88487;A2BFF8;Q3TPJ8;A2	17.5	20.6201	26.5702	17.5	17.5	17.5	17.5	17.5	17.5	2	2	2	4.9	4.9	4.9	68.393	0	1.01E+08	1	0.695952	4.06343	0.695952	4.06343
Ebp	3-beta-hydroxysteroid-Delta(8),Delt	P70245;A2AC29	P70245;A2AC29	17.5	17.5	17.5	17.5	17.5	17.5	17.5	17.5	20.8623	1	1	1	5.7	5.7	5.7	26.215	0.004008	6980200	2	-4.34E-08	0	0.427243	-1.12076
Eef1a1	Elongation factor 1-alpha 1	P10126;D3Z3I8;	P10126;D3Z3I8;D3YZ68	26.9032	28.3172	28.8008	30.4944	28.874	30.7338	27.8088	28.8422	28.9922	19	19	19	35.5	35.5	35.5	50.113	0	7.9E+09	194	1.16848	-2.02696	0.326877	-0.540661
Eef1b;Eef1b2	Elongation factor 1-beta	O70251;M0QWI	O70251;M0QWK5;M0QWH	17.5	18.1218	22.4995	21.6397	17.5	17.5	17.506	17.5	23.0307	1	1	1	6.7	6.7	6.7	24.693	0.004115	23800000	0	0.0835356	0.49386	0.00380428	0.0281741
Eef1d	Elongation factor 1-delta	P57776;P57776	P57776;P57776-3;D3YUQ5	17.5	21.3745	20.6711	17.5	17.5	17.5	17.5	23.1128	23.73	2	2	2	12.8	12.8	12.8	31.293	0	41359000	5	0.92058	2.34852	0.277945	-1.59909
Eef1g	Elongation factor 1-gamma	Q9D8N0	Q9D8N0	23.0111	24.5284	25.105	26.0441	24.9351	24.6113	22.9432	24.468	25.8936	8	8	8	18.5	18.5	18.5	50.06	0	3.71E+08	35	0.574925	-0.981995	0.0730712	-0.220061
Eef2	Elongation factor 2	P58252;Q0881C	P58252	24.3428	26.4848	26.6509	27.6161	26.9834	25.5138	25.3346	25.8874	27.5985	17	17	17	21.7	21.7	21.7	95.313	0	1.31E+09	113	0.380618	-0.878263	0.167321	-0.447318
Eif2s1	Eukaryotic translation initiation fac	Q6ZWX6	Q6ZWX6	20.7158	21.6619	17.5	22.0519	22.7397	23.7977	17.5	22.4872	23.3962	2	2	2	13	13	13	36.108	0	55833000	4	1.00311	-2.90386	0.202703	-1.16858
Eif2s2	Eukaryotic translation initiation fac	Q99L45;E0CXJ3	Q99L45;E0CXJ3	17.5	17.5	20.2729	17.5	17.5	22.5292	17.5	17.5	19.0203	2	2	2	7.6	7.6	7.6	38.092	0.004357	7851600	1	0.146033	-0.752116	0.147363	0.417521
Eif2s3y;Eif2s3x	Eukaryotic translation initiation fac	Q9Z0N2;Q9Z0N1	Q9Z0N2;Q9Z0N1	17.5	17.5	22.0674	22.694	17.5	20.8507	17.5	17.5	21.5138	3	3	3	11.7	11.7	11.7	51.13	0	18402000	3	0.243287	-1.32578	0.0306607	0.184526
Eif3a	Eukaryotic translation initiation fac	P23116	P23116	21.3824	22.613	22.8739	22.9042	20.9611	22.8029	17.5	20.4162	24.3273	5	5	5	4.2	4.2	4.2	161.93	0	70824000	13	0.0288211	0.0669823	0.309855	1.5419
Eif3b	Eukaryotic translation initiation fac	Q8JZQ9	Q8JZQ9	17.5	17.5	17.5	23.2217	24.2768	24.1109	21.3676	21.0752	25.0223	3	3	3	6.4	6.4	6.4	91.369	0	1.79E+08	6	4.38479	-6.36979	1.76638	-4.98838
Eif3c	Eukaryotic translation initiation fac	Q8R1B4;M0QW	Q8R1B4	23.4689	24.1716	23.7284	24.4733	20.4159	22.9018	17.5	22.4658	23.5536	7	7	7	9	9	9	105.53	0	1.82E+08	23	0.424652	1.19262	0.628464	2.61647
Eif3d	Eukaryotic translation initiation fac	O70194	O70194	17.5	17.5	17.5	17.5	17.5	17.5	17.5	17.5	17.5	1	1	1	4.6	4.6	4.6	63.988	0	0	1	-4.34E-08	0	-4.34E-08	0
Eif3l	Eukaryotic translation initiation fac	Q8QZY1	Q8QZY1	17.5	17.5	17.5	21.0982	17.5	21.9749	20.3968	17.5	17.5	1	1	1	2.8	2.8	2.8	66.612	0.004386	11212000	2	0.917983	-2.69104	0.427243	-0.965586
Eif4a1;Eif4a2	Eukaryotic initiation factor 4A-I;Euk	P60843;P10630	P60843;P10630;Q8BTU6;P	24.0024	25.9397	25.4677	25.452	24.6141	24.8227	17.5	23.7194	26.8507	8	8	8	29.3	29.3	29.3	46.153	0	4.33E+08	29	0.0979327	0.173657	0.363568	2.44656
Eif4b	Eukaryotic translation initiation fac	Q8BGD9	Q8BGD9	21.4504	22.2454	23.6593	22.1482	17.5	17.5	17.5	22.4879	22.3293	1	1	1	2.6	2.6	2.6	68.839	0.004193	51925000	6	0.947833	3.39562	0.403264	1.6726
Eif4g1	Eukaryotic translation initiation fac	Q6NZJ6;A0A0J9	Q6NZJ6;A0A0J9YUS5;E9PV	22.7295	23.4423	22.4435	25.5366	22.4874	25.2902	17.5	24.1822	25.4458	8	8	8	8.1	8.1	8.1	176.07	0	2.26E+08	16	0.698632	-1.56634	0.0698217	0.495742
Eif4h	Eukaryotic translation initiation fac	Q9WUK2;Q9WU	Q9WUK2;Q9WUK2-2	17.5	20.3496	22.4598	22.6603	17.5	22.0283	17.5	23.5975	24.4109	2	2	2	13.3	13.3	13.3	27.341	0	65655000	4	0.103968	-0.626438	0.264994	-1.73301
Eif5a;Eif5a2	Eukaryotic translation initiation fac	P63242;Q8BGY2	P63242;Q8BGY2;A0A0A0M	17.5	20.8665	23.2992	22.642	22.5278	25.5018	17.5	23.0701	24.6195	1	1	1	7.8	7.8	7.8	16.832	0	1.44E+08	11	0.722662	-3.06193	0.170587	-1.23463
Eif5b	Eukaryotic translation initiation fac	Q05D44	Q05D44	20.6825	17.5	17.5	17.5	20.8304	17.5	17.5	17.5	24.1124	2	2	2	3.1	3.1	3.1	137.61	0	96131000	4	0.0105846	-0.0493056	0.177472	-1.14328
Eif6	Eukaryotic translation initiation fac	O55135;A6PWZ	O55135;A6PWZ2	17.5	17.5	17.5	17.5	17.5	17.5	17.5	17.5	22.1585	1	1	1	7.3	7.3	7.3	26.511	0	19079000	2	-4.34E-08	0	0.427243	-1.55283
Elavl1	ELAV-like protein 1	P70372	P70372	19.3871	19.916	20.9303	20.3393	17.5	22.123	17.5	20.6756	21.1576	1	1	1	3.4	3.4	3.4	36.169	0.003824	16438000	4	0.021217	0.0903619	0.0863072	0.300044
Eno1	Alpha-enolase;Enolase	P17182;Q6PHC1	P17182;Q6PHC1;B0QZL1	25.9275	27.3236	26.8219	27.8397	26.8873	27.0115	25.6096	27.6821	28.5355	20	20	20	55.8	55.8	55.8	47.14	0	2.09E+09	103	0.47595	-0.555138	0.240087	-0.584727
Eppk1	Epiplakin	Q8R0W0	Q8R0W0	17.5	17.5	17.5	20.5283	17.5	17.5	17.5	17.5	17.5	1	1	1	2.6	2.6	2.6	723.3	0	5616700	1	0.427243	-1.00944	-4.34E-08	0
Eprs	Bifunctional glutamate/proline--tRN	Q8CGC7	Q8CGC7	21.1198	17.5	17.5	17.5	17.5	24.1039	17.5	22.6469	23.5983	2	2	2	2.6	2.6	2.6	170.08	0	50584000	7	0.147429	-0.994705	0.493522	-2.54183
Erh	Enhancer of rudimentary homolog	P84089	P84089	17.5	21.3157	22.4671	21.853	17.5	19.9313	17.5	17.5	20.9869	1	1	1	10.6	10.6	10.6	12.259	0.003861	17314000	1	0.124374	0.666166	0.392462	1.7653
Esd	S-formylglutathione hydrolase	Q9R0P3;H3BL99	Q9R0P3;H3BL99;H3BJC6;H	21.3018	22.3129	23.1595	23.1494	21.3898	22.3264	17.5	20.6393	24.028	1	1	1	7.8	7.8	7.8	31.319	0	65765000	5	0.0136215	-0.0304521	0.321384	1.53567
Fasn	Fatty acid synthase;[Acyl-carrier-pro	P19096;A0A0U1	P19096;A0A0U1RNJ1	19.8905	24.2225	23.6084	24.3877	17.5	22.8152	20.4328	22.4234	25.1422	9	9	9	4.8	4.8	4.8	272.43	0	1.24E+08	16	0.151053	1.00621	0.0159273	-0.092329
Flna	Filamin-A	Q8BTM8;B7FAU	Q8BTM8;B7FAU9;B7FAV1	26.6317	28.2287	28.187	29.3637	28.4194	29.2673	25.658	27.6939	29.6054	62	62	61	36.7	36.7	36.2	281.22	0	4.68E+09	374	1.03564	-1.33434	0.00786245	0.0300255
Flnb	Filamin-B	Q80X90	Q80X90	22.5119	24.932	24.6428	26.8942	24.9422	27.7568	18.5357	24.3737	27.0584	21	20	20	14.3	13.6	13.6	277.82	0	8.04E+08	68	1.04068	-2.50217	0.0961125	0.706329
Flnc	Filamin-C	Q8VHX6;Q8VHX	Q8VHX6;Q8VHX6-2	23.3215	25.2705	24.4838	26.1303	24.9657	26.4391	22.5646	22.7336	26.7728	26	25	24	13.7	13.2	13.2	291.12	0	5.03E+08	61	0.963751	-1.48643	0.0794276	0.334956
Fscn1	Fascin	Q61553;A0A0G2	Q61553;A0A0G2JDU7	22.2252	21.9395	22.5411	24.4257	22.9586	22.4539	21.8023	22.3845	24.474	4	4	4	12.6	12.6	12.6	54.507	0	1.06E+08	13	0.781142	-1.04412	0.322359	-0.651661
Fxyd5	FXYD domain-containing ion transp	P97808;F6TWM	P97808;F6TWM7;F8WJA1	17.5	24.5493	26.0718	17.5	23.8746	21.9363	17.5	23.6874	22.751	1	1	1	5.1	5.1	5.1	19.454	0.003876	1.41E+08	1	0.188999	1.60341	0.160146	1.39421
Gapdh;Gm7293	Glyceraldehyde-3-phosphate dehyd	P16858;A0A0A0	P16858;A0A0A0MQF6;S4R	25.7838	26.7976	27.9732	27.8606	28.2069	28.0599	25.9109	26.4636	28.4371	15	15	15	44.7	44.7	44.7	35.81	0	2.2E+09	90	0.86485	-1.19092	0.028969	-0.0856539
Gbp2	Interferon-induced guanylate-bindi	Q9Z0E6	Q9Z0E6	17.5	17.5	17.5	17.5	23.7884	23.7547	17.5	22.976	23.0162	2	2	2	3.7	3.7	3.7	66.739	0	77526000	7	0.935095	-4.18102	0.935086	-3.66408
Gdi2	Rab GDP dissociation inhibitor beta	Q61598;Q61598	Q61598;Q61598-2	17.5	21.1648	17.5	22.5013	17.5	17.5	20.0839	17.5	22.0958	2	2	2	5.8	5.8	5.8	50.537	0	20498000	3	0.0757772	-0.44547	0.258168	-1.17163
Gfpt1	Glutamine--fructose-6-phosphate am	P47856;P47856	P47856;P47856-2	17.5	17.5	17.5	21.8415	17.5	17.5	17.5	17.5	20.588	1	1	1	2.3	2.3	2.3	78.538	0	8109200	1	0.427243	-1.44715	0.427243	-1.02934
Glud1	Glutamate dehydrogenase 1, mitocl	P26443;F7CFA5	P26443;F7CFA5	19.8764	17.5	17.5	22.2926	17.5	19.5879	17.5	17.5	21.3281	1	1	1	3.8	3.8	3.8	61.336	0	15591000	2	0.397371	-1.50137	0.117224	-0.483901
Gm5414		Q6IFZ8	Q6IFZ8	23.1169	27.2321	30.319	25.3834	26.8819	24.9814	23.2842	26.8292	24.237	9	2	0	12.1	2.9	0	60.227	0	3.36E+09	50	0.203299	1.14046	0.377832	2.10591
Gnai2;Gnao1;Gnas;Gnai3	Guanine nucleotide-binding protein	P08752;P18872	P08752;P18872;P63094;Q	19.0141	22.2829	17.5	22.7849	17.5	21.3459	17.5	17.5	21.0531	2	2	2	7.3	7.3	7.3	40.489	0.002217	24266000	5	0.16845	-0.944589	0.190008	0.914618
Gnb1	Guanine nucleotide-binding protein	P62874;H3BLF7	P62874;H3BLF7;H3BKR2	17.5	19.3037	17.5	22.8636	17.5	17.5	17.5	17.5	22.789	1	1	1	5.3	5.3	5.3	37.377	0.002237	16381000	1	0.249163	-1.18663	0.246703	-1.16178
Gnb2;Gnb4	Guanine nucleotide-binding protein	P62880;P29387	P62880;P29387;A0A0A6Y	17.5	22.683	17.5	22.5079	17.5	21.0058	17.5	17.5	17.5	1	1	1	4.1	4.1	4.1	37.331	0	23886000	3	0.186145	-1.11022	0.427243	1.72767
Gnb2l1	Guanine nucleotide-binding protein	P68040	P68040	22.4341	23.604	25.2832	24.3391	24.3213	25.319	21.7372	23.9065	25.5201	5	5	5	15.8	15.8	15.8	35.076	0	2.34E+08	32	0.424953	-0.886031	0.0126342	0.0524998
Golgb1		E9QAH1;E9PVZ8	E9QAH1;E9PVZ8	17.5	17.5	28.2228	24.4427	24.2153	22.0766	17.5	24.605	23.2016	1	1	1	0.2	0.2	0.2	365.29	0	5.03E+08	4	0.275139	-2.50391	0.0574158	-0.694609
Got2	Aspartate aminotransferase, mitoch	P05202	P05202	17.5	22.0946	21.0015	22.8813	17.5	20.7848	17.5	17.5	23.076	3	3	3	9.5	9.5	9.5	47.411	0	33854000	9	0.0305963	-0.189993	0.133454	0.840024
Gpi	Glucose-6-phosphate isomerase	P06745;A0A0U1	P06745;A0A0U1RQ18;A0A	19.7169	20.1615	17.5	21.248	17.5	17.5	17.5	17.5	21.0854	2	2	2	7.3	7.3	7.3	62.766	0.002247	19058000	4	0.0896063	0.376799	0.107213	0.431009
Gsdma2;Gsdma	Gasdermin-A2;Gasdermin-A	Q32M21;Q32M2	Q32M21;Q32M21-3;Q9EST	18.8394	17.5	17.5	23.8505	17.5	17.5	17.5	17.5	17.5	1	1	1	3.2	3.2	3.2	49.843	0	15595000	1	0.31593	-1.67038	0.427243	0.446461
Gstp1;Gstp2	Glutathione S-transferase P 1;Glutat	P19157;P46425	P19157;P46425	19.4446	23.8729	20.7794	24.3763	23.1573	23.721	20.9237	17.5	24.3891	3	3	3	22.4	22.4	22.4	23.609	0	1.46E+08	19	0.813143	-2.3859	0.0624092	0.42802
Gtf2f1	General transcription factor IIF subu	Q3THK3	Q3THK3	17.5	17.5	17.5	17.5	17.5	17.5	17.5	17.5	17.5	1	1	1	3.1	3.1	3.1	57.241	0.002242	4333300	2	-4.34E-08	0	-4.34E-08	0
Gvin1	Interferon-induced very large GTPas	Q80SU7;L7N451	Q80SU7;L7N451	17.5	25.2917	26.6442	17.5	21.5167	20.8697	17.5	17.5	23.7292	3	3	3	2.1	2.1	2.1	280.81	0	1.67E+08	3	0.439043	3.18314	0.433344	3.56888
H2-D1	H-2 class I histocompatibility antige	P01899;P01897	P01899	17.5	22.7295	19.1144	23.2516	17.5	17.5	17.5	17.5	23.3727	3	3	3	11.6	11.6	11.6	40.836	0	39040000	6	0.0507939	0.364109	0.0443146	0.32376
Hadha	Trifunctional enzyme subunit alpha	Q8BMS1	Q8BMS1	17.5	20.7166	23.016	17.5	17.5	17.5	17.5	17.5	17.5	2	2	2	3.7	3.7	3.7	82.669	0	10205000	2	0.844876	2.91087	0.844876	2.91087
Hal	Histidine ammonia-lyase	P35492	P35492	17.5	17.5	23.2093	21.3941	17.5	17.5	17.5	17.5	17.5	3	3	3	5.6	5.6	5.6	72.257	0	14622000	3	0.0937763	0.605047	0.427243	1.9031
Hbb-bh1;Hbb-bh0	Hemoglobin subunit beta-H1;Hemo	P04444;P04443	P04444;P04443	19.7038	21.8234	17.5	22.1409	17.5	17.5	23.5689	17.5	17.5	1	1	1	6.8	6.8	6.8	16.494	0.002212	27473000	7	0.114881	0.628771	0.0214354	0.152765
Hdlbp	Vigilin	Q8VDJ3;A0A087	Q8VDJ3;A0A087WP83	17.5	23.1925	22.2952	23.0919	21.7292	24.381	17.5	17.5	23.4979	6	6	6	7.2	7.2	7.2	141.74	0	70369000	12	0.465171	-2.07147	0.218385	1.4966
Hint1	Histidine triad nucleotide-binding p	P70349;B0R1E3	P70349;B0R1E3	20.1647	21.5445	22.7793	22.1251	17.5	17.5	20.8907	22.9389	22.065	1	1	1	11.1	11.1	11.1	13.777	0.004141	35000000	6	0.645875	2.45449	0.186357	-0.468678
Hist1h1a	Histone H1.1	P43275	P43275	24.031	17.5	21.755	17.5	23.4068	22.5551	17.5	23.194	23.1767	5	3	3	25.4	17.4	17.4	21.785	0	83800000	11	0.00724464	-0.0586205	0.0242039	-0.194885
Hist1h1b	Histone H1.5	P43276	P43276	25.4909	25.1469	26.3634	25.6831	23.7143	24.3059	23.2211	25.0772	25.6108	9	7	7	32.7	24.2	24.2	22.576	0	5.91E+08	63	0.733905	1.09931	0.56545	1.03067
Hist1h1c	Histone H1.2	P15864	P15864	17.5	17.5	17.5	17.5	22.6367	22.7035	17.5	22.6714	23.0877	9	1	1	33	9.4	9.4	21.266	0	46639000	2	0.935044	-3.44673	0.932885	-3.58637
Hist1h1d	Histone H1.3	P43277;Q07133	P43277	26.1797	24.0432	24.4691	25.366	25.0924	25.102	24.5709	25.2231	24.6425	12	5	3	35.3	15.4	9.5	22.099	0	5.07E+08	34	0.165484	-0.289482	0.0423848	0.0851676
Hist1h1e	Histone H1.4	P43274	P43274	28.1389	24.9628	28.1234	27.386	26.9605	25.8711	25.7186	27.219	27.2672	13	13	6	39.7	39.7	19.6	21.977	0	1.88E+09	104	0.105411	0.335847	0.104513	0.340097
Hist1h2ab;Hist1h2af;Hist	Histone H2A type 1;Histone H2A typ	P22752;Q8CGP	P22752;Q8CGP5;Q8CGP7;(	24.9918	26.0179	25.372	26.2955	26.7121	25.9464	24.0499	25.983	27.6475	5	5	2	20.8	20.8	13.8	14.135	0	1.11E+09	77	1.08246	-0.857459	0.149074	-0.432938
Hist1h2bf;Hist1h2bc;His	Histone H2B type 1-F/J/L;Histone H2	P10853;Q6ZWY	P10853;Q6ZWY9;Q64478;(	26.4721	27.0743	27.3903	28.4595	27.7428	28.2433	26.0392	26.7188	28.9241	7	7	7	43.7	43.7	43.7	13.936	0	2.68E+09	95	1.56867	-1.16966	0.0976444	-0.24847
Hist1h3a;H3f3a;Hist1h3t	Histone H3.1;Histone H3.3;Histone	P68433;P84244	P68433;P84244;P84228;P	25.4892	27.6915	28.8262	27.8563	26.6077	27.3501	26.1522	26.6929	27.2349	4	4	4	19.9	19.9	19.9	15.404	0	2.1E+09	53	0.0205042	0.0642764	0.247139	0.6423
Hist1h4a	Histone H4	P62806	P62806	25.2289	27.505	28.4075	27.5299	26.4866	27.0458	26.0751	27.7949	28.6227	8	8	8	56.3	56.3	56.3	11.367	0	1.97E+09	68	0.00872628	0.0263303	0.137882	-0.450446
Hmga1	High mobility group protein HMG-I/	P17095-1	P17095-1	17.5	17.5	26.127	24.6961	17.5	19.9751	22.7026	24.9752	24.566	1	1	1	14.6	14.6	14.6	10.617	0	3.66E+08	9	0.0329356	-0.348051	0.554787	-3.70559
Hmga1	High mobility group protein HMG-I/	P17095	P17095	17.5	23.8987	24.8206	21.8967	17.5	23.8385	17.5	25.1525	24.5089	2	2	2	23.4	23.4	23.4	11.614	0	2.42E+08	19	0.122384	0.994721	0.0314799	-0.314021
Hmga2	High mobility group protein HMGI-(	P52927;Q6NSP9	P52927;Q6NSP9	23.3766	24.1796	17.5	23.5232	17.5	23.7888	17.5	17.5	17.5	1	1	1	21.3	21.3	21.3	11.819	0	1.01E+08	5	0.00910855	0.0814006	0.929074	4.1854
Hmgb1	High mobility group protein B1	P63158;A0A0J9	P63158;A0A0J9YUZ4;A0A0	24.1498	21.4067	24.9599	25.7615	20.8936	25.7182	17.5	24.4418	25.9182	9	9	9	39.1	39.1	39.1	24.893	0	3.62E+08	28	0.11594	-0.61987	0.114431	0.885441
Hmgn1	Non-histone chromosomal protein I	P18608;D3Z607	P18608;D3Z607	17.5	17.5	17.5	17.5	17.5	17.5	17.5	22.3096	22.9596	2	2	2	36.5	36.5	36.5	10.152	0.005629	33166000	2	-4.34E-08	0	0.929195	-3.42304
Hmox2	Heme oxygenase 2	O70252;D3YX62	O70252;D3YX62	17.5	17.5	17.5	22.2743	17.5	24.062	17.5	17.5	22.437	2	2	2	14.3	14.3	14.3	35.738	0	28265000	3	0.899928	-3.77876	0.427243	-1.64567
Hn1l	Hematological and neurological exp	Q6PGH2	Q6PGH2	17.5	17.5	17.5	17.5	17.5	21.5935	17.5	17.5	21.4146	2	2	2	18.9	18.9	18.9	20.02	0.003831	9080200	2	0.427243	-1.36448	0.427243	-1.30486
Hnrnpa1	Heterogeneous nuclear ribonucleop	P49312;Q5EBP8	P49312;Q5EBP8;P49312-2	22.5358	22.7415	23.9932	23.1697	23.4925	25.6104	17.5	24.8226	25.7883	5	5	5	24.1	24.1	24.1	34.196	0	2.42E+08	20	0.489358	-1.00073	0.0499556	0.386497
Hnrnpa2b1	Heterogeneous nuclear ribonucleop	O88569;O88569	O88569;O88569-3;O88569	23.1267	23.6952	23.8139	25.1415	22.9571	23.1689	22.0803	23.599	22.9569	2	2	2	9.9	9.9	9.9	37.402	0	2.13E+08	7	0.104273	-0.210536	0.612246	0.666547
Hnrnpa3;Gm6793;Gm92	Heterogeneous nuclear ribonucleop	Q8BG05;A2AL12	Q8BG05;A2AL12;B2RXM2;(	22.4592	24.1434	23.2653	24.4145	22.571	26.4241	21.1171	23.9072	25.186	7	7	7	29.6	29.6	29.6	39.652	0	2.56E+08	24	0.413412	-1.18056	0.0296252	-0.114128
Hnrnpab	Heterogeneous nuclear ribonucleop	Q99020;Q80XR6	Q99020;Q80XR6;Q20BD0	24.0161	17.5	17.5	17.5	17.5	23.6033	17.5	23.7532	22.8968	1	1	1	4.6	4.6	4.6	30.831	0	59844000	5	0.0153184	0.137581	0.22931	-1.71129
Hnrnpc	Heterogeneous nuclear ribonucleop	Q9Z204;Q9Z204	Q9Z204;Q9Z204-2;Q9Z204	22.9506	20.7856	17.5	22.0726	22.1843	22.949	22.0809	17.5	23.2864	2	2	2	7.3	7.3	7.3	34.384	0	43514000	2	0.547226	-1.98992	0.0810248	-0.543725
Hnrnpd	Heterogeneous nuclear ribonucleop	Q60668;Q60668	Q60668;Q60668-3;E9Q5B6	21.5602	19.5708	20.7189	19.9837	22.2451	23.327	19.0905	22.2353	23.8773	2	2	2	6.8	6.8	6.8	38.354	0	51452000	5	0.46854	-1.23529	0.298938	-1.11772
Hnrnph1;Hnrnph2	Heterogeneous nuclear ribonucleop	O35737;P70333	O35737;P70333;Q8C2Q7	21.4422	22.5852	22.2243	23.3843	20.9827	23.862	17.5	23.0969	23.9	2	2	1	6	6	3.8	49.199	0	87402000	9	0.278136	-0.659111	0.103111	0.584919
Hnrnpk	Heterogeneous nuclear ribonucleop	P61979;P61979	P61979;P61979-2;P61979	21.6338	24.9304	23.7994	24.8141	24.5025	25.3884	23.265	23.8474	25.0711	5	5	5	18.8	18.8	18.8	50.976	0	2.84E+08	13	0.653826	-1.44716	0.213342	-0.606649
Hnrnpl	Heterogeneous nuclear ribonucleop	Q8R081;G3UY3	Q8R081;G3UY38;G5E924	17.5	17.5	17.5	21.2515	17.5	21.2873	17.5	17.5	23.0566	2	2	2	6.8	6.8	6.8	63.963	0.005597	13780000	2	0.935072	-2.51295	0.427243	-1.85221
Hnrnpm	Heterogeneous nuclear ribonucleop	Q9D0E1;B8JK33	Q9D0E1;B8JK33;B8JK32;Q	17.5	21.8351	17.5	17.5	17.5	17.5	17.5	17.5	17.5	1	1	1	2.2	2.2	2.2	77.648	0	4977300	2	0.427243	1.44504	0.427243	1.44504
Hnrnpu	Heterogeneous nuclear ribonucleop	Q8VEK3;G3XA1C	Q8VEK3;G3XA10	24.5906	26.192	25.6409	26.8409	24.947	26.5523	23.3688	26.3912	27.4666	12	12	12	21.5	21.5	21.5	87.917	0	9.16E+08	65	0.352417	-0.6389	0.0713712	-0.267694
Hp1bp3	Heterochromatin protein 1-binding	Q3TEA8;Q3TEA8	Q3TEA8;Q3TEA8-2;A2AM70	17.5	17.5	17.5	17.5	17.5	22.9296	17.5	17.5	17.5	1	1	1	2.7	2.7	2.7	60.866	0.004082	16138000	1	0.427243	-1.80987	-4.34E-08	0
Hsp90aa1	Heat shock protein HSP 90-alpha	P07901;B7ZC50	P07901	21.7972	23.3104	25.1185	26.0739	24.1511	23.6565	21.74	25.562	27.0738	21	12	12	28.4	15.8	15.8	84.787	0	4.8E+08	39	0.430536	-1.2185	0.303392	-1.38321
Hsp90ab1	Heat shock protein HSP 90-beta	P11499;E9Q3D6	P11499	26.0252	28.418	28.1027	28.3944	27.5518	27.3005	25.271	27.8319	28.8824	29	29	18	38.1	38.1	23.5	83.28	0	2.95E+09	202	0.102409	-0.233607	0.0489695	0.186904
Hsp90b1	Endoplasmin	P08113;F7C312	P08113	22.634	24.1472	22.6651	25.2721	22.7752	23.5444	21.8936	23.1414	25.7842	10	8	8	17.7	16	16	92.475	0	2.07E+08	20	0.330382	-0.715092	0.134889	-0.457625
Hspa1a;Hspa1b	Heat shock 70 kDa protein 1A;Heat	Q61696;P17879	Q61696;P17879	19.8875	22.4017	21.3271	22.3649	17.5	17.5	17.5	17.5	21.912	6	1	1	11.4	2.5	2.5	70.078	0	21380000	2	0.514044	2.08381	0.610937	2.23476
Hspa4	Heat shock 70 kDa protein 4	Q61316;Q3U2G	Q61316;Q3U2G2	17.5	21.4754	20.1866	23.1661	17.5	21.487	17.5	20.9495	23.4124	5	5	5	8.4	8.4	8.4	94.132	0	33977000	6	0.185838	-0.996997	0.163003	-0.899928
Hspa5	78 kDa glucose-regulated protein	P20029	P20029	24.7845	26.6694	26.3653	26.9578	24.9728	25.2307	22.1575	25.458	27.2263	23	21	21	39.5	39.5	39.5	72.421	0	8.62E+08	101	0.0914885	0.219306	0.245837	0.992481
Hspa8	Heat shock cognate 71 kDa protein	P63017;Q504P4	P63017;Q504P4	27.093	28.5856	29.1891	29.0505	28.5104	28.1061	26.5337	28.2201	29.3686	25	25	19	43.7	43.7	34.8	70.87	0	4.84E+09	289	0.145529	-0.266492	0.0852949	0.248409
Hspa9	Stress-70 protein, mitochondrial	P38647	P38647	17.5	23.8513	21.1677	24.9879	23.463	25.6821	20.2048	23.7465	25.333	8	8	8	18	18	18	73.46	0	2.49E+08	24	0.925809	-3.87134	0.400285	-2.25512
Hspd1	60 kDa heat shock protein, mitocho	P63038;P63038	P63038	22.1182	23.527	23.6528	24.5525	23.8975	23.9406	20.7784	22.284	25.3531	7	7	7	19.4	19.4	19.4	60.955	0	1.69E+08	26	0.897916	-1.0309	0.0719165	0.294163
Hspe1	10 kDa heat shock protein, mitocho	Q64433	Q64433	17.5	17.5	17.5	17.5	17.5	17.5	17.5	21.5862	21.1643	1	1	1	13.7	13.7	13.7	10.963	0.005566	10621000	3	-4.34E-08	0	0.930725	-2.58349
Ifi205a	Interferon-activable protein 205-A	P0DOV1-2;P0DC	P0DOV1-2;P0DOV1;Q8CGE	17.5	17.5	22.7512	21.697	17.5	21.9614	17.5	22.7823	23.514	2	2	2	6.9	6.9	6.9	46.776	0	54919000	3	0.191722	-1.13573	0.320275	-2.01505
Ifitm3	Interferon-induced transmembrane	Q9CQW9;Q99J9	Q9CQW9	20.1981	23.8314	25.9768	24.6009	23.4294	21.0882	17.5	17.5	22.5664	4	4	4	48.2	48.2	48.2	14.954	0	1.41E+08	6	0.0514485	0.295935	0.803222	4.14664
Igtp		Q9DCE9;Q9Z1M	Q9DCE9	22.295	25.1694	25.0665	25.1027	23.6671	23.3515	20.2328	22.7526	25.4351	7	7	7	22.7	22.7	22.7	48.493	0	2.34E+08	21	0.0428997	0.136492	0.316305	1.37009

Iigp1	Interferon-inducible GTPase 1	Q9QZ85;Q3UED	Q9QZ85	21.5097	24.4122	24.5503	25.3955	24.3218	25.8122	21.4445	23.5403	25.4777	7	7	7	24	24	24	47.571	0	2.88E+08	40	0.70859	-1.68572	0.000696686	0.00326856
Impdh2	Inosine-5-monophosphate dehydro	P24547;A0A0A6	P24547;A0A0A6YY72	17.5	19.7902	17.5	22.4061	17.5	22.0942	17.5	17.5	23.0973	3	3	3	7.2	7.2	7.2	55.814	0	25015000	4	0.612878	-2.4034	0.212142	-1.10238
Ipo5	Importin-5	Q8BKC5;Q8BKC5	Q8BKC5;Q8BKC5-2	17.5	17.5	17.5	17.5	17.5	17.5	17.5	17.5	17.5	1	1	1	1.5	1.5	1.5	123.59	0	0	1	-4.34E-08	0	-4.34E-08	0
Ipo9	Importin-9	Q91YE6;E0CY46;	Q91YE6;E0CY46;E0CXB2;E9	17.5	17.5	22.3533	17.5	17.5	21.9534	17.5	17.5	17.5	1	1	1	2	2	2	116.05	0	9419100	1	0.0202228	0.133296	0.427243	1.61776
Iqgap1	Ras GTPase-activating-like protein IQ	Q9JKF1	Q9JKF1	20.5814	19.068	17.5	17.5	17.5	23.8657	17.5	17.5	20.2893	2	2	2	2.1	2.1	2.1	188.74	0	23923000	3	0.0883769	-0.572108	0.183698	0.620031
Irgm1	Immunity-related GTPase family M p	Q60766;Q5NCB5	Q60766;Q5NCB5;Q60766-2	17.5	23.2947	23.4162	21.3827	22.2536	23.6146	17.5	21.6918	19.8899	3	3	3	9	9	9	46.551	0	53510000	11	0.188321	-1.0133	0.30247	1.70976
Itgb1	Integrin beta-1	P09055;P09055	P09055;P09055-2	21.4053	21.8273	22.0607	22.5349	17.5	22.9887	17.5	17.5	22.6231	2	2	2	4.6	4.6	4.6	88.231	0	38131000	1	0.16055	0.756535	0.675684	2.55671
Jup	Junction plakoglobin	Q02257;Q0224	Q02257	29.0322	30.0735	32.2	28.4417	28.9022	26.8946	26.5041	29.4443	27.8458	29	29	29	42.4	42.4	42.4	81.8	0	1.13E+10	344	0.993023	2.35578	0.927632	2.50387
Kars	Lysine--tRNA ligase	Q99MN1	Q99MN1	20.2145	21.6328	22.0436	21.7172	22.3414	21.9408	21.0366	21.4975	21.7043	1	1	1	2.5	2.5	2.5	67.839	0	39163000	7	0.530649	-0.702847	0.0687957	-0.11581
Khsrp	Far upstream element-binding prote	Q3U0V1	Q3U0V1	22.2914	23.8471	24.107	22.606	23.0649	24.3949	20.7698	22.8619	24.4717	6	6	6	11	11	11	76.775	0	1.45E+08	11	0.0257168	0.0599028	0.230967	0.714048
Kpna2	Importin subunit alpha-1	P52293;A2A600	P52293;A2A600;A2A601;A	20.4673	20.6091	21.1643	20.292	22.0659	22.6744	17.5	17.5	21.951	3	3	3	8.3	8.3	8.3	57.927	0	24881000	4	0.552683	-0.930511	0.516186	1.76324
Kpnb1	Importin subunit beta-1	P70168	P70168	21.149	22.8993	21.3526	24.2172	22.8318	23.6622	19.8216	22.951	24.0688	4	4	4	6.1	6.1	6.1	97.183	0	96696000	20	1.21641	-1.77005	0.126978	-0.480134
Krt27	Keratin, type I cytoskeletal 27	Q9Z320	Q9Z320	17.5	20.6878	17.5	22.1141	17.5	17.5	17.5	17.5	17.5	2	1	1	4	2	2	49.104	1	6228700	1	0.0905591	-0.475437	0.427243	1.06261
Krt76	Keratin, type II cytoskeletal 2 oral	Q3UV17	Q3UV17	31.0281	31.5905	32.5898	30.7475	31.4087	29.5952	23.1806	31.1945	30.0894	17	2	2	14.6	1.9	1.9	62.844	0	2.66E+10	99	0.757399	1.15234	0.633224	3.58131
Lap3	Cytosol aminopeptidase	Q9CPY7;Q9CPY7	Q9CPY7;Q9CPY7-2	17.5	17.5	17.5	21.7162	17.5	21.6057	17.5	17.5	21.8994	1	1	1	2.3	2.3	2.3	56.141	0.004264	10942000	1	0.934844	-2.77398	0.427243	-1.46645
Lasp1	LIM and SH3 domain protein 1	Q61792;A2A6G9	Q61792;A2A6G9;A2A6H0;A	25.3847	22.8213	22.923	22.9467	17.5	17.5	17.5	21.5556	24.6293	6	6	6	27	27	27	29.994	0	1.59E+08	15	1.03191	4.39412	0.484348	2.48138
Ldha	L-lactate dehydrogenase A chain;L-la	P06151;A0A1B0	P06151;A0A1B0GSR9;Q564	24.0309	25.397	25.2958	26.4855	25.7034	24.8878	25.9082	24.7444	26.4599	9	9	8	27.1	27.1	23.5	36.498	0	6.06E+08	39	0.544079	-0.784376	0.522398	-0.796291
Lgals1	Galectin-1	P16045	P16045	20.0773	22.6776	22.5932	22.0898	17.5	23.3521	17.5	17.5	23.3823	1	1	1	13.3	13.3	13.3	14.866	0	55997000	5	0.151791	0.8021	0.470323	2.32195
Lgals3	Galectin-3	P16110;Q8C253	P16110;Q8C253	17.5	21.8967	23.4251	21.809	17.5	17.5	17.5	21.7013	21.4189	1	1	1	4.2	4.2	4.2	27.515	0.004073	33712000	4	0.36677	2.00428	0.119733	0.733878
Lmna	Prelamin-A/C;Lamin-A/C	P48678;P48678	P48678;P48678-2;P48678	25.9729	26.0056	26.5895	26.043	25.7161	24.4837	17.5	26.3877	26.37	19	19	19	34.7	34.7	34.7	74.237	0	7.36E+08	84	0.684083	0.775075	0.394423	2.77012
Lrrc59	Leucine-rich repeat-containing prot	Q922Q8	Q922Q8	17.5	21.4683	25.8321	23.5401	20.1431	19.983	17.5	19.3896	20.9087	3	3	3	12.4	12.4	12.4	34.877	0.00232	84598000	5	0.0485234	0.378077	0.376606	2.33408
Lrrfip1	Leucine-rich repeat flightless-intera	Q3UZ39;Q3UZ39	Q3UZ39;Q3UZ39-3	17.5	17.5	17.5	21.1971	17.5	17.5	17.5	21.0975	21.7953	1	1	1	4.3	4.3	4.3	79.248	0.004167	8287500	3	0.427243	-1.23238	0.923665	-2.63094
Luc7l2	Putative RNA-binding protein Luc7-	Q7TNC4;E9Q715	Q7TNC4;E9Q715;Q7TNC4-2	21.1396	21.8737	22.6307	23.3597	21.1051	21.7887	17.5	21.9077	24.6604	5	5	5	15.6	15.6	15.6	46.582	0	1.56E+08	11	0.0911331	-0.203171	0.087628	0.525305
Luc7l3	Luc7-like protein 3	Q5SUF2;Q5SUF2	Q5SUF2;Q5SUF2-3;Q5SUF2	20.6796	17.5	23.5385	23.631	17.5	20.4683	17.5	22.1119	23.2448	5	5	5	13	13	13	51.45	0	93135000	15	0.00521978	0.0395857	0.0527778	-0.379541
Lyz1;Lyz2	Lysozyme C-1;Lysozyme C-2	P17897;P08905	P17897;P08905	22.1381	23.933	25.1281	24.0457	17.5	17.5	20.3862	24.2608	24.6291	2	2	2	14.2	14.2	14.2	16.794	0	1.79E+08	23	0.796912	4.05117	0.148071	0.641027
Map4	Microtubule-associated protein 4;M	P27546;P27546	P27546;P27546-4;P27546	17.5	17.5	17.5	21.4869	17.5	17.5	17.5	20.9	21.7402	2	2	2	2.3	2.3	2.3	117.43	0.004132	17152000	2	0.427243	-1.32898	0.917558	-2.54672
Marcks	Myristoylated alanine-rich C-kinase	P26645	P26645	17.5	17.5	17.5	17.5	17.5	17.5	17.5	23.3196	23.8909	3	3	3	28.5	28.5	28.5	29.661	0	1.37E+08	10	-4.34E-08	0	0.931865	-4.07016
Marcksl1	MARCKS-related protein	P28667	P28667	21.8292	20.6345	17.5	22.1318	17.5	21.8092	20.2397	22.784	23.0141	2	2	2	27.5	27.5	27.5	20.165	0	65424000	9	0.0887011	-0.492443	0.575096	-2.02469
Mars	Methionine--tRNA ligase, cytoplasm	Q68FL6;E9QB02	Q68FL6;E9QB02	17.5	17.5	17.5	21.721	17.5	21.7606	17.5	17.5	20.6558	3	3	3	5.1	5.1	5.1	101.43	0	9630800	3	0.935074	-2.82722	0.427243	-1.05193
Mat2a	S-adenosylmethionine synthase isof	Q3THS6;A0A0U1	Q3THS6;A0A0U1RNT6;A0A(	17.5	17.5	21.1927	17.5	17.5	17.5	17.5	21.3707	22.455	2	2	2	7.6	7.6	7.6	43.688	0	13216000	4	0.427243	1.23089	0.368201	-1.711
Matr3	Matrin-3	Q8K310;A0A087	Q8K310;A0A087WSP7;A0A	17.5	17.5	17.5	21.1722	21.2393	20.675	17.5	20.9298	22.5349	2	2	2	3.9	3.9	3.9	94.629	0.00566	14587000	3	4.41828	-3.52888	0.885332	-2.82158
Mccc1	Methylcrotonoyl-CoA carboxylase s	Q99MR8;A0A0G	Q99MR8	18.833	28.3745	28.9881	27.6604	28.3045	28.0596	23.3378	18.7549	23.2083	27	27	27	45.9	45.9	45.9	79.343	0	1.76E+09	108	0.325674	-2.60965	0.429345	3.63153
Mcm3	DNA replication licensing factor MC	P25206	P25206	17.5	17.5	20.7799	17.5	17.5	21.2638	17.5	17.5	21.7682	2	2	2	3.9	3.9	3.9	91.545	0	7890200	3	0.0327153	-0.16132	0.0638616	-0.329444
Mcm4	DNA replication licensing factor MC	P49717	P49717	17.5	17.5	17.5	17.5	17.5	17.5	17.5	17.5	22.5388	1	1	1	3.1	3.1	3.1	96.735	0.003884	6093300	1	-4.34E-08	0	0.427243	-1.6796
Mdh2	Malate dehydrogenase, mitochondr	P08249;A0A0G2	P08249	22.0254	23.2411	24.7587	25.5899	21.6423	25.2982	23.7261	22.6858	26.0941	5	5	5	20.4	20.4	20.4	35.611	0	2.68E+08	17	0.217181	-0.835065	0.256589	-0.826939
Mif	Macrophage migration inhibitory fa	P34884	P34884	21.1735	18.7858	23.3108	23.6469	17.5	21.8997	20.2435	24.1166	23.1731	2	2	2	15.7	15.7	15.7	12.504	0	77553000	9	0.0109314	0.0745099	0.334719	-1.42106
Msn	Moesin	P26041;P26043	P26041	23.8322	24.4018	24.436	25.8015	23.3901	25.6917	18.194	21.7089	27.3593	19	19	19	31.7	31.7	31.7	67.766	0	4.61E+08	40	0.382987	-0.737747	0.269546	1.80259
Mtap	S-methyl-5-thioadenosine phospho	Q9CQ65	Q9CQ65	17.5	18.5334	20.2169	17.5	20.6958	17.5	17.5	17.5	22.2026	2	2	2	12.4	12.4	12.4	31.062	0	12803000	2	0.0477046	0.184839	0.0628064	-0.31742
Mthfd1	C-1-tetrahydrofolate synthase, cyto	Q922D8	Q922D8	17.5	20.6006	17.5	21.4243	21.0627	21.8768	17.5	17.5	19.9685	3	3	3	3.6	3.6	3.6	101.2	0	13226000	2	1.29178	-2.92107	0.0550223	0.210712
Mtpn	Myotrophin	P62774	P62774	21.7067	22.232	22.83	22.9199	17.5	17.5	17.5	20.9865	23.8841	2	2	2	30.5	30.5	30.5	12.861	0	72837000	6	0.73672	2.94957	0.320819	1.46601
Myadm	Myeloid-associated differentiation r	O35682;A0A0N4	O35682;A0A0N4SW94	20.9968	23.0301	23.3123	22.9685	22.9009	22.8072	20.6113	21.7917	23.0217	1	1	1	5.3	5.3	5.3	35.284	0	72543000	16	0.240464	-0.445811	0.250976	0.638179
Mybbp1a	Myb-binding protein 1A	Q7TPV4	Q7TPV4	21.6624	23.4092	24.202	24.1956	24.1754	26.0026	22.4072	23.7273	23.9895	12	12	12	10.6	10.6	10.6	152.04	0	2.28E+08	40	0.816263	-1.69997	0.11493	-0.283483
Myh14	Myosin-14	Q6URW6;Q6UR	Q6URW6;Q6URW6-2;K3W	17.5	24.7849	25.7594	24.5671	17.5	24.0888	21.5192	23.0347	24.4284	4	1	1	1.6	0.6	0.6	228.58	0.004329	1.96E+08	14	0.0631916	0.629488	0.0387737	-0.312652
Myh7;Myh6;Myh4;Myh1	Myosin-7;Myosin-6;Myosin-4;Myosi	Q91Z83;Q02566	Q91Z83;Q02566;Q5SX39;C	17.5	17.5	17.5	17.5	17.5	17.5	26.0081	17.5	17.5	5	5	5	2.9	2.9	2.9	222.88	0	67489000	15	-4.34E-08	0	0.427243	-2.83605
Myh9	Myosin-9	Q8VDD5;Q6187	Q8VDD5	28.3289	29.0581	29.4617	30.2019	29.1249	30.0066	27.1792	28.8156	30.2642	87	87	84	46.9	46.9	45.9	226.37	0	9.64E+09	479	0.818397	-0.828248	0.0724693	0.196557
Myl12b;Myl12a	Myosin regulatory light chain 12B	Q3THE2;Q6ZWQ	Q3THE2;Q6ZWQ9	21.3127	17.5	23.6689	22.3964	22.2205	23.8985	17.5	22.6922	25.707	3	3	3	23.8	23.8	23.8	19.779	0	1.65E+08	13	0.46381	-2.01125	0.140811	-1.13918
Myl6	Myosin light polypeptide 6	Q60605;Q60605	Q60605;Q60605-2	17.5	20.4366	19.3252	24.1231	17.5	17.5	17.5	22.3967	24.8267	3	3	3	24.5	24.5	24.5	16.93	0	1.1E+08	8	0.093532	-0.620426	0.463716	-2.48716
Myo1c	Unconventional myosin-Ic	Q9WTI7;Q9WTI7	Q9WTI7;Q9WTI7-3;Q9WTI7	17.5	17.5	19.3022	23.1639	17.5	21.314	17.5	23.941	23.5925	2	2	2	3	3	3	121.94	0	41405000	3	0.652977	-2.5586	0.755654	-3.57712
Naca	Nascent polypeptide-associated con	P70670;Q60817	P70670;Q60817	23.2835	23.9074	24.6986	23.7188	21.9809	23.7149	22.1495	23.4399	25.3705	3	3	3	1.9	1.9	1.9	220.5	0	2.05E+08	22	0.509752	0.824968	0.109722	0.309897
Nap1l1	Nucleosome assembly protein 1-like	P28656;E9PW66	P28656;E9PW66	17.5	21.8018	23.1801	22.8721	17.5	22.6077	17.5	23.6054	24.6947	1	1	1	4.3	4.3	4.3	45.345	0	87303000	4	0.0226611	-0.165964	0.145896	-1.10604
Nccrp1	F-box only protein 50	G3X9C2;A0A0A0	G3X9C2;A0A0A0MQ97	23.3092	22.6145	24.2798	17.5	17.5	23.3065	17.5	17.5	17.5	1	1	1	4.5	4.5	4.5	30.407	0	51201000	1	0.929141	3.96568	3.58919	5.90117
Ncl	Nucleolin	P09405;A0A087	P09405	27.535	27.3049	28.2522	28.5223	27.0057	26.9546	25.9566	28.5416	28.3054	29	29	29	37.5	37.5	37.5	76.722	0	5.03E+09	285	0.126583	0.20315	0.0373438	0.0961533
Nes	Nestin	Q6P5H2;Q6P5H	Q6P5H2;Q6P5H2-2	17.5	17.5	17.5	22.1223	17.5	17.5	17.5	17.5	22.4566	2	2	2	2	2	2	207.12	0	21382000	3	0.427243	-1.54077	0.427243	-1.6522
Nkap	NF-kappa-B-activating protein	Q9D0F4	Q9D0F4	17.5	17.5	17.5	17.5	17.5	17.5	17.5	17.5	17.5	1	1	1	3.9	3.9	3.9	47.227	0.004032	4412300	1	-4.34E-08	0	-4.34E-08	0
Nme1	Nucleoside diphosphate kinase A	P15532;Q5NC8	P15532;Q5NC80	17.5	17.5	17.5	22.2557	17.5	17.5	17.5	21.2202	22.0832	3	1	1	25.7	6.6	6.6	17.208	1	29752000	4	0.427243	-1.58523	0.91939	-2.76778
Nme2;Gm20390	Nucleoside diphosphate kinase B;Nu	Q01768;E9PZF0	Q01768;E9PZF0	21.9847	24.1831	25.258	25.322	22.2844	22.0654	20.8817	24.6351	23.2912	3	3	1	28.3	28.3	9.2	17.363	0	1.82E+08	15	0.153221	0.584712	0.234786	0.872643
Nmt1	Glycylpeptide N-tetradecanoyltrans	O70310	O70310	17.5	17.5	17.5	17.5	17.5	21.2223	17.5	17.5	21.7296	1	1	1	2.6	2.6	2.6	56.888	0.009208	7162800	1	0.427243	-1.24078	0.427243	-1.40986
Nolc1		E9Q5C9	E9Q5C9	25.2166	24.8451	25.9008	25.5638	23.6715	24.3433	22.3345	26.0538	26.827	16	16	16	17.1	17.1	17.1	73.697	0	1.12E+09	81	0.555123	0.794631	0.0608119	0.249106
Nop58	Nucleolar protein 58	Q6DFW4;A0A08	Q6DFW4;A0A087WQ46;A0	17.5	21.1757	17.5	17.5	17.5	22.341	17.5	17.5	17.5	2	2	2	5.4	5.4	5.4	60.342	0	13442000	3	0.0668609	-0.388412	0.427243	1.22524
Npm1;Gm5611	Nucleophosmin	Q61937;Q5SQB	Q61937;Q5SQB0;Q9DAY9;(	26.2391	26.7166	25.5455	26.7091	26.9317	26.7672	24.6101	26.1239	27.2841	12	12	12	35.3	35.3	35.3	32.56	0	1.22E+09	50	0.852416	-0.635634	0.0663787	0.160991
Npm3	Nucleoplasmin-3	Q9CPP0	Q9CPP0	22.9421	23.7367	17.5	17.5	17.5	17.5	17.5	17.5	24.4199	1	1	1	8.6	8.6	8.6	19.023	0.003976	70429000	2	0.928286	3.89291	0.20209	1.58628
Ntf3	Neurotrophin-3	P20181;Q3V1A4	P20181;Q3V1A4	17.5	22.1313	17.5	17.5	22.1781	21.595	21.7756	17.5	21.5145	1	1	1	4.7	4.7	4.7	29.587	0.003802	22891000	2	0.257487	-1.38059	0.230653	-1.21958
Nucks1	Nuclear ubiquitous casein and cycli	Q80XU3;A0A087	Q80XU3;A0A087WRY3	17.5	21.9441	17.5	21.8464	21.5603	20.2351	17.5	22.2312	22.7218	2	2	2	16.2	16.2	16.2	26.313	0.002232	75495000	2	0.645512	-2.23258	0.340817	-1.83631
P4hb	Protein disulfide-isomerase	P09103	P09103	21.3894	23.895	17.5	23.8498	23.3467	21.7043	17.5	21.6289	24.1408	2	2	2	4.9	4.9	4.9	57.058	0	82131000	8	0.444691	-2.03875	0.0200649	-0.161756
Pa2g4	Proliferation-associated protein 2G	P50580;P50580	P50580;P50580-2;D3YVH7	21.0963	23.781	23.6399	23.5443	20.5733	22.7646	18.075	20.076	24.8334	6	6	6	18.3	18.3	18.3	43.698	0	85656000	9	0.164717	0.54497	0.35032	1.84421
Pabpc1;Pabpc6	Polyadenylate-binding protein 1	P29341;Q9D4E6	P29341;Q9D4E6	21.5086	24.086	23.5716	24.4407	22.7851	22.3438	20.5402	21.6344	25.1605	8	8	8	14.8	14.8	14.8	70.67	0	1.28E+08	18	0.0453268	-0.134445	0.141218	0.610399
Paics	Multifunctional protein ADE2;Phos	Q9DCL9;D3Z6P1	Q9DCL9;D3Z6P1;D6RCU8	17.5	22.2879	17.5	17.5	22.3216	22.8807	17.5	17.5	17.5	3	3	3	10.4	10.4	10.4	47.006	0	27701000	4	0.315919	-1.80482	0.427243	1.59595
Pc;Pcx	Pyruvate carboxylase, mitochondria	Q05920;G5E8R3	Q05920;G5E8R3;E9QPD7	19.4329	28.3601	28.3717	27.7507	28.3798	28.7951	23.1014	17.5	24.1328	29	29	29	29.5	29.5	29.5	129.68	0	1.81E+09	102	0.415136	-2.92029	0.453368	3.81017
Pcbp1	Poly(rC)-binding protein 1	P60335	P60335	20.5222	22.6267	22.1223	22.7792	20.9382	23.9856	17.5	18.9492	24.2061	4	4	2	16.3	16.3	7.6	37.497	0	79557000	11	0.302524	-0.810609	0.291801	1.53864
Pcbp2;Pcbp3	Poly(rC)-binding protein 2;Poly(rC)-	Q61990;Q6199	Q61990;Q61990-2;Q6199	17.5	17.5	17.5	20.6393	21.896	23.3251	17.5	17.5	20.437	4	2	2	15.5	6.9	6.9	38.221	0.002326	17464000	4	2.34062	-4.45345	0.427243	-0.978984
Pcca	Propionyl-CoA carboxylase alpha ch	Q91ZA3;H3BL62	Q91ZA3	20.2679	28.4499	28.1231	27.0924	27.8866	27.6177	23.7953	17.5	22.019	26	26	26	40.5	40.5	40.5	79.921	0	1.3E+09	99	0.288771	-1.91863	0.620678	4.50887
Pcna	Proliferating cell nuclear antigen	P17918;A0A140	P17918;A0A140T8V5	17.5	20.8229	17.5	17.5	17.5	17.5	17.5	17.5	23.52	2	2	2	12.3	12.3	12.3	28.785	0	21653000	2	0.427243	1.10764	0.145763	-0.899035
Pdap1	28 kDa heat- and acid-stable phosph	Q3UHX2	Q3UHX2	22.2794	23.5466	23.5664	21.1153	17.5	21.0516	17.5	23.1669	24.3355	3	3	3	22.1	22.1	22.1	20.605	0	1.08E+08	11	1.20142	3.24184	0.272392	1.46332
Pdcd5	Programmed cell death protein 5	P56812;D3Z7Q5	P56812;D3Z7Q5	21.8637	21.0461	22.3972	23.5238	17.5	17.5	17.5	23.5472	25.361	3	3	3	27.8	27.8	27.8	14.275	0	1.86E+08	8	0.480018	2.26104	0.0524411	-0.367065
Pdia3	Protein disulfide-isomerase A3	P27773;F6Q404	P27773	24.3463	23.7969	23.5574	26.0049	23.6897	24.911	19.8536	23.6971	26.1458	11	11	11	27.3	27.3	27.3	56.678	0	2.96E+08	26	0.613701	-0.968293	0.133263	0.668016
Pdia6	Protein disulfide-isomerase A6	Q922R8;Q3TML(	Q922R8;Q3TML0	19.821	21.281	17.5	21.9112	17.5	21.6994	17.5	17.5	21.7222	1	1	1	3.9	3.9	3.9	48.1	0	18848000	4	0.175187	-0.836205	0.128708	0.626599
Pebp1	Phosphatidylethanolamine-binding	P70296	P70296	17.5	17.5	17.5	22.1515	17.5	17.5	17.5	17.5	23.1965	1	1	1	13.9	13.9	13.9	20.83	0.002252	14272000	2	0.427243	-1.5505	0.427243	-1.89885
Pgam1	Phosphoglycerate mutase 1	Q9DBJ1;O7025	Q9DBJ1	22.1984	23.7013	24.7638	23.8953	17.5	17.5	17.5	21.9737	24.3944	4	4	4	28	28	28	28.832	0	1.27E+08	15	0.803176	3.92271	0.453506	2.26513
Pgd	6-phosphogluconate dehydrogenas	Q9DCD0	Q9DCD0	17.5	23.2307	17.5	22.956	17.5	22.6943	17.5	17.5	23.6077	2	2	2	6.4	6.4	6.4	53.247	0	40302000	4	0.248936	-1.63985	0.0149091	-0.125671
Pgk1	Phosphoglycerate kinase 1	P09411;S4R2M	P09411	23.9124	24.8486	25.6076	27.6295	25.769	25.6762	22.4427	26.4045	27.4236	7	7	7	28.1	28.1	28.1	44.55	0	8.23E+08	32	0.911806	-1.56871	0.147815	-0.634041
Phb	Prohibitin	P67778;Q5SQG	P67778;Q5SQG5	17.5	17.5	21.8875	22.8825	17.5	22.5592	23.3586	21.137	23.3981	3	3	3	13.6	13.6	13.6	29.82	0	61240000	6	0.37142	-2.01806	1.04951	-3.66874
Phgdh	D-3-phosphoglycerate dehydrogena	Q61753;F6ZSB7	Q61753;F6ZSB7	22.456	23.9695	23.1151	24.0242	23.0727	23.9262	23.8879	17.5	24.0296	5	5	5	12.2	12.2	12.2	56.585	0	1.37E+08	13	0.391802	-0.494182	0.247493	1.37435
Pkm	Pyruvate kinase PKM	P52480;P52480	P52480;P52480-2	27.3191	28.3286	28.9804	29.4964	29.4162	29.3424	27.6407	28.8862	30.2417	24	24	24	55.6	55.6	55.6	57.844	0	7.13E+09	228	1.17135	-1.20899	0.328739	-0.713523
Pkp1	Plakophilin-1	P97350	P97350	24.6581	25.8702	28.2063	24.537	24.9808	22.8984	23.7153	25.3195	23.8685	14	14	14	24.9	24.9	24.9	80.895	0	7.84E+08	42	0.798556	2.10616	0.77186	1.94377
Plec	Plectin	Q9QXS1;Q9QXS1	Q9QXS1;Q9QXS1-3;Q9QXS1	23.2555	24.3882	24.6279	26.0672	17.5	26.1948	17.5	20.7746	26.0446	29	29	29	7.9	7.9	7.9	534.18	0	3.57E+08	55	0.103526	0.836532	0.452257	2.65083
Plin2	Perilipin-2	P43883;B1AXR3	P43883;B1AXR3;B1AXR5	17.5	20.7033	23.3188	21.4126	17.5	22.0385	17.5	17.5	20.9379	2	2	2	7.3	7.3	7.3	46.646	0	29274000	4	0.029065	0.190334	0.384821	1.86139
Plp2	Proteolipid protein 2	Q9R1Q7	Q9R1Q7	22.2273	17.5	17.5	22.2112	22.094	21.8072	17.5	17.5	22.0618	1	1	1	7.9	7.9	7.9	16.607	0.002315	28250000	5	0.872274	-2.96171	0.00828228	0.0551612
Pnn	Pinin	O35691;Q3TUQ	O35691;Q3TUQ5	17.5	17.5	17.5	23.6795	17.5	17.5	17.5	22.4454	22.6369	1	1	1	1.5	1.5	1.5	82.435	0	55505000	3	0.427243	-2.05983	0.93457	-3.36079
Pof1b	Protein POF1B	Q8K4L4	Q8K4L4	17.5	17.5	21.768	17.5	17.5	17.5	17.5	17.5	17.5	2	2	2	4.4	4.4	4.4	67.784	0.004348	35106000	2	0.427243	1.42266	0.427243	1.42266
Ppa1	Inorganic pyrophosphatase	Q9D819	Q9D819	17.5	21.1464	17.5	21.9153	17.5	21.2122	17.5	17.5	21.862	1	1	1	7.6	7.6	7.6	32.667	0	12518000	1	0.336805	-1.49372	0.0429167	-0.238542
Ppia;Gm5160	Peptidyl-prolyl cis-trans isomerase A	P17742;F8VPN3	P17742;F8VPN3	17.5	20.5659	22.8644	23.6078	22.8183	22.0127	20.6258	17.5	25.9461	3	3	3	17.7	17.7	17.7	17.971	0	1.77E+08	10	0.704681	-2.50283	0.132233	-1.04721
Ppm1g	Protein phosphatase 1G	Q61074;A0A0J9	Q61074;A0A0J9YVG0	17.5	17.5	17.5	17.5	17.5	17.5	17.5	17.5	17.5	1	1	1	2.2	2.2	2.2	58.727	0	0	1	-4.34E-08	0	-4.34E-08	0
Ppp1ca	Serine/threonine-protein phosphata	P62137	P62137	22.2254	22.5751	17.5	22.9003	17.5	19.3112	17.5	20.8098	23.7632	2	2	2	7.6	7.6	7.6	37.54	0	48486000	8	0.140112	0.862944	0.010238	0.0757936
Ppp1cb	Serine/threonine-protein phosphata	P62141;A0A0J9	P62141;A0A0J9YUG2	17.5	17.5	17.5	17.5	22.5311	22.2899	17.5	17.5	22.2821	1	1	1	4	4	4	37.186	0.004274	17349000	1	0.934209	-3.27367	0.427243	-1.59403
Ppp2r1a;Ppp2r1b	Serine/threonine-protein phosphata	Q76MZ3;Q7TNP	Q76MZ3;Q7TNP2;G3UXQ1;	20.7131	17.5	24.2686	23.3655	20.9056	24.0648	20.9993	17.5	24.0782	2	2	2	5.4	5.4	5.4	65.322	0	96399000	4	0.37601	-1.95136	0.00382904	-0.031908
Pqbp1	Polyglutamine-binding protein 1	Q91VJ5	Q91VJ5	17.5	17.5	17.5	17.5	17.5	17.5	17.5	17.5	17.5	1	1	1	9.5	9.5	9.5	30.597	0.002283	13109000	2	-4.34E-08	0	-4.34E-08	0
Prdx1	Peroxiredoxin-1	P35700;B1AXW	P35700;B1AXW5;B1AXW6	24.17	23.6759	26.9577	23.4383	24.7365	19.4462	21.1972	25.8163	25.8551	4	4	4	20.1	20.1	20.1	22.176	0	4.57E+08	21	0.561796	2.39418	0.127636	0.644993
Prdx2	Peroxiredoxin-2	Q61171;D3Z4A4	Q61171;D3Z4A4	17.5	23.5306	25.056	23.0674	23.0871	21.0111	20.7495	23.2483	22.8384	3	3	3	24.2	24.2	24.2	21.778	0	1.4E+08	10	0.0513584	-0.359679	0.0347222	-0.249826
Prdx4	Peroxiredoxin-4	O08807;B1AZS9	O08807;B1AZS9	18.146	23.1404	22.3398	22.9981	21.3748	17.5	17.5	21.3667	23.9282	4	4	4	16.1	16.1	16.1	31.052	0	51701000	8	0.0926972	0.584452	0.0387726	0.277107

Prdx6	Peroxiredoxin-6	O08709;A0A0A6	O08709;A0A0A6YXQ7;Q6G	17.5	22.0556	17.5	22.5659	17.5	22.0982	17.5	17.5	22.8952	1	1	1	9.4	9.4	9.4	24.87	0	36154000	4	0.313886	-1.70283	0.0404389	-0.279853
Prep	Prolyl endopeptidase	Q9QUR6	Q9QUR6	17.5	17.5	17.5	22.6799	17.5	17.5	17.5	17.5	22.8954	2	2	2	4.5	4.5	4.5	80.751	0	21946000	3	0.427243	-1.72663	0.427243	-1.79846
Prim2	DNA primase large subunit	P33610	P33610	25.6557	26.5155	28.0001	24.9167	24.7042	23.5249	22.8207	24.922	17.5	2	2	2	4.8	4.8	4.8	58.408	0.003914	8.02E+08	4	1.35139	2.34182	1.00981	4.9762
Prpf38a	Pre-mRNA-splicing factor 38A	Q4FK66;Q4FK66	Q4FK66;Q4FK66-2	20.3008	17.5	17.5	17.5	17.5	20.6946	17.5	17.5	22.2145	1	1	1	3.8	3.8	3.8	37.436	0	19671000	6	0.0312273	-0.131238	0.128012	-0.637871
Prpf40a	Pre-mRNA-processing factor 40 hom	Q9R1C7;Q9R1C7	Q9R1C7;Q9R1C7-2;A0A1B0	17.5	17.5	17.5	20.5751	17.5	17.5	17.5	17.5	17.5	1	1	1	1.7	1.7	1.7	108.48	0	20232000	2	0.427243	-1.02502	-4.34E-08	0
Psma1	Proteasome subunit alpha type-1	Q9R1P4	Q9R1P4	20.5372	21.0204	17.5	20.85	17.5	17.5	17.5	17.5	17.5	1	1	1	5.7	5.7	5.7	29.546	0	5538600	2	0.273334	1.0692	0.927118	2.18585
Psma3	Proteasome subunit alpha type-3	O70435;E0CZ34	O70435;E0CZ34;F8WH02;E	21.5416	21.7751	17.5	21.4407	17.5	21.8466	17.5	17.5	21.6966	1	1	1	7.5	7.5	7.5	28.405	0	24768000	3	0.00163637	0.00983556	0.280501	1.37336
Psma6	Proteasome subunit alpha type-6	Q9QUM9;E0CXB	Q9QUM9;E0CXB1	17.5	17.5	17.5	17.5	17.5	17.5	17.5	17.5	23.7713	1	1	1	7.3	7.3	7.3	27.372	0	21704000	3	-4.34E-08	0	0.427243	-2.09044
Psma7;Psma8	Proteasome subunit alpha type-7;Pr	Q9Z2U0;Q9CWH	Q9Z2U0;Q9CWH6	17.5	22.5302	23.6504	23.4114	22.0591	22.4693	17.5	22.3642	22.1121	2	2	2	14.9	14.9	14.9	27.855	0	75564000	9	0.29806	-1.41975	0.0814281	0.568132
Psmb5	Proteasome subunit beta type-5	O55234	O55234	17.5	20.7624	22.7265	20.5724	17.5	17.5	17.5	17.5	17.5	1	1	1	5.3	5.3	5.3	28.532	0	18152000	1	0.41889	1.8055	0.863411	2.82963
Psmc2	26S protease regulatory subunit 7	P46471;Q8BVQ9	P46471;Q8BVQ9	17.5	17.5	17.5	17.5	17.5	22.4192	21.0102	17.5	22.6617	1	1	1	3	3	3	48.647	0	19222000	4	0.427243	-1.63972	0.884932	-2.89064
Psmc6	26S protease regulatory subunit 10I	P62334	P62334	19.7751	21.2843	19.0444	21.5465	17.5	17.5	17.5	19.2651	21.4925	2	2	2	5.9	5.9	5.9	44.172	0	14056000	3	0.324364	1.18579	0.175464	0.615444
Psmd11	26S proteasome non-ATPase regulat	Q8BG32;G3UZ2	Q8BG32;G3UZ28;G3UWW	17.5	21.5869	17.5	17.5	17.5	17.5	17.5	17.5	23.3943	1	1	1	2.8	2.8	2.8	47.436	0.007394	15456000	1	0.427243	1.36231	0.0896573	-0.602452
Psmd12	26S proteasome non-ATPase regulat	Q9D8W5;Q3TRH	Q9D8W5;Q3TRH2;B1AT36	19.2911	20.5845	17.5	21.3734	17.5	17.5	17.5	17.5	22.0991	1	1	1	4.6	4.6	4.6	52.895	0.002331	9423000	1	0.0747117	0.334082	0.0172508	0.0922057
Psmd2;Gm5422	26S proteasome non-ATPase regulat	Q8VDM4;J3KMC	Q8VDM4;J3KMQ2	17.5	21.1492	17.5	22.2864	17.5	21.8914	19.8828	17.5	24.312	4	4	4	6.1	6.1	6.1	100.2	0	38367000	8	0.398139	-1.84286	0.324898	-1.84851
Psmd3	26S proteasome non-ATPase regulat	P14685	P14685	17.5	21.9179	17.5	22.2838	17.5	22.0587	17.5	17.5	23.3091	1	1	1	2.8	2.8	2.8	60.718	0.003953	33684000	2	0.312828	-1.64154	0.0664596	-0.463734
Psmd6	26S proteasome non-ATPase regulat	Q99JI4	Q99JI4	17.5	17.5	17.5	21.8596	17.5	17.5	17.5	17.5	22.7264	1	1	1	4.4	4.4	4.4	45.536	0.004338	14170000	3	0.427243	-1.45321	0.427243	-1.74213
Psmd7	26S proteasome non-ATPase regulat	P26516	P26516	17.5	17.5	17.5	17.5	17.5	17.5	17.5	17.5	17.5	2	2	2	15	15	15	36.539	0	10522000	2	-4.34E-08	0	-4.34E-08	0
Psme2	Proteasome activator complex subu	P97372;G3X9V0	P97372;G3X9V0;E0CZ90	17.5	21.0407	24.6751	22.0788	17.5	17.5	17.5	21.6948	17.5	2	2	1	9.6	9.6	5	27.057	0	42660000	5	0.326898	2.04563	0.363007	2.17366
Ptbp1	Polypyrimidine tract-binding protei	Q8CB58;Q8BGJ5	Q8CB58;Q8BGJ5;Q922I7;P	17.5	21.1386	23.0559	24.0157	17.5	21.1129	17.5	21.2908	24.4916	4	4	4	11	11	11	52.63	0	56412000	6	0.0425974	-0.311349	0.0714076	-0.529277
Ptges3	Prostaglandin E synthase 3	Q9R0Q7;D3Z7C6	Q9R0Q7;D3Z7C6	17.5	17.5	17.5	17.5	17.5	17.5	17.5	17.5	21.8447	1	1	1	6.2	6.2	6.2	18.721	0.004184	6348200	1	-4.34E-08	0	0.427243	-1.44824
Ptgs2	Prostaglandin G/H synthase 2	Q05769	Q05769	17.5	17.5	22.4135	17.5	17.5	17.5	17.5	17.5	21.099	3	3	3	7.3	7.3	7.3	69.012	0	14867000	3	0.427243	1.63784	0.0758858	0.438174
Ptma	Prothymosin alpha;Prothymosin al	P26350;A0A087	P26350;A0A087WP98;A0A	23.2352	21.2731	17.5	20.0613	17.5	23.4507	17.5	17.5	23.2666	2	2	2	15.3	15.3	15.3	12.254	0	55253000	6	0.0472016	0.332071	0.186442	1.24723
Ptrh2	Peptidyl-tRNA hydrolase 2, mitocho	Q8R2Y8	Q8R2Y8	17.5	17.5	17.5	23.0983	17.5	17.5	17.5	17.5	22.7879	1	1	1	7.7	7.7	7.7	19.526	0.00231	18931000	2	0.427243	-1.86612	0.427243	-1.76263
Qars		D3Z158;Q8BML9	D3Z158;Q8BML9	17.5	17.5	17.5	17.5	17.5	17.5	17.5	17.5	22.0287	1	1	1	2.5	2.5	2.5	85.054	0.003869	4278700	1	-4.34E-08	0	0.427243	-1.50958
Rab10	Ras-related protein Rab-10	P61027	P61027	17.5	17.5	17.5	17.5	17.5	17.5	17.5	17.5	17.5	2	1	1	11.5	6	6	22.541	0.009225	7707700	1	-4.34E-08	0	-4.34E-08	0
Rab1A;Rab1b;Rab1	Ras-related protein Rab-1A;Ras-relat	P62821;Q9D1G1	P62821;Q9D1G1;Q5SW88;	17.5	21.68	23.7972	22.4907	17.5	17.5	17.5	17.5	21.4737	3	3	2	18.5	18.5	13.2	22.677	0	29388000	4	0.29837	1.82884	0.403766	2.16783
Rab2a	Ras-related protein Rab-2A	P53994	P53994	17.5	21.359	23.5177	22.9657	17.5	17.5	17.5	17.5	20.9208	2	2	2	14.2	14.2	14.2	23.547	0	29346000	5	0.227124	1.47036	0.440295	2.15199
Rab7a	Ras-related protein Rab-7a	P51150	P51150	17.5	24.2154	17.5	24.8273	23.3903	24.2691	17.5	17.5	24.3646	1	1	1	6.8	6.8	6.8	23.489	0	1.21E+08	2	0.906574	-4.42379	0.00508937	-0.0497265
Ran;1700009N14Rik	GTP-binding nuclear protein Ran	P62827;Q14AA6	P62827;Q14AA6	22.0847	23.2499	22.6071	22.6323	17.5	23.1126	21.6306	17.5	24.6578	5	5	4	25.9	25.9	20.8	24.423	0	94683000	15	0.356643	1.56561	0.262769	1.38444
Rars	Arginine--tRNA ligase, cytoplasmic	Q9D0I9	Q9D0I9	17.5	21.081	21.0685	22.2295	17.5	23.3176	17.5	17.5	23.7265	5	5	5	10.6	10.6	10.6	75.673	0	36605000	5	0.20362	-1.13251	0.0438734	0.307667
Rb1cc1	RB1-inducible coiled-coil protein 1	Q9ESK9;F7CCJ3;	Q9ESK9;F7CCJ3;F7CC56	23.7416	22.8811	17.5	22.6239	17.5	17.5	17.5	17.5	21.0092	2	2	2	1.3	1.3	1.3	182.35	0.005556	42505000	3	0.346108	2.16629	0.522087	2.70451
Rbbp4	Histone-binding protein RBBP4	Q60972	Q60972	17.5	17.5	17.5	21.6518	21.8898	17.5	17.5	17.5	21.8357	1	1	1	3.3	3.3	3.3	47.655	0.004283	12325000	2	0.933953	-2.84722	0.427243	-1.44523
Rbm3	Putative RNA-binding protein 3	O89086;Q8BG13	O89086;Q8BG13	20.4256	20.7278	17.5	17.5	17.5	17.5	17.5	17.5	23.088	3	3	3	26.1	26.1	26.1	16.604	0	16698000	4	0.931538	2.05113	0.0297969	0.188459
Rbm39	RNA-binding protein 39	Q8VH51;F7AA45	Q8VH51;F7AA45;Q8VH51-3	21.9158	22.1133	24.3008	23.3438	22.1717	23.7706	20.9421	23.9891	23.9093	3	3	3	7.9	7.9	7.9	59.406	0	2.55E+08	22	0.129882	-0.318701	0.0461745	-0.170183
Reep5	Receptor expression-enhancing pro	Q60870;G3X8R0	Q60870;G3X8R0	17.5	22.7439	17.5	22.2671	17.5	17.5	17.5	17.5	17.5	1	1	1	5.4	5.4	5.4	21.05	0.004219	17049000	1	0.022464	0.158927	0.427243	1.74795
Rnf17	RING finger protein 17	Q99MV7;Q99MV	Q99MV7;Q99MV7-4;Q99M	17.5	21.8243	17.5	22.503	17.5	17.5	17.5	17.5	17.5	1	1	1	0.5	0.5	0.5	185.6	1	15364000	0	0.034706	-0.226226	0.427243	1.44145
Rnf213	E3 ubiquitin-protein ligase RNF213	A0A171EBL2;E9	A0A171EBL2;E9Q555	17.5	20.4258	17.5	20.4465	17.5	23.2391	17.5	17.5	21.0616	2	2	2	0.6	0.6	0.6	584.62	0	16637000	2	0.426533	-1.91991	0.0472259	-0.211915
Rnps1	RNA-binding protein with serine-ric	Q99M28;Q99M2	Q99M28;Q99M28-2	23.9367	17.5	24.2971	24.875	17.5	21.5047	19.2392	23.3872	25.3884	5	5	5	22.6	22.6	22.6	34.208	0	5.65E+08	45	0.0704634	0.618041	0.0951736	-0.760326
Rpl10;Rpl10l	60S ribosomal protein L10;60S ribo	Q6ZWV3;I7HLV2	Q6ZWV3;I7HLV2;P86048	18.4045	17.5	22.0718	24.6417	21.8465	24.6433	17.5	22.9795	24.0486	4	4	4	16.4	16.4	16.4	24.604	0	1.26E+08	10	1.22614	-4.3851	0.371217	-2.18394
Rpl10a	60S ribosomal protein L10a;Ribosom	P53026;Q5XJF6;	P53026;Q5XJF6;D6RE43	20.8594	17.5	24.3819	24.582	20.8388	23.353	20.8146	17.5	25.1045	4	4	4	25.3	25.3	25.3	24.916	0	2.16E+08	11	0.370501	-2.01083	0.0255197	-0.225903
Rpl11;Gm10036;Gm509	60S ribosomal protein L11	Q9CXW4;E9PYL9	Q9CXW4;E9PYL9;A2BH06;E	21.6685	24.3538	24.4384	24.085	22.6863	25.5908	17.5	23.1174	24.0992	2	2	2	14.6	14.6	14.6	20.252	0	1.72E+08	16	0.196932	-0.6338	0.354243	1.9147
Rpl12	60S ribosomal protein L12	P35979	P35979	22.9003	17.5	23.6694	24.4593	17.5	24.2055	17.5	17.5	25.1945	2	2	2	14.5	14.5	14.5	17.804	0	2.32E+08	10	0.0825131	-0.698404	0.149648	1.29171
Rpl13	60S ribosomal protein L13	P47963;A0A140	P47963;A0A140T8K8	21.6292	22.8582	23.7737	23.995	17.5	24.6158	17.5	22.1216	25.339	7	7	7	34.6	34.6	34.6	24.305	0	1.83E+08	12	0.109928	0.716785	0.177097	1.10018
Rpl13a	60S ribosomal protein L13a	P19253;A0A1B0	P19253;A0A1B0GSB2;A0A1	22.7572	23.3159	25.6061	25.2471	25.1779	25.8624	24.5469	25.2129	25.0482	4	4	4	20.7	20.7	20.7	23.464	0	4.04E+08	22	0.789247	-1.53605	0.511003	-1.04295
Rpl14	60S ribosomal protein L14	Q9CR57	Q9CR57	21.8931	22.6308	24.3507	24.6449	17.5	24.5768	22.1475	24.3933	24.4899	3	3	3	17.1	17.1	17.1	23.564	0	2.11E+08	12	0.104213	0.717602	0.272798	-0.718702
Rpl15;Gm10020	60S ribosomal protein L15;Ribosom	Q9CZM2;E9QAZ2	Q9CZM2;E9QAZ2;B8JKK2	23.7914	23.3736	25.9582	25.2849	17.5	24.5031	22.769	24.314	27.631	9	9	9	35.8	35.8	35.8	24.146	0	6.06E+08	33	0.304345	1.94507	0.117468	-0.5303
Rpl17	60S ribosomal protein L17	Q9CPR4;Q6ZWZ	Q9CPR4;Q6ZWZ7	19.1137	20.814	22.4592	24.3967	17.5	26.7961	21.7469	22.8392	25.6409	8	8	8	43.5	43.5	43.5	21.423	0	2.62E+08	18	0.287901	-2.10198	0.800318	-2.61339
Rpl18	60S ribosomal protein L18	P35980;A0A1B0	P35980;A0A1B0GQU8;A0A	25.4925	26.2097	27.0106	28.2943	27.3283	27.6196	25.5088	28.1976	28.5271	7	7	7	31.4	31.4	31.4	21.644	0	2.87E+09	71	1.3483	-1.50979	0.485466	-1.17356
Rpl18a;Gm17541	60S ribosomal protein L18a	P62717;A0A1D5	P62717;A0A1D5RM85;A0A	21.1015	22.1045	22.9358	23.0979	23.8377	24.6004	22.7384	17.5	22.5874	4	4	4	21.6	21.6	21.6	20.732	0	91485000	14	1.23272	-1.79805	0.242113	1.10533
Rpl19	60S ribosomal protein L19	P84099;A2A547	P84099;A2A547	22.6013	23.9577	24.4987	24.3883	21.4731	24.1657	19.8859	23.064	25.5505	4	4	4	18.4	18.4	18.4	23.466	0	2.28E+08	24	0.113993	0.343529	0.187959	0.852456
Rpl21	60S ribosomal protein L21	O09167;Q9CQM	O09167;Q9CQM8	18.6505	20.7472	23.4289	23.1835	17.5	17.5	17.5	20.71	23.9825	2	2	2	16.2	16.2	16.2	18.562	0	69745000	8	0.263283	1.54771	0.0305882	0.21136
Rpl22	60S ribosomal protein L22	P67984	P67984	21.9765	17.5	17.5	23.3617	23.5596	23.8378	21.8076	22.9376	23.7421	2	2	2	19.5	19.5	19.5	14.759	0	85581000	12	1.42661	-4.59423	1.13192	-3.83694
Rpl23	60S ribosomal protein L23	P62830;A2A6F8	P62830;A2A6F8	17.5	18.2298	21.7781	21.8923	17.5	24.8809	20.013	17.5	22.6292	2	2	2	25	25	25	14.865	0	50393000	5	0.375615	-2.25508	0.166835	-0.878094
Rpl23a	60S ribosomal protein L23a	P62751;A0A140	P62751;A0A140T8M7	20.2816	24.5681	25.3713	24.3717	17.5	17.5	17.5	20.8414	21.4505	2	2	2	15.4	15.4	15.4	17.695	0.003968	1.11E+08	4	0.579139	3.61643	0.803222	3.47638
Rpl24	60S ribosomal protein L24	Q8BP67	Q8BP67	19.8328	20.6177	22.7022	22.0357	21.1948	23.4382	17.5	17.5	21.7324	2	2	2	14	14	14	17.779	0	39088000	2	0.471174	-1.17197	0.577666	2.14011
Rpl27a	60S ribosomal protein L27a	P14115	P14115	21.0236	22.5691	22.5463	23.4865	21.7572	23.9729	17.5	22.7156	23.2445	3	3	3	14.9	14.9	14.9	16.605	0	83319000	13	0.535562	-1.02588	0.178261	0.892959
Rpl28	60S ribosomal protein L28	P41105;F6Z0X0	P41105	17.5	17.5	17.5	25.915	24.149	24.3233	17.5	20.182	25.4688	5	5	5	29.2	29.2	29.2	15.733	0	1.83E+08	11	3.69253	-7.29577	0.690438	-3.55027
Rpl29;Gm17669;Gm107	60S ribosomal protein L29	P47915;F6QL70	P47915;F6QL70;W4VSN7;D	23.1675	21.8412	22.6922	24.3126	17.5	23.0589	17.5	21.9689	23.2204	4	4	4	13.8	13.8	13.8	17.587	0	1.2E+08	8	0.167048	0.94311	0.396975	1.67053
Rpl3	60S ribosomal protein L3	P27659;A0A087	P27659	23.383	17.5	23.4349	25.2044	23.2383	24.8658	20.942	22.6435	24.8195	4	4	4	16.1	16.1	16.1	46.109	0	2.09E+08	15	0.658349	-2.9969	0.236382	-1.36235
Rpl30	60S ribosomal protein L30	P62889	P62889	17.5	22.482	17.5	24.9841	17.5	17.5	21.537	17.5	24.7906	3	3	3	34.8	34.8	34.8	12.784	0	1.39E+08	5	0.0998662	-0.834046	0.323548	-2.11523
Rpl34;Gm2178	60S ribosomal protein L34	Q9D1R9;A0A0G2	Q9D1R9;A0A0G2JGY8;A0A0	19.3306	21.8527	22.0631	22.8859	21.7652	24.5021	17.5	17.5	23.9399	4	4	4	21.4	21.4	21.4	13.293	0	72643000	6	0.765373	-1.96891	0.244542	1.4355
Rpl38	60S ribosomal protein L38	Q9JJI8	Q9JJI8	17.5	19.8013	21.4983	21.6302	17.5	17.5	17.5	17.5	23.2922	2	2	2	35.7	35.7	35.7	8.2038	0	55573000	4	0.149771	0.723134	0.025153	0.16914
Rpl4	60S ribosomal protein L4	Q9D8E6	Q9D8E6	17.5	25.0053	25.9481	24.9975	24.7918	26.2784	22.4067	25.2469	26.5258	8	8	8	29.1	29.1	29.1	47.153	0	7.2E+08	23	0.395272	-2.53812	0.258696	-1.90869
Rpl5	60S ribosomal protein L5	P47962	P47962	17.5	17.5	17.5	17.5	17.5	17.5	17.5	17.5	20.8357	1	1	1	4.4	4.4	4.4	34.4	0.004149	1871400	1	-4.34E-08	0	0.427243	-1.1119
Rpl6;Gm5428	60S ribosomal protein L6	P47911;E9PUX4	P47911;E9PUX4	24.2414	23.7702	24.7494	25.4446	23.4251	23.3566	22.5332	24.3473	25.941	9	9	9	26	26	26	33.509	0	5.57E+08	57	0.0852693	0.178229	0.00646465	-0.0201791
Rpl7	60S ribosomal protein L7	P14148;F6XI62	P14148;F6XI62	26.4099	25.8908	27.2278	26.9168	21.0453	24.3582	23.0909	26.5244	25.9543	12	12	12	39.6	39.6	39.6	31.419	0	1.1E+09	64	0.619323	2.40277	0.511113	1.31967
Rpl7a	60S ribosomal protein L7a	P12970;A0A140	P12970;A0A140T8L1;A0A1	22.9999	24.2644	25.3775	24.7004	24.0526	25.4807	23.4598	25.0289	25.4644	6	6	6	18.8	18.8	18.8	29.976	0	3.69E+08	30	0.264348	-0.530637	0.18128	-0.437082
Rpl8	60S ribosomal protein L8	P62918	P62918	21.6744	22.8867	23.865	23.3504	23.085	24.8914	17.5	23.6443	24.3288	4	4	4	14	14	14	28.024	0	1.51E+08	21	0.498004	-0.966886	0.163764	0.984344
Rpl9	60S ribosomal protein L9	P51410;A0A140	P51410;A0A140T8T4;D3YZ	17.5	17.5	21.1324	24.8382	24.339	27.0525	17.5	17.5	23.5029	3	3	3	22.4	22.4	22.4	21.881	0	2.29E+08	13	1.98446	-6.6991	0.123517	-0.790165
Rplp0	60S acidic ribosomal protein P0	P14869;D3YVM5	P14869;D3YVM5;S4R1N1	17.5	24.2117	23.497	25.2287	23.1994	23.2644	17.5	25.4867	24.725	3	3	3	20.2	20.2	20.2	34.216	0	2.13E+08	16	0.411933	-2.16127	0.0894712	-0.834318
Rplp2	60S acidic ribosomal protein P2	P99027	P99027	21.0347	24.2331	25.4125	21.7951	17.5	20.1508	22.1622	24.519	26.5191	7	7	7	64.3	64.3	64.3	11.651	0	3.03E+08	22	0.96922	3.74481	0.175465	-0.839996
Rpn1	Dolichyl-diphosphooligosaccharide	Q91YQ5	Q91YQ5	17.5	21.2084	17.5	21.6794	17.5	20.4899	17.5	17.5	20.5683	2	2	2	3.9	3.9	3.9	68.527	0.004396	8809200	2	0.262435	-1.15365	0.045458	0.213374
Rpn2	Dolichyl-diphosphooligosaccharide	Q9DBG6;A2ACG7	Q9DBG6;A2ACG7	17.5	25.653	26.5885	20.6085	17.5	17.5	17.5	17.5	21.4315	2	2	2	6.3	6.3	6.3	69.062	0	1.58E+08	2	0.700522	4.71102	0.63042	4.43667
Rps11	40S ribosomal protein S11	P62281;A0A1B0	P62281;A0A1B0GRR3;A0A1	20.8593	17.5	22.2095	23.9577	17.5	23.7553	21.3821	24.5945	25.2406	6	6	6	38.6	38.6	38.6	18.431	0	1.29E+08	10	0.240202	-1.5481	0.900015	-3.54946
Rps13	40S ribosomal protein S13	P62301;Q921R2	P62301;Q921R2	22.4117	23.3919	24.1256	24.7961	22.8627	23.1715	21.6382	22.8931	24.7879	8	8	8	40.4	40.4	40.4	17.222	0	1.82E+08	12	0.143125	-0.300394	0.0681746	0.203323
Rps14	40S ribosomal protein S14	P62264;D3YVF4	P62264	22.6667	23.4476	24.4327	23.4202	23.6155	24.6267	17.5	23.2342	24.7594	5	5	5	18.5	18.5	18.5	16.273	0	1.47E+08	12	0.230189	-0.371792	0.301948	1.68446
Rps16	40S ribosomal protein S16	P14131	P14131	17.5	20.7429	20.9112	21.5108	17.5	20.8566	17.5	17.5	22.3496	2	2	2	15.1	15.1	15.1	16.445	0.002288	15808000	2	0.0489443	-0.237758	0.111063	0.601503
Rps18;Gm10260	40S ribosomal protein S18	P62270;S4R1N6	P62270;S4R1N6;F6YVP7	20.3477	20.626	21.7717	21.0868	21.2381	21.1348	17.5	21.2519	22.4357	1	1	1	7.2	7.2	7.2	17.718	0.004175	25801000	3	0.210714	-0.238131	0.122338	0.519219
Rps19	40S ribosomal protein S19	Q9CZX8;S4R223	Q9CZX8;S4R223;D3YUT3;D	17.5	17.5	21.5537	17.5	17.5	17.5	17.5	17.5	21.3565	2	2	2	13.8	13.8	13.8	16.085	0.002294	9460200	2	0.427243	1.35123	0.011629	0.0657196
Rps2;Gm6576;Gm5786;(	40S ribosomal protein S2	P25444;F6YTZ4;	P25444;F6YTZ4;J3QMG5;A(	20.6831	22.6559	24.0659	23.3561	22.377	21.8449	22.2525	22.7272	24.3588	4	4	4	15.4	15.4	15.4	31.231	0	95790000	5	0.017813	-0.0576998	0.213903	-0.644542
Rps20	40S ribosomal protein S20	P60867	P60867	20.3403	21.5898	17.5	22.9343	17.5	17.5	17.5	17.5	24.0272	2	2	2	22.7	22.7	22.7	13.373	0	37732000	8	0.0808244	0.498598	0.0179226	0.134281
Rps21	40S ribosomal protein S21	Q9CQR2	Q9CQR2	17.5	17.5	17.5	17.5	17.5	17.5	17.5	17.5	22.5591	2	2	2	34.9	34.9	34.9	9.1413	0	6179600	2	-4.34E-08	0	0.427243	-1.68636
Rps23	40S ribosomal protein S23	P62267	P62267	22.7722	23.2395	23.1051	24.7958	21.6712	23.4124	19.3787	23.1878	24.2491	4	4	4	21.7	21.7	21.7	15.807	0	1.64E+08	12	0.099712	-0.254164	0.19876	0.767062
Rps25	40S ribosomal protein S25	P62852	P62852	17.5	23.1542	23.7201	23.9917	22.7229	23.2106	17.5	21.6134	24.0356	3	3	3	22.4	22.4	22.4	13.742	0	1.01E+08	12	0.385654	-1.85029	0.0509751	0.408469
Rps27;Rps27l	40S ribosomal protein S27;40S ribo	Q6ZWU9;A0A0G	Q6ZWU9;A0A0G2JDW7;A0	17.5	22.5292	23.6412	23.7866	17.5	22.7041	17.5	17.5	23.8521	2	2	2	31	31	31	9.461	0	70682000	9	0.0130278	-0.106746	0.22066	1.60609
Rps27a;Ubc;Ubb;Uba52;(	Ubiquitin-40S ribosomal protein S2	P62983;P0CG50	P62983;P0CG50;P0CG49;A	26.9595	27.8137	27.5834	28.324	27.8641	27.8292	26.0935	27.1431	28.5489	7	7	7	39.1	39.1	39.1	17.951	0	2.73E+09	143	0.855073	-0.553593	0.0896433	0.19039
Rps3	40S ribosomal protein S3	P62908;A0A140	P62908;A0A140LI77;D3YV4	23.0745	24.546	25.3486	25.7164	25.2935	25.6175	24.1053	24.5953	26.6792	8	8	8	46.9	46.9	46.9	26.674	0	5.34E+08	44	0.834196	-1.21941	0.318873	-0.803565
Rps3a	40S ribosomal protein S3a	P97351	P97351	23.3276	24.323	24.4385	26.4248	24.2063	26.7617	21.7304	25.4448	26.8424	6	6	6	26.9	26.9	26.9	29.885	0	4.93E+08	30	0.94441	-1.76788	0.153416	-0.642861
Rps4x	40S ribosomal protein S4, X isoform	P62702;V9GWY0	P62702;V9GWY0	17.5	17.5	22.5549	22.9442	17.5	23.7845	17.5	17.5	23.6779	4	4	4	13.7	13.7	13.7	29.597	0	46702000	4	0.357381	-2.22459	0.0482167	-0.374336
Rps5	40S ribosomal protein S5;40S ribos	P97461;D3YYM6	P97461;D3YYM6;D3Z1S8;C	17.5	17.5	23.3222	23.4153	17.5	23.2812	17.5	17.5	24.9192	4	4	4	27.5	27.5	27.5	22.889	0	63595000	7	0.287418	-1.9581	0.0586096	-0.532309
Rps6	40S ribosomal protein S6	P62754	P62754	24.0829	23.0513	25.5635	25.5288	22.1898	25.3146	21.8126	22.377	25.1545	4	4	4	22.1	22.1	22.1	28.68	0	3.59E+08	30	0.0288628	-0.111817	0.370063	1.11787
Rps8	40S ribosomal protein S8	P62242	P62242	24.6316	24.2628	25.71	25.4966	25.0081	26.036	22.9179	25.0892	26.1486	7	7	7	33.2	33.2	33.2	24.205	0	5.56E+08	55	0.542051	-0.64544	0.0490779	0.149562
Rps9	40S ribosomal protein S9	Q6ZWN5;F7CJS8	Q6ZWN5;F7CJS8;D3YWH9;	22.6854	24.2261	24.8733	25.3385	23.5126	24.2299	17.5	24.405	25.0875	6	6	6	24.7	24.7	24.7	22.591	0	2.56E+08	24	0.198223	-0.432068	0.25265	1.59742
Rpsa	40S ribosomal protein SA	P14206	P14206	24.8328	25.5095	26.008	25.2123	23.7714	24.9306	22.6199	25.9433	26.6554	8	8	8	42	42	42	32.838	0	6.93E+08	45	0.66012	0.811998	0.105471	0.377263
Rrbp1	Ribosome-binding protein 1	Q99PL5;A2AVJ7;	Q99PL5;A2AVJ7;Q99PL5-4;	24.7862	24.2645	26.2022	26.5534	28.0601	26.8353	24.8167	26.1071	25.8552	23	23	23	32.3	32.3	32.3	172.88	0	1.61E+09	69	1.30594	-2.06533	0.29408	-0.508708
Rrm2	Ribonucleoside-diphosphate reduct	P11157;D3Z0D8	P11157;D3Z0D8;F6RUC3	17.5	17.5	17.5	21.828	17.5	17.5	17.5	20.0197	22.5771	2	2	2	7.7	7.7	7.7	45.095	0	11043000	2	0.427243	-1.44265	0.798326	-2.53228
Rtn4	Reticulon-4	Q99P72;Q8BH78	Q99P72;Q8BH78;Q8BHF5	17.5	24.2119	24.0867	22.9771	21.7717	22.3984	17.5	17.5	17.5	3	3	3	5	5	5	126.61	0	54613000	5	0.0700708	-0.449507	0.934974	4.43287
S100a11	Protein S100-A11	P50543	P50543	24.035	26.0176	25.5333	25.2928	23.2493	22.7579	17.5	24.3978	25.1337	1	1	1	10.2	10.2	10.2	11.083	0.004016	3.34E+08	15	0.661006	1.42862	0.497201	2.85144

S100a14	Protein S100-A14	Q9D2Q8	Q9D2Q8	22.3832	23.672	26.5714	22.5768	22.5101	21.1555	21.3558	23.1284	22.7287	3	3	3	40.4	40.4	40.4	11.599	0	1.84E+08	13	0.738011	2.12803	0.59821	1.80455
S100a4	Protein S100-A4	P07091;A0A0G2	P07091;A0A0G2JGD2	17.5	17.5	22.0067	17.5	17.5	17.5	17.5	17.5	23.0378	2	2	2	15.8	15.8	15.8	11.721	0.004107	24063000	2	0.427243	1.50222	0.0495603	-0.343709
S100a6	Protein S100-A6	P14069	P14069	24.8318	25.3756	25.8433	26.0618	25.4993	25.0233	22.4841	25.126	26.2506	3	3	3	25.8	25.8	25.8	10.051	0	5.24E+08	18	0.159293	-0.177896	0.250758	0.729978
Sdc1	Syndecan-1	P18828;P18828	P18828;P18828-2	17.5	17.5	17.5	17.5	21.0334	17.5	17.5	21.409	21.7329	1	1	1	6.4	6.4	6.4	32.904	0.00565	10977000	2	0.427243	-1.17781	0.932759	-2.71397
Sec22b	Vesicle-trafficking protein SEC22b	O08547;E9Q6R3	O08547;E9Q6R3	17.5	17.5	17.5	17.5	17.5	17.5	17.5	17.5	22.3687	1	1	1	6.5	6.5	6.5	24.74	0.00431	5415800	1	-4.34E-08	0	0.427243	-1.62291
Sec61a1	Protein transport protein Sec61 sub	P61620;Q9JLR1;	P61620	17.5	22.1997	24.327	22.6536	17.5	21.756	17.5	17.5	21.7386	3	3	3	6.7	6.7	6.7	52.264	0	42538000	9	0.0984997	0.705683	0.420541	2.42938
Sec61b	Protein transport protein Sec61 sub	Q9CQS8	Q9CQS8	17.5	17.5	17.5	23.8147	24.0446	24.1332	22.6797	17.5	24.0814	1	1	1	15.6	15.6	15.6	9.9583	0	80360000	5	6.56418	-6.4975	0.914583	-3.92037
Serbp1	Plasminogen activator inhibitor 1 R	Q9CY58;Q9CY58	Q9CY58;Q9CY58-2;Q9CY58	23.3648	24.2961	24.1251	24.3176	17.5	21.9722	17.5	24.2345	26.0052	7	7	7	24.6	24.6	24.6	44.714	0	2.5E+08	21	0.589261	2.66541	0.199184	1.34879
Serpinh1	Serpin H1	P19324;A0A140	P19324;A0A140LHR4	23.1398	23.1219	23.618	23.8929	23.237	26.2195	17.5	24.5931	25.3261	3	3	3	9.4	9.4	9.4	46.533	0	2.83E+08	15	0.557837	-1.15661	0.119531	0.820141
Set	Protein SET	Q9EQU5;Q9EQU	Q9EQU5;Q9EQU5-2;A2BE93	17.5	17.5	22.5534	17.5	17.5	17.5	17.5	17.5	17.5	1	1	1	4.5	4.5	4.5	33.377	0	26705000	3	0.427243	1.68447	0.427243	1.68447
Sf1	Splicing factor 1	Q64213;Q64213	Q64213;Q64213-2;D3YVH4	20.0158	17.5	17.5	23	17.5	17.5	17.5	17.5	22.3978	1	1	1	2	2	2	70.402	0.004367	18944000	1	0.188709	-0.994761	0.162665	-0.794012
Sf3b3	Splicing factor 3B subunit 3	Q921M3-2;Q921	Q921M3-2;Q921M3	17.5	20.8428	21.0762	22.6139	17.5	22.6861	17.5	17.5	24.4321	3	3	3	3.7	3.7	3.7	125.31	0	43650000	9	0.211172	-1.12701	0.000553073	-0.00438372
Sf3b4	Splicing factor 3B subunit 4	Q8QZY9	Q8QZY9	17.5	17.5	17.5	20.3055	17.5	20.3766	17.5	17.5	20.2715	1	1	1	3.3	3.3	3.3	44.355	0.004301	3922800	2	0.934874	-1.89403	0.427243	-0.923834
Sfpq	Splicing factor, proline- and glutami	Q8VIJ6	Q8VIJ6	17.5	17.5	22.9156	22.8742	17.5	23.7767	17.5	17.5	23.0273	1	1	1	2.4	2.4	2.4	75.441	0.002268	49759000	4	0.31978	-2.07844	0.00473083	-0.037262
Shmt2	Serine hydroxymethyltransferase	Q9CZN7	Q9CZN7	17.5	17.5	17.5	21.7804	22.1813	17.5	17.5	21.4867	21.7965	1	1	1	2.2	2.2	2.2	55.758	0.002262	14940000	6	0.932141	-2.98726	0.933032	-2.76108
Slc25a24	Calcium-binding mitochondrial car	Q8BMD8	Q8BMD8	17.5	20.8594	21.7315	21.0615	19.9074	17.5	17.5	17.5	20.0294	1	1	1	2.7	2.7	2.7	52.901	0.009191	10907000	1	0.118414	0.540658	0.474816	1.68714
Slc25a3	Phosphate carrier protein, mitocho	Q8VEM8;G5E902	Q8VEM8;G5E902	23.2333	25.7729	26.5332	26.4896	26.1274	24.3102	24.3683	23.7907	25.4253	6	6	6	15.7	15.7	15.7	39.632	0	5.12E+08	23	0.142411	-0.462566	0.230943	0.651668
Slc25a5;Slc25a4	ADP/ATP translocase 2;ADP/ATP tra	P51881;P48962	P51881;P48962	21.4488	25.0432	27.3265	25.3832	26.2366	26.1901	24.4862	23.8095	26.5299	10	10	10	30.9	30.9	30.9	32.931	0	6.34E+08	33	0.313833	-1.33046	0.0614402	-0.335691
Slc39a7	Zinc transporter SLC39A7	Q31125	Q31125	17.5	17.5	17.5	21.5106	17.5	17.5	17.5	17.5	17.5	2	2	2	7.1	7.1	7.1	50.656	0.004098	4898300	2	0.427243	-1.33685	-4.34E-08	0
Slc3a2	4F2 cell-surface antigen heavy chain	P10852;A0A0U1	P10852;A0A0U1RPK4;P108	21.2414	23.0014	17.5	17.5	17.5	21.8418	17.5	17.5	17.5	1	1	1	3.2	3.2	3.2	58.336	0.003906	22821000	3	0.306138	1.63366	0.884943	3.08094
Smarca5	SWI/SNF-related matrix-associated a	Q91ZW3	Q91ZW3	17.5	17.5	17.5	17.5	17.5	23.1096	17.5	17.5	17.5	1	1	1	1.4	1.4	1.4	121.63	0	9050500	1	0.427243	-1.86986	-4.34E-08	0
Snd1	Staphylococcal nuclease domain-co	Q78PY7;Q3TJ56	Q78PY7;Q3TJ56	19.8867	21.36	24.0102	23.1683	22.0479	21.8732	17.5	21.9676	23.533	6	6	6	8.9	8.9	8.9	102.09	0	60132000	6	0.182869	-0.610853	0.126842	0.752075
Snrnp27	U4/U6.U5 small nuclear ribonucleo	Q8K194;Q8K194	Q8K194;Q8K194-2	19.9923	20.7534	23.151	22.2076	17.5	20.9262	17.5	20.5577	22.7009	3	3	3	17.4	17.4	17.4	18.885	0	40542000	8	0.254533	1.08761	0.22976	1.04603
Snrnp70	U1 small nuclear ribonucleoprotein	Q62376;A0A1B0	Q62376;A0A1B0GR44;Q62	17.5	17.5	17.5	21.7209	17.5	17.5	17.5	17.5	17.5	1	1	1	2.2	2.2	2.2	51.991	0.005639	3456500	1	0.427243	-1.40696	-4.34E-08	0
Srek1	Splicing regulatory glutamine/lysine	Q8BZX4;A0A1B0	Q8BZX4;A0A1B0GSB4;A0A1	17.5	17.5	17.5	17.5	17.5	17.5	17.5	17.5	22.1921	1	1	1	2.8	2.8	2.8	56.763	0	7566100	1	-4.34E-08	0	0.427243	-1.56403
Srrm1	Serine/arginine repetitive matrix pr	Q52KI8;A2A8V9;	Q52KI8;A2A8V9;A2A8V8;E9	17.5	20.901	23.909	17.5	17.5	22.0847	17.5	22.5638	22.9841	5	5	5	8.2	8.2	8.2	106.86	0	1.34E+08	19	0.293896	1.74178	0.0324682	-0.245955
Srrm2	Serine/arginine repetitive matrix pr	Q8BTI8;Q8BTI8-3	Q8BTI8;Q8BTI8-3;Q8BTI8-2	24.7639	22.9178	23.5775	25.2264	24.4718	24.0135	23.2959	24.598	24.9748	14	14	14	7.7	7.7	7.7	294.84	0	5.84E+08	58	0.562097	-0.817528	0.29277	-0.536507
Srsf1	Serine/arginine-rich splicing factor 1	Q6PDM2;H7BX9	Q6PDM2;H7BX95;Q6PDM2	25.8976	25.5236	25.215	25.5603	20.8314	23.6082	19.8612	25.3977	26.2301	10	10	10	42.3	42.3	42.3	27.744	0	5.82E+08	53	0.731064	2.21212	0.355454	1.71574
Srsf10	Serine/arginine-rich splicing factor 1	Q9R0U0;Q3TFP0	Q9R0U0;Q3TFP0;Q9R0U0-3	17.5	23.6571	17.5	24.3261	17.5	21.5294	17.5	17.5	23.6337	3	3	3	17.9	17.9	17.9	31.3	0	1.02E+08	3	0.213104	-1.56612	0.000878572	0.00780614
Srsf11		Q3UIX4;E9Q6E5;	Q3UIX4;E9Q6E5;F6RDI8;D3	17.5	19.6865	20.2643	19.5814	17.5	17.5	17.5	17.5	22.0179	4	4	4	13.9	13.9	13.9	53.102	0	38726000	9	0.366433	0.956484	0.0280938	0.144307
Srsf2	Serine/arginine-rich splicing factor 2	Q62093	Q62093	25.2969	24.1565	25.3819	25.3009	22.8313	24.7067	23.8541	25.6588	26.1373	4	4	4	21.7	21.7	21.7	25.476	0	6.69E+08	33	0.324378	0.665464	0.124292	-0.271677
Srsf3;Gm12355	Serine/arginine-rich splicing factor 3	P84104;P84104	P84104;P84104-2;A2A4X6	26.7015	26.107	26.4498	27.4138	23.9852	27.489	23.6125	27.8876	28.0125	8	8	7	44.5	44.5	44.5	19.329	0	2.11E+09	77	0.0357662	0.12344	0.0193673	-0.0847626
Srsf4	Serine/arginine-rich splicing factor 4	Q8VE97;Q542V3	Q8VE97;Q542V3	17.5	17.5	17.5	17.5	17.5	17.5	21.6908	17.5	22.7158	2	1	1	4.5	2.7	2.7	55.979	0	42365000	2	-4.34E-08	0	0.917869	-3.13554
Srsf5	Serine/arginine-rich splicing factor 5	O35326;Q9D8S5	O35326;Q9D8S5	21.6073	22.5718	17.5	24.1349	17.5	24.2017	17.5	23.2227	26.4748	3	2	2	10.8	7.4	7.4	30.891	0	2.55E+08	13	0.196304	-1.38585	0.237387	-1.83947
Srsf6		Q3TWW8;A0A0A	Q3TWW8	24.1733	22.2738	23.825	24.5989	23.3479	21.9195	22.8099	24.2511	24.5535	5	5	4	14.5	14.5	11.8	39.025	0	2.08E+08	25	0.0478048	0.135291	0.219591	-0.447427
Srsf7	Serine/arginine-rich splicing factor 7	Q8BL97;Q8BL97	Q8BL97;Q8BL97-3;Q8BL97	24.5967	23.8513	22.9902	26.5516	19.741	24.083	22.0616	23.252	26.622	6	5	5	24.7	24.7	24.7	30.817	0	5.18E+08	22	0.0600568	0.35419	0.0390398	-0.165792
Ssr3	Translocon-associated protein subu	Q9DCF9;Q9DCF9	Q9DCF9;Q9DCF9-2	17.5	17.5	17.5	21.4366	17.5	17.5	17.5	17.5	21.1165	1	1	1	7.6	7.6	7.6	21.064	0.003795	5112000	1	0.427243	-1.31222	0.427243	-1.20552
Ssrp1	FACT complex subunit SSRP1	Q08943;A2AW0	Q08943;A2AW05;Q08943-	17.5	17.5	17.5	22.1238	17.5	17.5	17.5	17.5	19.4615	2	2	2	2.8	2.8	2.8	80.859	0	10704000	2	0.427243	-1.54128	0.427243	-0.65384
Stat1	Signal transducer and activator of tr	P42225;Q8C3V4	P42225;Q8C3V4;A0A087W	17.5	22.1229	22.0769	23.6786	17.5	21.4147	19.3913	20.1899	21.9712	4	4	4	6.1	6.1	6.1	87.196	0	33378000	5	0.0428828	-0.297858	0.00944641	0.0491371
Stip1	Stress-induced-phosphoprotein 1	Q60864	Q60864	21.3147	22.453	22.6916	22.4733	21.9115	23.2055	19.9801	23.1369	23.5343	2	2	2	4.2	4.2	4.2	62.581	0	70196000	11	0.265921	-0.376979	0.0176874	-0.063996
Stmn1	Stathmin	P54227;D3Z1Z8	P54227;D3Z1Z8;D3Z5N2	22.3172	17.5	22.224	21.8737	17.5	23.7985	17.5	17.5	25.0083	2	2	2	18.1	18.1	18.1	17.274	0	1.36E+08	7	0.052978	-0.376974	0.0806876	0.677649
Stmn2	Stathmin-2	P55821	P55821	17.5	20.4403	17.5	21.2255	17.5	20.7097	17.5	17.5	17.5	1	1	1	5.6	5.6	5.6	20.828	0.004237	16669000	3	0.365305	-1.33164	0.427243	0.980101
Stt3a	Dolichyl-diphosphooligosaccharide	P46978	P46978	17.5	20.8341	21.6713	20.3646	17.5	17.5	17.5	17.5	21.5762	1	1	1	1.6	1.6	1.6	80.597	0.003854	11662000	1	0.413104	1.54691	0.242143	1.14305
Sumo2;Sumo3	Small ubiquitin-related modifier 2;S	P61957;Q9Z172	P61957;Q9Z172;G3UZX6;H	22.115	22.4212	23.4519	22.4638	17.5	17.5	17.5	23.9591	23.1481	1	1	1	12.6	12.6	12.6	10.871	0.002257	80377000	8	0.964541	3.50806	0.210917	1.12693
Supt16h;Supt16	FACT complex subunit SPT16	Q920B9;G3X956	Q920B9;G3X956	17.5	21.5015	17.5	22.1692	17.5	22.6948	17.5	17.5	22.3278	1	1	1	1.4	1.4	1.4	119.82	0.002273	23392000	5	0.387951	-1.95416	0.0450231	-0.275438
Syncrip	Heterogeneous nuclear ribonucleop	Q7TMK9;G3UXJ6	Q7TMK9;G3UXJ6;G3UZ48;(	19.6932	17.5	17.5	22.9332	17.5	22.7991	17.5	17.5	22.7228	1	1	1	2.1	2.1	2.1	69.632	0.004124	29451000	2	0.667931	-2.84634	0.20683	-1.00988
Tagln2	Transgelin-2	Q9WVA4;A0A0A	Q9WVA4	24.0667	25.5818	24.5065	25.0932	23.7367	23.5174	22.6958	24.6085	25.7771	9	9	9	44.7	44.7	44.7	22.395	0	4.02E+08	25	0.379215	0.602576	0.130979	0.357876
Tap1	Antigen peptide transporter 1	P21958;F6QHF1	P21958;F6QHF1;Q3TBA3	17.5	23.5799	24.2994	23.022	22.3326	25.0145	17.5	17.5	21.4127	3	3	3	6.4	6.4	6.4	78.863	0	89432000	8	0.292527	-1.66326	0.521028	2.98887
Tap2	Antigen peptide transporter 2	P36371	P36371	17.5	17.5	17.5	17.5	17.5	17.5	17.5	17.5	21.0138	1	1	1	2.6	2.6	2.6	77.444	0.005576	2117300	1	-4.34E-08	0	0.427243	-1.17126
Tardbp	TAR DNA-binding protein 43	Q921F2;A0A087	Q921F2;A0A087WR97;A0A	21.5352	22.6271	22.8808	22.6863	17.5	22.3571	17.5	21.5204	21.9827	3	3	3	10.1	10.1	10.1	44.547	0	41169000	10	0.362468	1.4999	0.609255	2.01332
Tcof1	Treacle protein	O08784;H3BL37	O08784;H3BL37;H3BIX0;F6	26.0545	25.2028	26.3843	26.8496	24.0388	24.3172	23.7486	26.2183	26.6357	21	21	21	20.5	20.5	20.5	135	0	1E+09	89	0.35097	0.812008	0.131707	0.346317
Tcp1	T-complex protein 1 subunit alpha	P11983;P11983	P11983;P11983-2;F2Z483	21.4723	22.3513	23.7491	24.2621	17.5	23.2387	17.5	17.5	22.9436	4	4	4	10.1	10.1	10.1	60.448	0	84433000	13	0.144245	0.857289	0.764594	3.20971
Tgm1	Protein-glutamine gamma-glutamyl	Q9JLF6;A0A0R4J	Q9JLF6;A0A0R4J293	23.6706	23.0266	25.1865	23.1828	23.7581	22.5422	17.5	17.5	22.1444	4	4	4	6.1	6.1	6.1	89.825	0	1.62E+08	10	0.475371	0.80024	1.36957	4.9131
Tgm3	Protein-glutamine gamma-glutamyl	Q08189	Q08189	21.5731	23.0408	17.5	20.2622	19.5287	18.2164	17.5	22.8295	21.6611	1	1	1	1.9	1.9	1.9	77.308	0	68328000	5	0.318184	1.36886	0.00581684	0.0410824
Tgtp;Tgtp2		Q62293;Q3T9E4	Q62293;Q3T9E4	17.5	17.5	17.5	23.0489	17.5	17.5	17.5	17.5	21.8667	1	1	1	2.4	2.4	2.4	47.12	0.00404	12502000	1	0.427243	-1.84964	0.427243	-1.45555
Thrap3	Thyroid hormone receptor-associate	Q569Z6;Q8BZN7	Q569Z6;Q8BZN7	17.5	21.2561	22.4751	20.3666	22.5355	19.8147	17.5	22.9854	24.8475	4	4	4	6.7	6.7	6.7	108.18	0	1.56E+08	15	0.104154	-0.495192	0.197211	-1.36724
Tkt	Transketolase	P40142	P40142	21.2969	22.249	21.2571	23.3727	22.6942	22.7089	17.5	22.0534	23.7857	3	3	3	6.4	6.4	6.4	67.63	0	65793000	6	1.54891	-1.32424	0.0914054	0.487967
Tln1	Talin-1	P26039;F6S1V7;	P26039	21.962	24.9984	24.1041	25.1908	26.1645	25.7038	19.892	22.7059	25.7669	16	16	16	9.8	9.8	9.8	269.82	0	3.82E+08	46	0.992891	-1.99821	0.177945	0.899869
Tmem184c		D3YVG4	D3YVG4	17.5	24.0343	21.5038	20.88	26.5899	25.7933	26.5619	25.0123	23.8837	1	1	1	3.5	3.5	3.5	26.656	1	5.18E+08	11	0.582612	-3.40836	0.942557	-4.13994
Tmpo	Lamina-associated polypeptide 2, is	Q61029;Q61029	Q61029;Q61029-3;Q61033	22.1263	22.8808	24.6129	17.5	17.5	23.003	17.5	22.4221	25.1513	6	6	6	18.6	18.6	18.6	50.372	0	1.26E+08	15	0.914828	3.87235	0.255565	1.51554
Top1	DNA topoisomerase 1	Q04750	Q04750	17.5	17.5	17.5	17.5	17.5	17.5	17.5	17.5	17.5	2	2	2	3.3	3.3	3.3	90.875	0	4032500	1	-4.34E-08	0	-4.34E-08	0
Top2a	DNA topoisomerase 2-alpha	Q01320	Q01320	21.0739	20.8317	17.5	21.2657	17.5	20.3619	17.5	20.8049	21.1347	4	4	4	3.9	3.9	3.9	172.79	0	27745000	5	0.019064	0.0927111	0.00225893	-0.0113163
Tpi1	Triosephosphate isomerase	P17751;H7BXC3	P17751;H7BXC3	20.5747	22.9631	23.1314	23.1426	17.5	17.5	17.5	17.5	22.4515	4	4	4	15.4	15.4	15.4	32.191	0	39393000	6	0.622219	2.8422	0.766332	3.07255
Tpm3;Tpm3-rs7	Tropomyosin alpha-3 chain	P21107-2;D3Z6I	P21107-2;D3Z6I8;E9Q7Q3;	24.6449	24.4745	24.5151	24.9824	22.2784	25.1271	23.4668	25.7527	26.6775	13	13	8	52.8	52.8	34.7	29.02	0	6.2E+08	36	0.169119	0.415511	0.32409	-0.754157
Tpm4	Tropomyosin alpha-4 chain	Q6IRU2	Q6IRU2	20.9477	17.5	17.5	22.8873	17.5	22.2147	17.5	17.5	24.1782	5	4	4	23.4	19	19	28.467	0	1.01E+08	9	0.46897	-2.2181	0.161492	-1.07683
Tpt1	Translationally-controlled tumor pr	P63028	P63028	17.5	21.2231	17.5	23.3903	17.5	24.0362	17.5	17.5	23.9987	1	1	1	8.1	8.1	8.1	19.462	0	62291000	6	0.527176	-2.90113	0.136835	-0.925193
Tra2a	Transformer-2 protein homolog alp	A0A0N4SVC2;Q6	A0A0N4SVC2;Q6PFR5;E9QF	21.0607	21.3042	22.5856	21.6336	17.5	20.7462	17.5	17.5	21.689	2	1	1	8.6	5	5	32.259	0.002342	28327000	7	0.558355	1.69024	0.86917	2.75384
Tra2b	Transformer-2 protein homolog bet	P62996;F8WJG3	P62996;F8WJG3	22.039	17.5	17.5	23.0915	17.5	21.9753	17.5	17.5	22.5917	4	4	3	16	16	12.5	33.665	0	53151000	5	0.332823	-1.84259	0.0271927	-0.184252
Trim28	Transcription intermediary factor 1-	Q62318	Q62318	17.5	17.5	17.5	17.5	20.0282	25.2084	17.5	17.5	17.5	2	2	2	4.1	4.1	4.1	88.846	0	57026000	2	0.684011	-3.41221	-4.34E-08	0
Try4;Try5		Q9R0T7;Q9QUK9	Q9R0T7;Q9QUK9	26.4558	27.1231	26.8861	27.4283	24.6474	24.4576	24.5097	25.5377	27.5896	1	1	1	8.1	8.1	8.1	26.274	0.003922	1.27E+09	20	0.598546	1.31055	0.436147	0.942684
Tuba1a;Tuba1c;Tuba3a	Tubulin alpha-1A chain;Tubulin alpl	P68369;P68373	P68369;P68373;P05214	26.0763	27.8083	28.3866	27.9513	28.7514	29.5972	27.4631	27.8647	28.7966	14	14	1	41	41	2.9	50.135	0	3.97E+09	176	0.73138	-1.34288	0.316598	-0.61771
Tuba1b;Tuba4a	Tubulin alpha-1B chain;Tubulin alpl	P05213;P68368	P05213;P68368;A0A0A0M	20.1802	18.0953	20.6063	23.3268	23.3542	17.5	17.5	17.5	22.3839	14	1	1	41	2.9	2.9	50.151	0.004228	55549000	3	0.349925	-1.76639	0.0993062	0.4993
Tubb2b;Tubb2a	Tubulin beta-2B chain;Tubulin beta-	Q9CWF2;Q7TMN	Q9CWF2;Q7TMM9	17.5	17.5	17.5	23.9677	17.5	22.5192	20.978	17.5	17.5	9	1	1	26.7	3.4	3.4	49.953	0	31184000	2	0.912207	-3.82896	0.427243	-1.15933
Tubb4b;Tubb4a	Tubulin beta-4B chain;Tubulin beta-	P68372;Q9D6F9	P68372;Q9D6F9	22.536	24.0546	24.7857	24.8092	22.5296	25.6075	21.6536	22.3446	23.8301	13	4	3	32.4	10.3	7	49.83	0	1.86E+08	12	0.1747	-0.523376	0.569984	1.18267
Tubb5	Tubulin beta-5 chain	P99024;A2AQ07	P99024	26.282	27.6098	28.6581	29.1626	28.3066	29.2521	27.3788	27.8409	29.0398	14	14	4	38.5	38.5	13.1	49.67	0	4.16E+09	173	0.861319	-1.39047	0.269202	-0.569843
Tubb6	Tubulin beta-6 chain	Q922F4	Q922F4	17.5	17.5	21.8222	21.4715	17.5	25.2185	17.5	17.5	21.4147	10	5	3	31.3	16.6	11.9	50.09	0	57226000	6	0.390311	-2.45594	0.0233591	0.135858
Txn	Thioredoxin	P10639	P10639	22.9364	25.3062	26.0777	25.2466	24.6474	17.5	24.5802	24.9796	25.5691	4	4	4	31.4	31.4	31.4	11.675	0	4.16E+08	15	0.361837	2.30877	0.0977391	-0.26952
Txnl1	Thioredoxin-like protein 1	Q8CDN6	Q8CDN6	17.5	21.4276	17.5	21.4541	21.3858	21.5498	17.5	17.5	22.0256	2	2	2	12.8	12.8	12.8	32.237	0.004158	21483000	3	0.947912	-2.65402	0.0337105	-0.199315
U2surp	U2 snRNP-associated SURP motif-co	Q6NV83;Q6NV8	Q6NV83;Q6NV83-3;Q6NV8	21.2484	21.5903	17.5	22.3502	17.5	17.5	17.5	17.5	22.7911	3	3	3	6	6	6	118.26	0	35234000	6	0.182341	0.996159	0.143395	0.849202
Uba1	Ubiquitin-like modifier-activating e	Q02053;P31254	Q02053	21.6098	24.7303	25.085	26.136	24.1409	23.4501	21.2447	25.9157	25.2044	7	7	7	10.2	10.2	10.2	117.81	0	3.29E+08	25	0.218661	-0.767296	0.0594557	-0.313218
Ugdh	UDP-glucose 6-dehydrogenase	O70475	O70475	21.2606	22.0288	17.5	22.4312	17.5	17.5	17.5	21.4344	21.0188	2	2	2	6.7	6.7	6.7	54.831	0	24353000	4	0.199668	1.11937	0.0510867	0.278706
Upp2	Uridine phosphorylase 2	Q8CGR7;Q8R093	Q8CGR7;Q8R093	23.6095	27.681	29.3313	26.6239	17.5	23.598	21.2228	25.0279	24.5371	1	1	1	4.1	4.1	4.1	35.755	0	1.67E+09	63	0.606816	4.29998	0.721568	3.27802
Vars	Valine--tRNA ligase	Q9Z1Q9;G3UY93	Q9Z1Q9;G3UY93;G3UZ22	17.5	19.5406	17.5	20.4685	17.5	17.5	21.8568	17.5	21.8356	2	2	2	2.1	2.1	2.1	140.21	0	19936000	2	0.0918181	-0.309298	0.623086	-2.21726
Vcl	Vinculin	Q64727	Q64727	24.0139	26.005	26.3952	25.7744	24.7959	25.2586	22.5822	23.8028	24.6034	11	11	11	12.4	12.4	12.4	116.72	0	4.31E+08	34	0.0877679	0.195105	0.894325	1.80857
Vcp	Transitional endoplasmic reticulum	Q01853	Q01853	22.9803	24.0158	25.0941	26.5214	22.6907	24.1058	22.8563	23.1095	26.4435	10	10	10	17.2	17.2	17.2	89.321	0	4.21E+08	33	0.116844	-0.409258	0.0273134	-0.106373
Vdac1	Voltage-dependent anion-selective c	Q60932-2;Q609	Q60932-2;Q60932;F2Z471	17.5	23.0082	25.047	23.9168	23.037	22.749	22.316	21.0184	22.7277	5	5	5	20.5	20.5	20.5	30.755	0	95985000	13	0.238677	-1.38254	0.0244508	-0.168958
Vdac2	Voltage-dependent anion-selective c	Q60930;G3UX26	Q60930;G3UX26	17.5	17.5	24.4814	22.6641	23.2587	22.932	21.878	17.5	23.8242	2	2	2	10.8	10.8	10.8	31.732	0	79795000	9	0.599284	-3.12447	0.155438	-1.24026
Vim	Vimentin	P20152;A0A0A6	P20152;A0A0A6YWC8	28.242	28.8477	29.1932	29.1244	28.7808	29.6321	26.3607	29.0491	30.3595	43	42	39	81.3	81.3	79.4	53.687	0	6.56E+09	402	0.489471	-0.418131	0.0485275	0.171187
Wdr96		E9Q7R9	E9Q7R9	17.5	17.5	23.1112	17.5	17.5	17.5	17.5	17.5	17.5	1	1	1	1	1	1	193.42	0	13988000	2	0.427243	1.87039	0.427243	1.87039
Xpo1	Exportin-1	Q6P5F9	Q6P5F9	17.5	18.274	22.808	23.938	17.5	20.9445	17.5	17.5	23.5433	2	2	2	2.8	2.8	2.8	123.09	0.002304	53422000	6	0.195386	-1.26684	0.0016144	0.0128994
Ybx1	Nuclease-sensitive element-binding	P62960;A2BGG7	P62960;A2BGG7	22.1011	21.494	24.3609	22.4298	17.5	22.5409	20.9971	24.0923	25.7233	7	7	7	51.2	51.2	51.2	35.73	0	2.45E+08	18	0.414355	1.82845	0.227603	-0.952253
Ywhab	14-3-3 protein beta/alpha;14-3-3 pr	Q9CQV8;Q9CQV8	Q9CQV8;Q9CQV8-2;A2A5N	17.5	17.5	17.5	17.5	17.5	21.8533	17.5	17.5	23.2647	2	1	1	9.3	5.7	5.7	28.086	0	20882000	1	0.427243	-1.4511	0.427243	-1.92157
Ywhae	14-3-3 protein epsilon	P62259;D6REF3	P62259;D6REF3;F6WA09	22.2534	24.0249	24.3017	24.0181	23.2217	22.1094	21.7384	23.3473	24.8654	4	4	4	18.8	18.8	18.8	29.174	0	1.52E+08	7	0.18472	0.410313	0.0659641	0.209651
Ywhag	14-3-3 protein gamma;14-3-3 prote	P61982	P61982	22.0763	17.5	17.5	17.5	17.5	17.5	17.5	17.5	17.5	1	1	1	5.7	5.7	5.7	28.302	0.003945	8323800	1	0.427243	1.52543	0.427243	1.52543
Ywhah	14-3-3 protein eta	P68510	P68510	21.8509	17.5	17.5	17.5	21.0491	21.1108	17.5	23.0026	23.2204	3	3	3	17.1	17.1	17.1	28.211	0	37057000	3	0.190922	-0.936337	0.411058	-2.29069
Ywhaq	14-3-3 protein theta	P68254;P68254	P68254;P68254-2;F6YY69;	21.4313	21.5822	22.5331	22.7581	17.5	17.5	17.5	17.5	23.5544	3	2	2	17.6	13.9	13.9	27.778	0	33885000	5	0.658008	2.59618	0.496882	2.33075
Ywhaz	14-3-3 protein zeta/delta	P63101;D3YXN6	P63101;D3YXN6	24.8351	24.882	26.1756	25.4769	24.3067	24.5921	22.2762	24.3566	26.0821	10	10	9	42	42	38.4	27.771	0	5.03E+08	33	0.376849	0.505677	0.374905	1.05929
Zc3h18	Zinc finger CCCH domain-containing	Q0P678;H3BJJ9;	Q0P678;H3BJJ9;H3BIW0;G	17.5	17.5	17.5	17.5	17.5	19.4332	17.5	17.5	20.438	1	1	1	1.6	1.6	1.6	105.69	0.003817	12235000	1	0.427243	-0.644409	0.427243	-0.979322
Znf143;Znf76;Zfp523	Zinc finger protein 143;Zinc finger p	O70230;O70230	O70230;O70230-3;O70230	17.5	23.8226	23.3284	17.5	17.5	17.5	23.3551	17.5	23.0529	2	2	2	3.4	3.4	3.4	69.04	0.005671	81774000	4	0.932654	4.05034	0.0299463	0.247639

Zyx	Zyxin	Q7TQE2;Q62523	Q7TQE2;Q62523;A0A0N4S\	24.123	24.6724	23.0673	25.0872	17.5	24.1897	20.9685	24.8638	25.4445	4	4	4	10.7	10.7	10.7	57.026	0	2.89E+08	14	0.279552	1.69526	0.045019	0.195285
	UPF0444 transmembrane protein C	Q9DAM7	Q9DAM7	17.5	17.5	24.4654	24.3808	17.5	21.7552	17.5	17.5	23.2242	1	1	1	22.6	22.6	22.6	11.549	0.002222	90384000	4	0.1714	-1.3902	0.047135	0.413747
		A0A140LIJ4	A0A140LIJ4	17.5	22.6036	23.4242	17.5	17.5	21.3576	21.7948	22.418	17.5	2	2	2	0.6	0.6	0.6	516.94	0.004049	43918000	1	0.457096	2.39006	0.0891578	0.604989
